# Tough, thermal insulating and scalable puff pastry-inspired architected cementitious material via simple rolling-folding

**DOI:** 10.1038/s41467-026-77120-z

**Published:** 2026-08-27

**Authors:** Kailun Xia, Yuning Chen, Xingzi Liu, Siyuan Qiu, Qian Lin, Yi Tang, Tiantian Xiang, Sule Chen, Enlai Dong, Zijian Jia, Wei Wang, Yu Chen, Yamei Zhang

**Affiliations:** 1 State Key Laboratory of Engineering Materials for Major Infrastructure, School of Materials Science and Engineering, Southeast University, Nanjing, China; 2 Hefei National Research Center for Physical Sciences at the Microscale, University of Science and Technology of China, Hefei, China; 3 College of Civil Engineering, Nanjing Tech University, Nanjing, China

**Keywords:** Mechanical properties, Ceramics, Composites, Civil engineering

## Abstract

Architected material design enables cementitious materials to achieve highly desirable mechanical and functional performance for next-generation infrastructure. However, current architected cementitious materials require complex and energy-intensive fabrication, limiting their scalability and large-scale implementation. Inspired by the preparation of puff pastry, we propose a lamellar cementitious architected material fabricated by a simple rolling-folding process under ambient conditions. This material features a highly ordered lamellar architecture, consisting of alternating parallel cement lamellae and discrete plate-like cavities. This structure enables ductile fracture behavior, high fracture toughness, and effective thermal insulation. The specific fracture toughness of this material reaches up to 91 MPa·mm^0.5^/g·cm^3^, approximately 20 times higher than that of the cast counterpart, surpassing conventional reinforced cementitious composites and approaching the level of certain polymers. Meanwhile, its thermal conductivity decreases to 0.38 W/m·K, representing a 68.3% reduction relative to the cast counterpart, and outperforming lightweight cementitious materials with comparable densities. Furthermore, this material demonstrates good manufacturing efficiency, scalability, and geometric adaptability, overcoming the application limitations of existing architected cementitious materials. This study can help advance the development of next-generation infrastructure materials.

## Abstract (web summary)

Inspired by puff pastry, this research highlights a lamellar cementitious material that combines high mechanical toughness, scalable fabrication and effective thermal insulation, offering a route toward safer and more energy-efficient infrastructure.

## Introduction

Cementitious materials have been, and will likely remain for a long time, the primary materials for infrastructure construction worldwide^1,2^. However, owing to the intrinsically low toughness and poor energy dissipation capacity of cementitious materials, conventional concrete structures are prone to unstable crack propagation and brittle failure under extreme events, including earthquakes, impacts, and accidental loads. Such failure mechanisms limit their ability to withstand sudden energy release and may result in progressive damage or catastrophic collapse, thereby threatening the safety and resilience.^3,4^

Although modern concrete can be toughened with steel reinforcement, fibers, or polymers, these methods still provide only modest and limited improvements, with enhancements in ductility and toughness generally remaining below a twofold increase^5,6^. In addition, most urban concrete buildings rely on energy-intensive air conditioning systems for indoor temperature regulation and thermal management, contributing substantially to energy consumption and carbon emissions^7,8^. Lightweight foamed concrete presents a promising route to improving thermal insulation but often fails to retain adequate mechanical performance^9,10^.

Nature-inspired and architected designs have been promising strategies for achieving desirable mechanical and functional properties of structural materials^11–18^. More recently, these concepts have endowed cementitious materials with remarkable improvements in ductility, toughness and thermal insulation^5,6,19–21^. These strategies are primarily accomplished by creating hierarchically ordered structures^6,22–25^, designing and arranging weak interfaces^5,22,24,26^, and adopting lamellar distributions of organic–inorganic components^5,6,20,25^ to mimic structures found in natural materials such as nacre^27–29^, bamboo^21^, fish scales^30,31^ and bone^32,33^. The improvements in toughness are realized through mechanisms such as defect-induced crack twisting and deflection^5,21,30,34^, energy dissipation via deformation of organic phases^5,6,21^, as well as lamellar sliding and pull-out^5,12^, while the enhancements in thermal insulation primarily originate from orderly arranged thermal conducting barriers^6,17^.

However, existing high-performance architected cementitious materials often rely heavily on complex fabrication methods, such as 3D printing^22,24^, ice-templating^6,20^, hot pressing^21^, laser-cutting^5^ and prefabricated mold casting^26^. These methods not only require considerable time but also involve processes such as low-temperature cooling, heating, vacuum impregnation, and freeze–thaw cycling, thereby imposing significant constraints on the scalability, formability, production efficiency, and energy consumption of materials fabrication. Therefore, cementitious architected materials are still facing challenges in achieving facile, flexible and efficient large-scale fabrication, which must be addressed to meet the demands of infrastructure-scale construction.

In recent years, designs of hydrogel–cementitious composites have shown great compatibility and potential in toughness-enhancement strategies for cement-based materials, by enabling the creation of layered structures, increasing the energy-absorption capacity of the matrix, and improving the material’s strain capacity^6,35,36^. However, current approaches face challenges in simultaneously achieving adequate performance improvements, mild processing conditions, and good scalability, and are therefore largely restricted to laboratory-scale studies, hindering large-scale implementation.

In this work, through the utilization of hydrogel, we developed a puff pastry architected cementitious material (PPAC) featuring a well-aligned lamellar architecture composed of alternately distributed solid phases and lamellar cavities at the micrometer scale. The preparation of PPAC requires no hot pressing, freezing, or molding processes and can be carried out entirely under ambient conditions. Meanwhile, it demonstrates excellent scalability and geometric adaptability, enabling the successful fabrication of components with large dimensions and diverse geometries. By inducing crack deflection and mechanical interlocking, the PPAC architecture effectively enhances the fracture toughness of the material without compromising its specific strength. Simultaneously, the PPAC architecture imparts pronounced thermal insulation to the material, which is also a highly desirable property in infrastructure engineering. This endows PPAC with multifunctionality and thermal management advantages in addition to its excellent structural performance.

As a result, while maintaining a specific flexural strength comparable to that of conventional cast cementitious materials, PPAC simultaneously achieves a specific fracture toughness of more than 90 MPa·mm^0.5^/g·cm^3^ and a thermal conductivity of 0.38 W/m·K, which are approximately 20 times higher and 68.3% lower compared to ordinary cast cement paste, respectively. The fracture toughness far surpasses that of conventional fiber-reinforced and steel-reinforced concretes^4,37–40^, reaching levels comparable to polymers^41–46^ and certain advanced technical ceramics^47–51^, while the thermal conductivity remains significantly lower than that of lightweight concretes with comparable density^52–55^. These findings demonstrate the strong application potential of this PPAC material for next-generation large-scale resilient infrastructure, combining excellent structural performance, efficient thermal management, and facile fabrication under mild conditions.

## Results

### Fabrication strategy

**Fig. 1:**
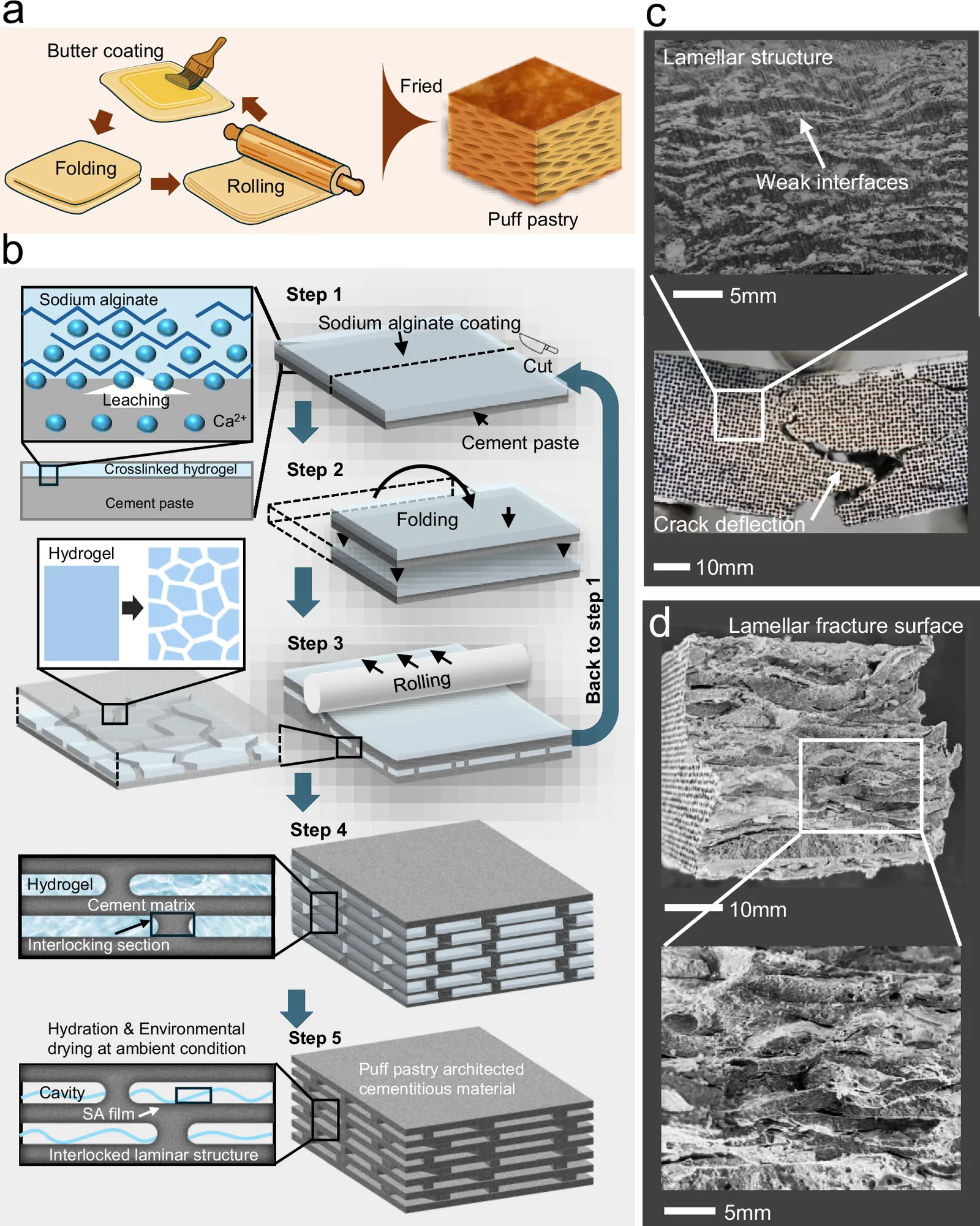
Fabrication and fracture features of puff pastry architected cementitious material (PPAC). **a** Schematic illustration of puff pastry fabrication process. Traditional preparation process involves repeated steps such as coating with butter, folding, and rolling, followed by frying to produce the final puff pastry. **b** Schematic illustration of rolling-folding method for manufacturing PPAC. The process involves repeated cycles of coating with sodium alginate, folding, and rolling, followed by drying and hardening to form PPAC. **c** Lamellar weak interfaces observed from the side view of the PPAC specimen and crack deflection behavior observed in PPAC specimen during 3-point bending (3PB) test. **d** Fracture surface with lamellar structure.

Our fabrication strategy was inspired by the manufacturing process of puff pastry in food engineering, which employs a very simple yet highly effective method to generate well-ordered lamellar structures (Fig. 1a). In this process, the dough is repeatedly folded and rolled, while butter is applied to prevent adhesion between layers, thereby forming a lamellar architecture. During frying, the butter within the dough melts and is removed, ultimately producing the layered puff pastry architecture. This rolling-folding process resembles the accumulative roll bonding (ARB) technique used in advanced metallic materials for fabricating advanced multilayered metals^56–58^. Here, we adopt this concept for the fabrication of puff pastry architected cementitious materials (Fig. 1b). With proper mix design, the cementitious material can exhibit dough-like plasticity during its plastic stage (Suppl. Figs. 1–2). Meanwhile, sodium alginate (SA) is employed as the butter to prevent interlayer bonding between adjacent cement lamellae. SA is a low-cost, non-toxic bio-extracted polysaccharide capable of crosslinking with di- and tri-valent metal ions, especially calcium ions, to form stable and water-insoluble hydrogels^59–63^. Owing to the abundant release of calcium ions from the surface of fresh cement paste, SA can rapidly and spontaneously crosslink on the surface without the need for any additional treatment^64,65^ (Step 1 in Fig. 1b, Suppl. Figs. 3–4). This crosslinked hydrogel provides the basis for preventing interlayer bonding in the cement paste (Suppl. Fig. 5) and for creating well-ordered weak interfaces. Based on this, lamellar cement-based materials can be created by rolling, cutting, folding, and repeating this process with cement paste coated with the alginate-based hydrogel (Steps 1 ~ 3 in Fig. 1b). Due to its “egg-box” crosslinked network^66^ (Suppl. Fig. 4) and its highly hydrated state (water content > 90%, Suppl. Fig. 3f), the alginate-based hydrogel generated on the surface of cement paste possesses limited deformability (Suppl. Figs. 6–7). As a result, it can fracture with the in-plane elongational deformation of plastic cement paste during repeated rolling processes (Step 3 in Fig. 1b, Suppl. Fig. 8), creating regions that allow mechanical interlocking between adjacent cement paste layers, thereby maintaining the overall integrity of the manufactured material (Step 4 in Fig. 1b, Suppl. Figs. 9). With repeated rolling-folding cycles, the number of lamellae in the material increases exponentially, and a highly pronounced lamellar structure can be formed after only a few repetitions of this process (the maximum number of folds in this study was seven). After that, the hydrogel lamellae in the material gradually dries under environmental drying and water absorption of the cement paste to form thin films, leaving sheet-like cavities and ultimately producing a puff pastry architected cementitious material (PPAC) (Step 5 in Fig. 1b). Under ambient conditions, after seven folding cycles, we obtained PPAC which exhibited evident multilayered structure with embedded discrete weak interfaces (Fig. 1c). The PPAC exhibited significant crack deflection capability (Fig. 1c), and the fracture surface also showed a clear lamellar structure (Fig. 1d).

### Mechanical properties

**Fig. 2:**
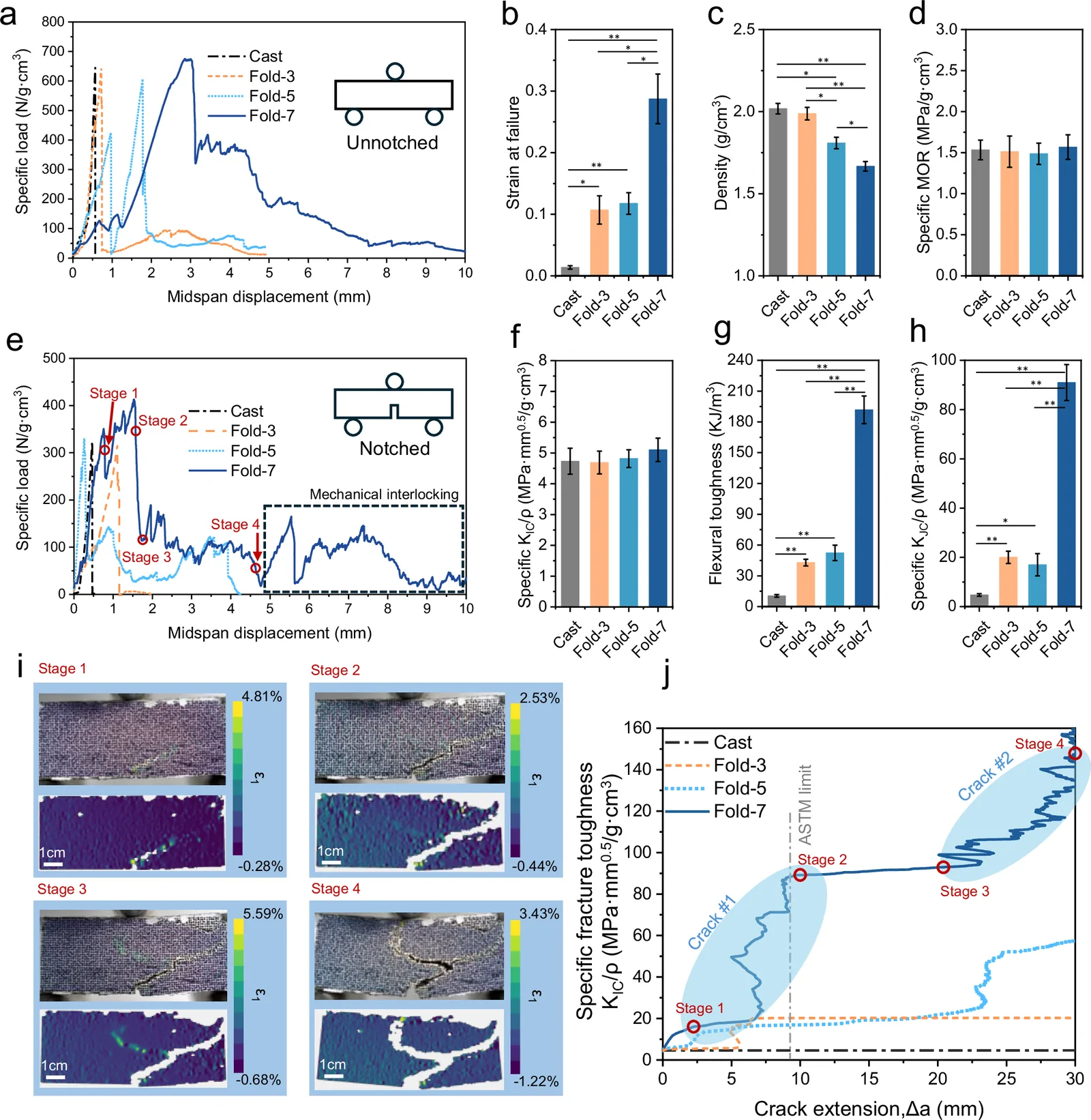
Mechanical properties of puff pastry architected cementitious material (PPAC). **a** Representative 3-point bending (3PB) curves of the PPAC with different numbers of rolling-folding cycles and cast specimens. **b**, **d**, **f**, **g**, **h** Comparison of specific mechanical parameters between PPAC with different numbers of rolling-folding cycles and cast specimens. Absence of significance mark means no significant difference between results. **c** Density of PPAC and cast specimens. **e** Representative SENB test curves of the PPAC with different numbers of rolling-folding cycles and cast specimens. **i** Representative crack extension in Fold-7 specimen during SENB test captured by digital image correlation (DIC) technique. **j** Representative crack-resistance curves (J-R curves) for manifesting the resistance to fracture, *K*_*J*_, in terms of the stress intensity, as a function of crack extension, *Δa*, for the PPAC with different numbers of rolling-folding cycles and cast specimens. The gray dashed line in the figure denotes the maximum crack-extension limit specified in ASTM E1820. The values corresponding to this limit on the curves were used to calculate the fracture toughness *K*_*JC*_. Data in the bar charts are presented as mean ± SD from three independent specimens (*n* = 3). * depicts *p* < 0.05 and ** depicts *p* < 0.01, which indicate statistically significant differences. *P*-value is obtained from T-test. Source data are provided as a Source Data file.

Compared with the cast counterpart, PPAC specimens exhibit crack deflection and ductile fracture (Fig. 2). As rolling–folding without SA does not significantly alter the mechanical response (Suppl. Fig. 10), these features are mechanistically attributed to the weak interfaces introduced by the crosslinked alginate-based hydrogel. Consequently, the toughness of PPAC is enhanced under bending perpendicular to the interfaces (see parallel loading results in Suppl. Fig. 11), and is further amplified with the increasing number of interfaces generated by repeated rolling–folding. Specifically, in 3-point bending (3PB) results of unnotched specimens, the Fold-7 (representing PPAC specimen with a rolling–folding cycle number of 7) specimen exhibited a markedly different mechanical response from conventional cast cement paste, characterized by stepwise, tortuous, and non-brittle behavior (Fig. 2a). In contrast, this feature was significantly weakened in Fold-5 and Fold-3, confirming that the number of internal lamellar structures in PPAC is a critical factor influencing its mechanical performance. The failure of Fold-7 specimen after peak load was significantly delayed, and multiple subsequent load rises and drops were observed, demonstrating progressive and multi-stage fracture characteristics and gradual failure (Fig. 2a). This resulted in excellent ductility enhancement: compared to cast specimens, the strain at failure of Fold-7 reached approximately 20 times that of the cast counterpart (Fig. 2b).

Moreover, although the density of PPAC specimens decreased noticeably with increasing number of rolling-folding cycles (density of the Fold-7 is 17.2% lower than the Cast) (Fig. 2c), the specific modulus of rupture (specific MOR) was not significantly affected and remained comparable to that of cast specimen (Fig. 2d). This may be related to the material’s crack initiation behavior. Unlike brick-mortar structural designs which consist of continuous weak interfaces and discrete solid phases, PPAC consists of discrete weak interfaces and interlocked solid phases, which allow crack initiation to still occur within the solid cementitious phase. Meanwhile, single-edge notch bending (SENB) tests (Fig. 2e) characterizing the fracture toughness at crack initiation indicated that PPAC specimens with different numbers of rolling-folding cycles, as well as cast specimens, maintained similar *K*_*IC*_ levels (Fig. 2f), confirming this point again. Similar mechanisms have been reported in a study on bone-inspired cementitious architected materials, where performance enhancement was also induced by void-guided structural design^26^. These results suggest that PPAC can achieve enhanced ductility and fracture toughness without sacrificing its specific strength. This resulted in a flexural toughness of 190 kJ/m³ for Fold-7, approximately 15 times that of the cast specimen (Fig. 2g).

In SENB results, cast specimens exhibited brittle fracture at lower stress levels compared to 3PB results due to the presence of prefabricated notch. However, PPAC specimens still showed ductile fracture behavior similar to that observed in unnotched 3PB tests (Fig. 2 e, Suppl. Movie 1). Further analysis of crack extension using digital image correlation (DIC) revealed the multistage characteristics of the load-displacement curves and the corresponding crack paths (Fig. 2 i). Fold-7 still exhibited the toughest fracture behavior and is presented here as a representative specimen. The full results for Fold-7, Fold-5 and Fold-3 are provided in the Suppl. Figs. 12–14. Specifically, in Fold-7, the initial crack propagation occurred with significant deflection along the notch (Stage 1 in Fig. 2e, i). Subsequently, Fold-7 specimen did not fail instantaneously but displayed multiple load rises and drops as indicated by the SENB curve. This process occurred during the propagation of the #1 primary crack and concluded when the crack fully penetrated the specimen (Stage 2 in Fig. 2e, i). This indicates that the #1 primary crack traversed multiple weak interfaces, causing progressive and sequential fracture with multiple small-scale deflections. Thereafter, the Fold-7 specimen still did not fail, instead, a #2 crack branched from the stress-concentrated region along the #1 crack path, producing further gradual failure during propagation (Stage 3 in Fig. 2e, i). During this process, the load gradually decreased until the crack fully penetrated the specimen (Stage 4 in Fig. 2e, i). However, the specimen could still maintain overall integrity while the load fluctuated within a certain range (dashed box in Fig. 2e). After that, the specimen continued to undergo ductile deformation, due to interlayer mechanical interlocking (will be discussed later). Mechanical interlocking and interlayer friction provide resistance to lamellar pull-out, enabling continued ductile response. This behavior resembles the lamellar pull-out effect in nacre and shell-inspired biomimetic structures^12^.

The crack-resistance curve (J-R curve) fully captures the evolution of specific fracture toughness with crack extension (Fig. 2j). Cast specimen exhibited immediate brittle failure after crack initiation, with K_J_ remaining constant at the initial *K*_*IC*_. In contrast, PPAC showed a J-R curve with ascending stage similar to tough biomimetic materials with nacre-like structures. Notably, Fold-7 exhibited two pronounced ascending stages in the J-R curve, aligning well with the two crack propagation events observed via DIC analysis and SENB tests. This strongly demonstrates the excellent crack resistance of Fold-7 during crack propagation. According to the ASTM limit^67^ for *K*_*JC*_ determination in brittle materials, the specific fracture toughness of Fold-7 reached approximately 90 MPa·mm^0.5^/g·cm³, about 22 times that of cast specimen (Fig. 2h).

However, it should be noted that although the results demonstrate improved mechanical performance with increasing rolling-folding cycles, this study only presents results up to a maximum of 7 folds. This limitation arises from the significant deterioration in processability beyond seven cycles, making it difficult to maintain a stable rolling–folding process with the equipment used in this work. In principle, PPAC is expected to exhibit a performance limit associated with the number of cycles for specific mixtures. However, this remains to be systematically investigated in the future.

### Thermal insulation performance

**Fig. 3:**
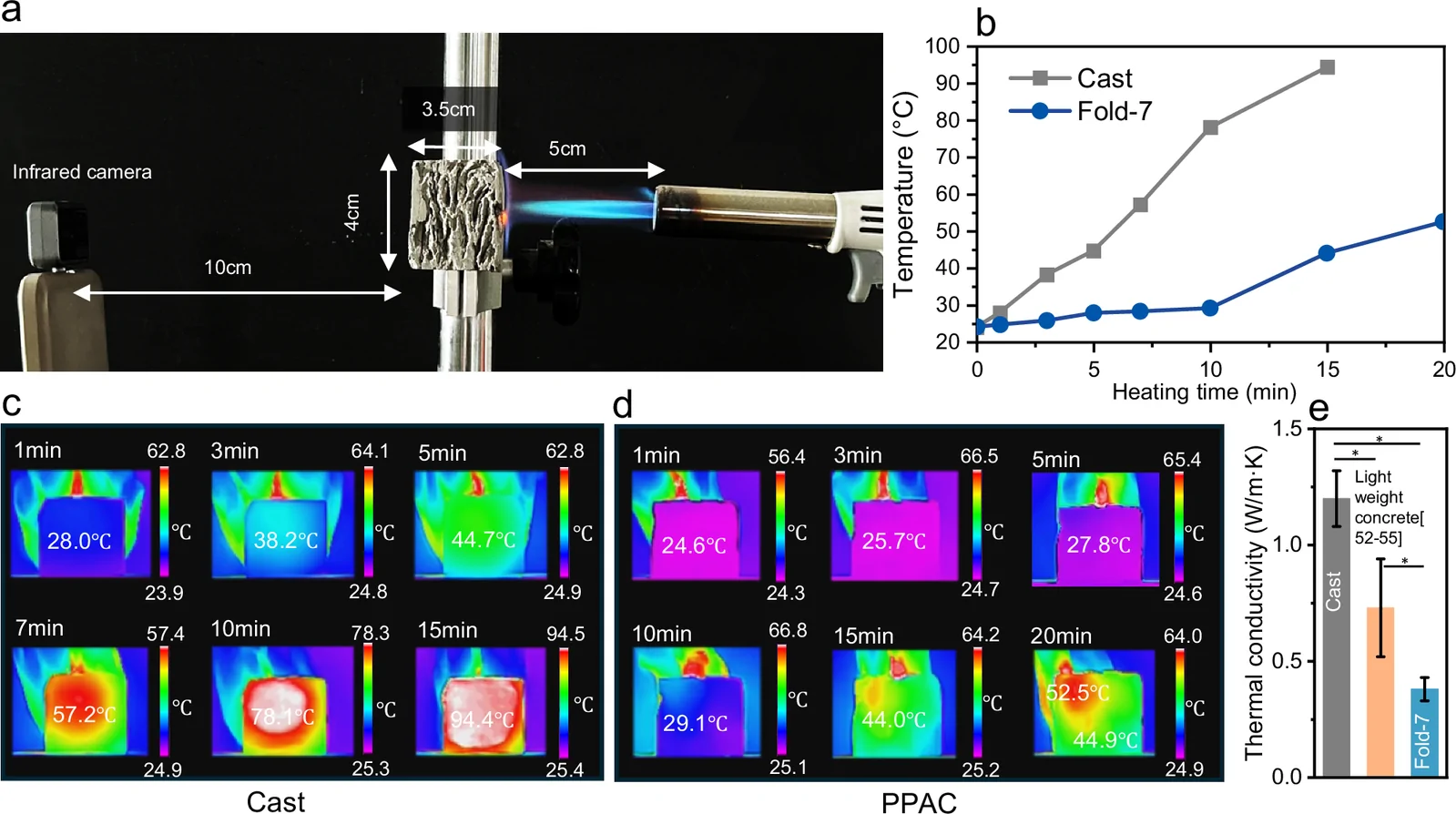
Thermal insulation performance of puff pastry architected cementitious material (PPAC). **a** Illustration of the fire & thermal resistance test configuration. The torch used was a butane flame torch, with a flame core temperature of approximately 1300 °C. **b** Representative maximum temperature evolution curves of specimen’s backside. **c**, **d** Representative thermal infrared images of the specimen’s backside during the fire & thermal resistance test. The values shown in the figure represent the maximum temperature in the corresponding regions. **e** Comparison on thermal conductivity of cast specimen, light weight concrete with a density similar to that of PPAC Fold-7, and PPAC Fold-7. The data of lightweight concrete are referenced from the literatures^52–55^. Data in the bar charts are presented as mean ± SD from three independent specimens (*n *= 3). * depicts *p* < 0.05 and ** depicts *p* < 0.01, which indicate statistically significant differences. *P*-value is obtained from T-test. Source data are provided as a Source Data file.

The lamellar structure of PPAC endows it with pronounced thermal anisotropy (Suppl. Fig. 15), and it exhibits excellent thermal insulation performance when the heat flow is perpendicular to the lamellar structure. According to the literature^17^, a blowtorch with a flame temperature of approximately 1300 °C was applied to heat the specimen surface while the backside temperature was monitored using an infrared camera to evaluate the fire & thermal resistance of PPAC Fold-7 in the direction perpendicular to the lamellar structure (Fig. 3a). Specimens with dimensions of 40 mm (height) × 40 mm (width) × 35 mm (thickness) were used during this test. Under continuous heating, the backside temperature of the Fold-7 specimen increased much more slowly than that of the cast specimen (Fig. 3b–d). After 15 min of flame exposure, the maximum backside temperature of the cast specimen reached 94.4 °C (Fig. 3c), whereas that of Fold-7 reached only 44 °C (Fig. 3d). Even after 20 min of heating, the maximum backside temperature of Fold-7 was limited to 52.5 °C (Fig. 3d). It should be noted that this test did not follow a standardized procedure and was intended solely as a qualitative demonstration to visually illustrate the thermal insulation performance of PPAC. Therefore, the more precise and quantitative Hot Disk measurements were conducted, and the results revealed that Fold-7 exhibited a thermal conductivity of only 0.38 W/m·K, representing a 68.3% reduction compared with the cast specimen (1.2 W/m·K) (Fig. 3e). This thermal conductivity value is also significantly lower than that of lightweight concretes with densities similar to Fold-7^52–55^ (Fig. 3e).

### Material architecture and functional mechanisms

**Fig. 4:**
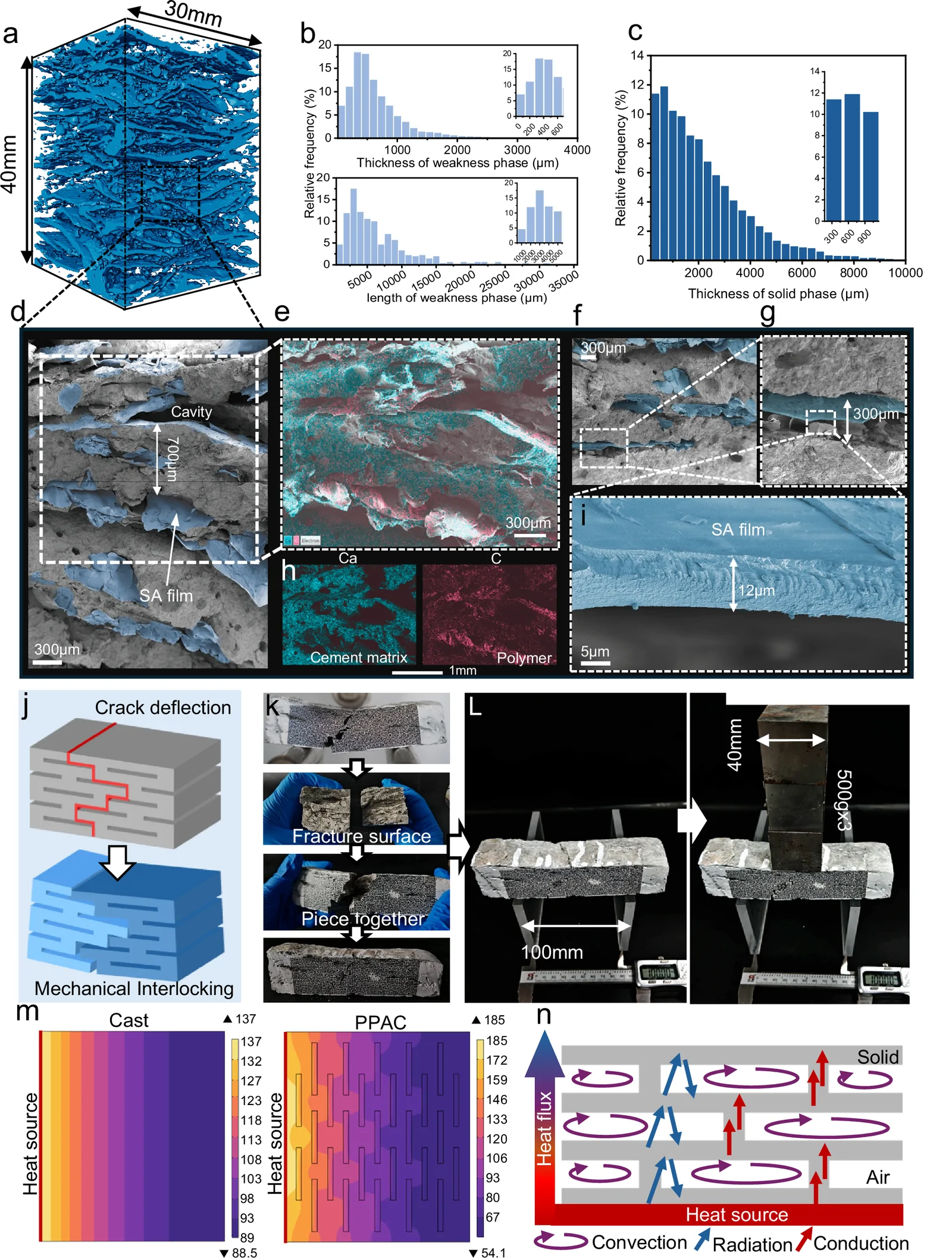
Material architecture and performance mechanisms of PPAC Fold-7. **a** Lamellar structure demonstrated by X-CT reconstruction. This structure is composed of alternating solid phases of the material matrix and weak phases consisting of sheet-like cavities. **b**, **c** Thickness distributions of solid phase and weakness phase and length distributions of weakness phase. Source data are provided as a Source Data file. **d**–**i** Lamellar structure demonstrated by SEM and EDS mapping. The results verify that the alginate hydrogel contributes to the formation of the lamellar structure. To make the results more intuitive, a color-enhanced version is presented here, while the original version can be found in Suppl. Fig. 19. **j** Schematic diagram of toughening mechanisms, including crack deflection and mechanical interlocking. **k**, **L** Mechanical interlocking after fracture. Even after fracture, the PPAC specimen could be reassembled and still retained partial structural integrity and load-bearing capacity. **m** Heat transfer simulation results. The lamellar cavities in PPAC can effectively impede heat transfer. **n** Schematic illustration of the thermal insulation mechanism. The lamellar cavities act as barriers to heat conduction, and the cement–air interfaces provide multiple reflections of infrared radiation.

X-CT results show that after multiple rolling-folding cycles, PPAC Fold-7 exhibits evident lamellar structure, in which solid phases and weakness phases are alternately and parallelly arranged (Fig. 4a). The weakness phases mainly originate from the space occupied by the SA hydrogel. Their thickness is primarily determined by the thickness of the SA hydrogel when it ruptures during the rolling process (Suppl. Figs. 6–8). Consequently, the thickness distributions of the weakness phases are mainly concentrated within a few hundred micrometers and are similar across different rolling-folding cycles (Fig. 4b, Suppl. Fig. 16). By contrast, the thickness distributions of the solid phases is highly dependent on the number of rolling-folding cycles (Suppl. Fig. 16). With increasing number of  rolling-folding cycles, the quantity of the discrete weakness phase increased significantly, while the thickness of the solid phase gradually decreased (Suppl. Figs. 16, 17). In PPAC Fold-7, the most probable thickness of the solid phases falls within 300 ~ 900 μm (Fig. 4c), enabling the construction of lamellar architectures at the micrometer scale via such a simple and ambient rolling-folding approach. Meanwhile, the length of weakness phase is mainly distributed within a range of several millimeters, with the most probable value around 3 mm (Fig. 4b, Suppl. Fig. 16). This indicates that the hydrogel has been sufficiently fragmented after multiple rolling-folding processes, ensuring effective interlocking between the cement paste lamellae and maintaining the overall integrity of the PPAC material.

SEM observations further confirm the lamellar architecture of PPAC Fold-7, revealing a ternary structure consisting of cement matrix, cavity, and sodium alginate film (Fig. 4d–i, Suppl. Fig. 18, Suppl. Table 3). This structure forms because the SA hydrogel undergoes dehydration during cement hydration and environmental drying, leaving polymer films of only ~10 μm in thickness and lamellar cavities of ~300 μm in thickness within the originally occupied regions (Fig. 4f, g, i). Meanwhile, during early-age hydration of cement, as the internal relative humidity decreases, fluid desorption from the SA layer may also promote the diffusion of water from the hydrogel into the cement matrix. This released water may partially mitigate self-desiccation and shrinkage of the cement, thereby providing an internal curing effect, which can also improve the mechanical properties^68–71^. However, as this is not the primary focus of the present study, it is not discussed in detail here.

These lamellar weak interfaces impede the propagation of cracks and induce crack deflection along the interlayers during fracture (Fig. 4 j, Suppl. Fig. 20), resulting in lamellar fracture surfaces (Fig. 1d).

Moreover, since the length of the weakness phases is much larger than the thickness of the solid phases (Fig. 4b), strong mechanical interlocking develops between adjacent cement matrix lamellae at the fracture sites (Fig. 4j). This interlocking effect effectively prevents pullout of the cement matrix lamellae, allowing PPAC Fold-7 to sustain loads even after complete crack propagation without catastrophic failure (Fig. 2b, i and Fig. 4j, k, L, Suppl. Figs. 21–22, Suppl. Movie 2). In contrast, when the number of rolling-folding cycles is relatively small, the solid phase thickness increases sharply (Suppl. Fig. 16), thereby weakening the mechanical interlocking effect and further confirming the positive correlation between rolling-folding cycles and PPAC performance. The synergistic effects of crack deflection and interlamellar mechanical interlocking provide PPAC with a toughening mechanism analogous to that of nacre-like materials, despite their fundamental architectural difference (in PPAC, the weak interfaces serve as the discrete phase, whereas in nacre the solid platelets represent the discrete phase).

For further supporting the thermal insulation results, we conducted a multi-physics heat transfer simulation based on the obtained PPAC architecture. In this simulation, conjugate conduction through both the solid phase (cement matrix) and weakness phase (lamellar cavities), as well as thermal radiation were simultaneously considered. The simulation results were highly consistent with the fire & thermal resistance test (Fig. 4m, Suppl. Movie 3). During model construction, a simplified 2D model was employed (Suppl. Fig. 30). The weak cavities were idealized as rectangles with an aspect ratio of 10:1 based on the observations in Fig. 4b, and the pore medium was defined as air. The pore area in the 2D model was determined according to the density of the Fold-7 specimen. A regularly staggered arrangement was then adopted to approximate the interlocking features and the dispersed weakness phase distribution. Although this simplified model was used, it retains the key structural characteristics of PPAC and incorporates appropriate parameterization. While the simulation is not intended for quantitative prediction, it still enables a directional comparison with the cast counterpart, thereby partially reflecting the thermal insulation advantage of PPAC.

The thermal insulation mechanism of PPAC closely resembles the nacre-like ceramic structures reported in the literature^17^ (Fig. 4n). In terms of heat transfer, the discrete distribution of lamellar cavities greatly restricts air convection, while the multiple lamellar cavities along the conduction path create successive low-conductivity regions that effectively hinder heat conduction. Meanwhile, the well-aligned solid–air interfaces promote multiple reflections of infrared radiation. Collectively, these effects significantly obstruct heat transfer within the lamellar cavities of PPAC, causing heat to be concentrated near the exposed surface but strongly attenuated in the interior, resulting in evident heat transfer resistance along the direction perpendicular to the lamellar arrangement.

### Performance advantages

**Fig. 5:**
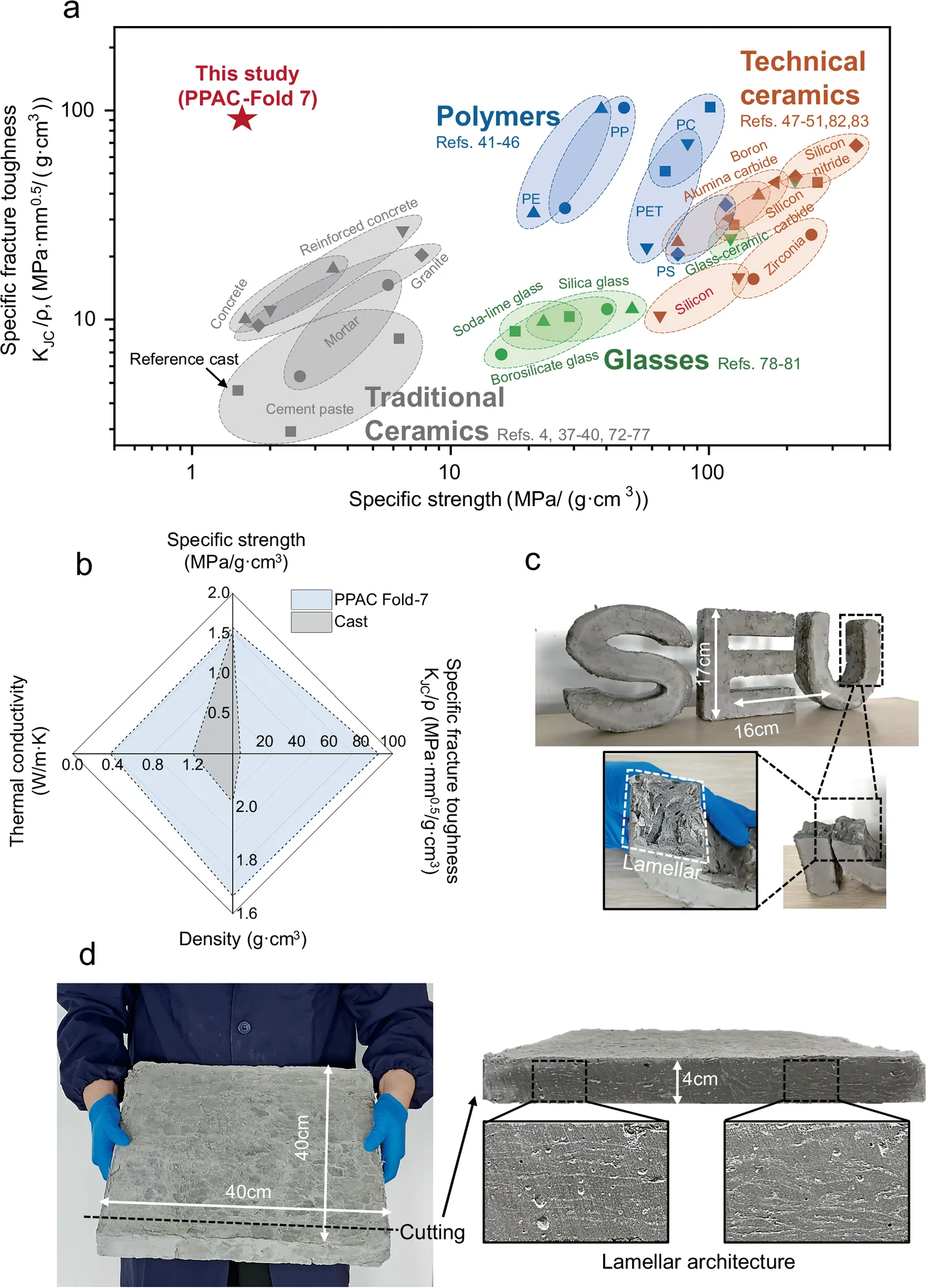
Performance advantages of PPAC and its application potential. **a** Ashby plot of specific fracture toughness versus specific strength, displaying the performance advantages of our PPAC materials compared with a wide range of engineering materials^4,37–51,72–83^. Material selection and classification were conducted with reference to Refs. ^5,22,26^ Data points represent literature-reported values. The elliptical regions are manually defined schematic ranges for illustration only and are not intended for quantitative interpretation. Source data are provided as a Source Data file. **b** Multifunctionality of PPAC Fold-7 comparing to ordinary cast cement paste. PPAC combines high toughness, thermal insulation, and lightweight properties. **c** Formability of PPAC, featuring that PPAC can be fabricated into various geometries. The Letter “SEU” is the abbreviation for Southeast University. **d** Scalability of PPAC, featuring that PPAC can be fabricated into large-scale structural component.

The Ashby plot (Fig. 5a) illustrates the performance advantages of PPAC in a plot of specific fracture toughness versus specific strength, compared with conventional cementitious materials^4,37–40,72–77^, glasses^78–81^, polymers^41–46^, and advanced technical ceramics^47–51,82,83^. It is evident that the specific fracture toughness of PPAC-Fold-7 significantly exceeds that of traditional cement paste, mortar, concrete, and fiber-reinforced concretes, reaching a value of about 91 MPa·mm^0.5^/g·cm³, comparable to polymers such as polypropylene (PP) and polycarbonate (PC) as well as technical ceramics such as silicon nitride.

Relative to ordinary cast cement paste, PPAC demonstrates evident multifunctionality (Fig. 5b). At a similar level of specific strength, PPAC exhibits a lower density (17.7% lower than that of cast cement paste), a much higher specific fracture toughness (more than 20 times that of cast cement paste), and a significantly reduced thermal conductivity (68.3% lower than that of cast cement paste). These advantages are highly desirable for resilient and energy-efficient cities in civil engineering and infrastructure applications.

Moreover, a particularly significant feature is the simplicity of the PPAC fabrication process. During fabrication, no hot-pressing, vacuum treatment, freeze–thaw cycling, or manual cutting–assembly is required. Instead, the rolling-folding process can be fully conducted at room temperature, with seven rolling-folding cycles completed within approximately 30 min, producing PPAC-Fold-7 with excellent toughness and thermal insulation performance in this study. Because the process is conducted entirely during the plastic stage of cementitious materials, the fresh PPAC upon fabricated retains adequate formability and geometric adaptability. We further demonstrate this by molding PPAC into the letters “SEU” (abbreviation of Southeast University), where the lamellar structure is well preserved after hardening (Fig. 5c, Suppl. Fig. 23). This confirms that PPAC can be easily shaped into diverse geometrical components. Moreover, large-scale PPAC components can be fabricated simply by increasing the roller size and material volume, or by joining multiple PPAC units during the plastic stage (Fig. 5c, Suppl. Fig. 24). Such formability and scalability offer strong feasibility for large-scale and practical construction, overcoming long-standing limitations in the mass fabrication and scaled applications of cementitious architected materials.

## Discussion

In this study, we developed a previously undiscovered puff pastry architected cementitious material (PPAC) featuring a layered architecture with discrete lamellar cavities, which simultaneously exhibits excellent specific fracture toughness (about 91 MPa·mm^0.5^/g·cm³) and good thermal insulation (0.38 W/m·K).

Existing architected cementitious materials typically rely on complex fabrication processes such as ice templating, hot pressing, freeze–thaw cycling, or laser cutting and assembly, which impose severe limitations on their scalability and hinder their practical application in infrastructure. In contrast, our bulk PPAC can be produced easily under ambient mild conditions through a simple rolling–folding process, offering remarkable scalability and formability. This enables the fabrication of large-scale components with diverse geometries, thereby overcoming the long-standing barriers to the infrastructure-scale applications of architected cementitious materials.

The high fracture toughness and ductile failure behavior of PPAC can enhance the structural safety of infrastructure by mitigating catastrophic collapse and reducing associated risks and losses. Meanwhile, its excellent thermal insulation performance can enable efficient thermal management, reducing energy consumption for temperature control and associated carbon emissions. We envision that PPAC could play an important role in advancing both the safety and service performance of next-generation infrastructure.

However, despite the demonstrated performance advantages and application potential of PPAC, this work represents only an initial step toward its large-scale implementation in real construction scenarios, and many questions remain to be addressed. For example, while SA-crosslinked hydrogels have been shown to effectively introduce weak interfaces in cementitious materials, their physicochemical properties and the influence of their tunability on interfacial morphology require further investigation. Moreover, other materials capable of generating similar weak interfaces may exist, offering opportunities to expand the PPAC concept and potentially introduce additional functionalities. In addition, considering the complex service conditions of infrastructure, more systematic and strategy-driven studies are needed, such as compressive performance optimization (Suppl. discussion 1), size-dependent behavior (Suppl. discussion 2), customized structural design, and long-term durability (Suppl. discussion 3).

To translate this concept from laboratory-scale demonstration to practical applications, our future work will focus on both material and structural optimization. In particular, considering the carbon footprint of cementitious systems and the widespread use of aggregates in real construction, we will explore extending the PPAC concept to mortar, concrete, and even fiber-reinforced composites.

Furthermore, one of the key advantages of PPAC is its mild processing conditions, simple fabrication procedure, and low cost (Suppl. discussion 4). The fabrication process used in this study is limited to laboratory-scale demonstrations and primarily serves as a proof of concept. From an industrial perspective, further optimization of the process is required to improve reproducibility and ensure consistent product quality. According to the fundamental principle underlying PPAC fabrication, each step in the process has strong potential for automation. The integration of robotic assistance, additive manufacturing techniques and industrial automation systems could enable the development of efficient and scalable production lines for PPAC.

Nevertheless, this study establishes a proof-of-concept for PPAC with combined high toughness and thermal insulation, providing valuable insights for the development of high-performance and multifunctional infrastructure materials. With continued advances, PPAC may enable cost-effective, energy-saving and scalable manufacturing of infrastructure systems that simultaneously offer enhanced safety and efficient thermal management, thereby improving the comfort and resilience of human living and working environments.

## Methods

### Fabrication of cementitious puff pastry architected material (PPAC)

Sodium alginate (SA) solution was prepared by dissolving the SA powder (Analytical Reagent, AR) in water, followed by continuous stirring at ambient temperature until the mixture reached homogeneous and transparent state. P·O 42.5 Portland cement, tap water and hydroxypropyl methylcellulose (HPMC) with viscosity of 75,000 Pa·s were mixed to fabricate cement paste. The water-to-cement ratio was 0.3, content of HPMC was 2 wt.% of cement. After continuous mixing for 5 min, the cement paste was removed from the mixer, wrapped with silicone release paper, and then rolled using a rolling machine to a thickness of approximately 20 mm. Then, the silicone release paper was removed, and the pre-prepared SA solution was uniformly adopted onto the surface of the cement paste, with a consumption of approximately 0.03 g/cm². After one minute, the cement paste was cut in half, the two portions stacked, wrapped in silicone release paper, and rolled again to thickness of 20 mm. The cycle of surface SA application, cutting, stacking, and rolling was subsequently repeated for the desired number of iterations. Through empirical optimization, the repetitive process was carried out up to seven times without compromising the material’s integrity, with each cycle lasting 3–5 minutes, and seven cycles could be completed within 30 min. A schematic of the PPAC fabrication process is shown in Fig. 1 and Suppl. Fig. 25.

### Mechanical testing

Three-point bending (3PB) tests were conducted according to ASTM C293M16^84^ to characterize the flexural performance of PPAC. To determine the toughness related parameters including *K*_*IC*_ and K_*JC*_, single-edge notch bending (SENB) was used according to ASTM E1820-20b^67^. The specimens used for 3PB and SENB were cut into prisms with dimensions of 40 mm (width) × 40 mm (depth) × 160 mm (length). In the SENB specimens, a 2 mm-deep initial notch was first cut using a 1 mm-thick diamond saw, and then extended by an additional 1 mm with a 300 μm-thick razor blade^5^ (Suppl. Fig. 26). During the 3PB and SENB tests, a span length of 100 mm was used, and the crosshead was displacement-controlled, with a loading rate of 1 mm/min. Digital image correlation (DIC) was additionally used to identify the nodes corresponding to the crack initiation and to observe crack propagation in situ during the SENB test. The DIC speckle pattern on the specimen surface was prepared using a transfer sticker. The speckle pattern consisted of a white background with randomly distributed black circular dots of approximately 1 mm in diameter (Suppl. Fig. 26). A schematic of the test setups for 3PB and SENB is shown in Suppl. Fig. 27. The details of the mechanical parameter calculation including MOR, Flexural toughness, *K*_*IC*_, and *K*_*JC*_ are given in the Suppl. method 1.

### Microstructure characterization

X-ray computed tomography (X-CT) scanning and three-dimensional reconstruction were conducted to characterize the lamellar architecture of PPAC. An X-CT scanner (YXLON International, Y. CT Precision) was operated at 0.24 mA and 195 kV, with a resolution of 50 μm. The reconstructed 3D images were visualized and analyzed using commercial software Avizo 3D (ThermoFisher Scientific, USA). The thickness distributions of the weakness phase and the solid phase were determined by creating five equally spaced slices perpendicular to the lamellar orientation and calculating the pixel distribution of different phases within each slice (Suppl. Fig. 28). The length distribution of the weakness phase was obtained by manually measuring its length in these slices using ImageJ software. Scanning electron microscopy (SEM) combined with energy-dispersive spectroscopy (EDS) was employed to further verify the lamellar structural features and composition of PPAC. A ZEISS Sigma 360 microscope (Carl Zeiss, Germany) was used. Prior to observation, the specimens were sputter-coated with Au to improve conductivity.

### Thermal insulation characterization

Specimens were cut into dimensions of 40 mm(length) × 40 mm(width) × 35 mm(depth) and placed on a support. For the PPAC specimens, the depth direction was oriented perpendicular to the lamellar structure. A blowtorch was positioned parallel to the specimen depth direction, and the nozzle was 5 cm away from the specimen surface. A butane flame from the blowtorch was continuously applied to the center of the specimen surface, with a core temperature of about 1300 °C. Simultaneously, an infrared camera was positioned 10 cm away from the opposite side of the specimen to record the temperature evolution on the specimen’s back surface in real time during heating. This test was conducted for 15 min on the cast specimens and for 20 min on the PPAC specimens. The schematic of the fire & thermal resistance test is shown in Fig. 3a. In addition, the thermal conductivity along the depth direction was quantitatively measured using a Hot Disk TPS 2500S (Sweden) at room temperature. During the test, two specimens of identical dimensions were used to sandwich the sensor probe, and the specimen size was consistent with that employed in the fire & thermal resistance tests. For PPAC specimens, the measured thermal conductivity corresponded to heat transfer along the direction perpendicular to the lamellar structure (Suppl. Fig. 29).

### Numerical modeling for heat transfer in PPAC architecture

Numerical simulations of heat transfer in the PPAC architecture were performed using COMSOL Multiphysics with coupled solid–fluid heat transfer and thermal radiation modules. A two-dimensional model with dimensions of 40 mm (length) × 35 mm (width) was built to represent the cross-section of PPAC. Due to the minimum scale limitations of the software, the weakness phase was simplified into rectangular air layers of 10 mm (length) × 1 mm (thickness) according to the length-to-thickness ratio. The total area of the weakness phase relative to the solid matrix was set based on the density of PPAC-Fold-7. Details of the model are provided in Suppl. Fig. 30. The thermal conductivity of the cement matrix was set to 1.2 W/m·K according to Hot Disk measurements, and its emissivity was set to 0.95 according to the literature^85–87^. A linear heat source with a heat flux of 500 W·m⁻² was applied on one side of the model to simulate flame exposure. Continuous heating for 20 min was simulated, and the results are presented. A video of the simulation is provided in Suppl. Movie 3.

### Statistical analysis

Data in the bar figures is presented as mean ± SD. For the statistical methods such as mechanical test and hot disk test, a minimum of 3 repetitions were performed for each test. The t-tests were performed to confirm the significance between the results. Confidence interval of 95% and α value of 0.05 was used. *P* < 0.05 indicates a significant difference (*) and *P* < 0.01 indicates a highly significant difference (**). The calculated *t*-value, *p*-value and significance are presented in Suppl. Tables 4–10.

## Supplementary information

Supplementary Information

Description of Additional Supplementary Files

Supplementary Movie 1

Supplementary Movie 2

Supplementary Movie 3

Transparent Peer Review file

## Source data

Source data

## Data Availability

The data supporting the findings of this study and generated in this study are available in the Supplementary Information/Source Data file. Source data are provided with this paper.
