# Supplementary material for: Tough, thermal insulating and scalable puff pastry-inspired architected cementitious material via simple rolling-folding
Source: Nat Commun. 2026 Aug 27;17:10244. doi: 10.1038/s41467-026-77120-z (PMC13616957; doi:10.1038/s41467-026-77120-z)
Supplement: Supplementary file 1 [file 41467_2026_77120_MOESM1_ESM.pdf]

Supplementary Information

Tough, thermal insulating and scalable puff pastry architected cementitious material via simple rolling-folding

Kailun Xia[1], Yuning Chen[2, 1], Xingzi Liu[1], Siyuan Qiu[1], Qian Lin[1], Yi Tang[1], Tiantian Xiang[1], Sule Chen[1], Enlai Dong[1], Zijian Jia[3], Wei Wang[1], Yu Chen[1], Yamei Zhang[1, *]

[1] *State Key Laboratory of Engineering Materials for Major Infrastructure, School of Materials Science and Engineering, Southeast University, Nanjing 211189, China.*

[2] *Hefei National Research Center for Physical Sciences at the Microscale, University of Science and Technology of China, Hefei 230026, China.*

[3] *College of Civil Engineering, Nanjing Tech University, Nanjing 211816, China.*

[*]Corresponding author: Yamei Zhang (ymzhang@seu.edu.cn)

This file includes:

Supplementary Figures 1 to 33

Supplementary Tables 1 to 14

Supplementary Equations 1 to 16

Supplementary Discussions 1 to 4

Supplementary Method 1

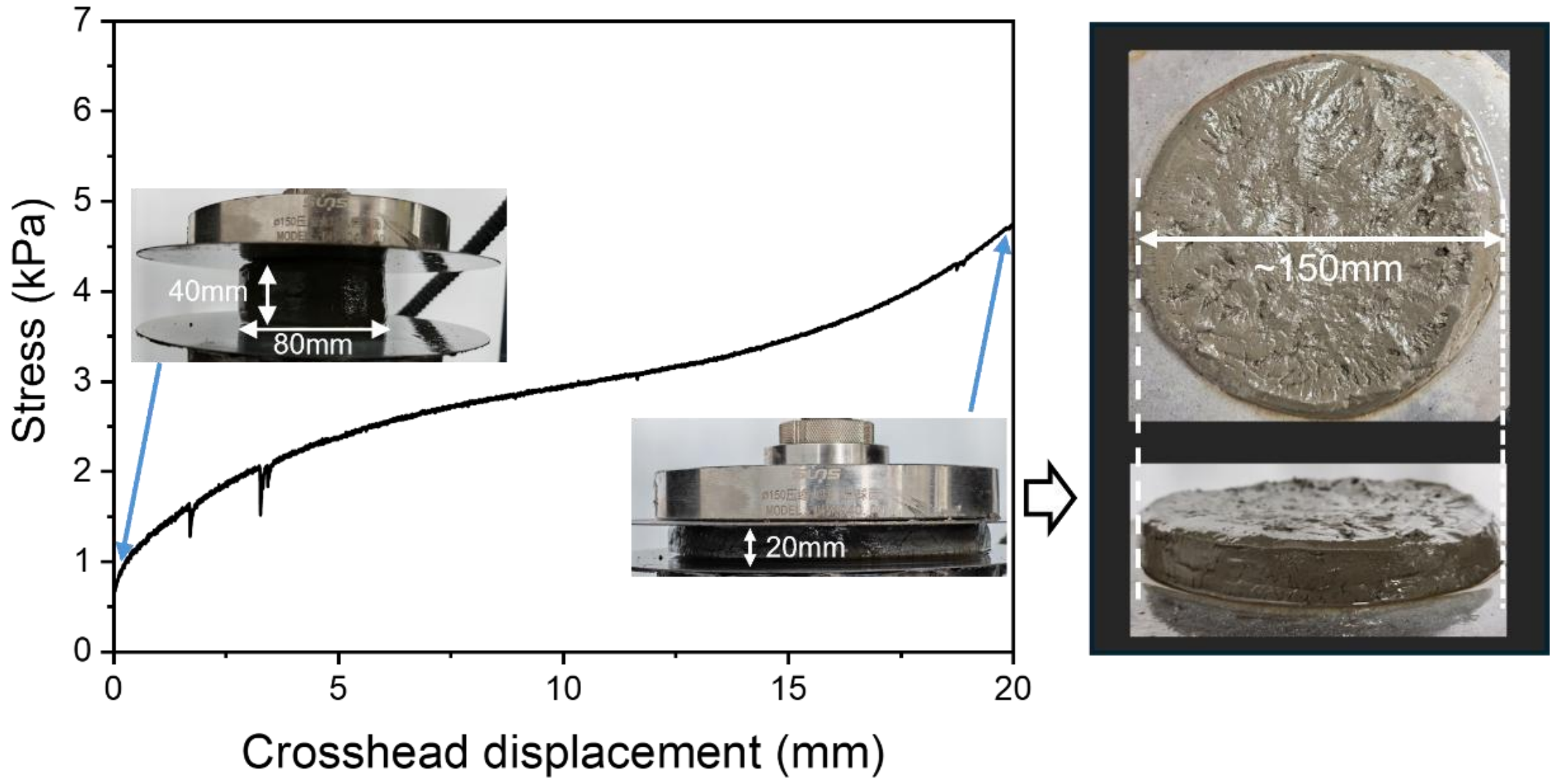

Supplementary Fig. 1. Compressive deformation and stress-strain curves of the prepared fresh cement paste. The water-to-cement ratio is 0.3. Hydroxypropyl methylcellulose (HPMC) powder with viscosity of 75000 Pa·s was added at 2wt.% of cement. The test was conducted after mixing the cement paste for 5 min. The cement paste was cast into a cylinder with a diameter of 80 mm and a height of 40 mm, and then compressed at a crosshead speed of 0.5 mm/s until the height reached 20 mm. The fresh cement paste exhibited dough-like compressibility and did not fracture during compression. Under compression, the cement paste expanded to a diameter of 150 mm, corresponding to a uniaxial strain of 87.5%. These results demonstrate that the prepared cement paste possesses good roll-forming capability.

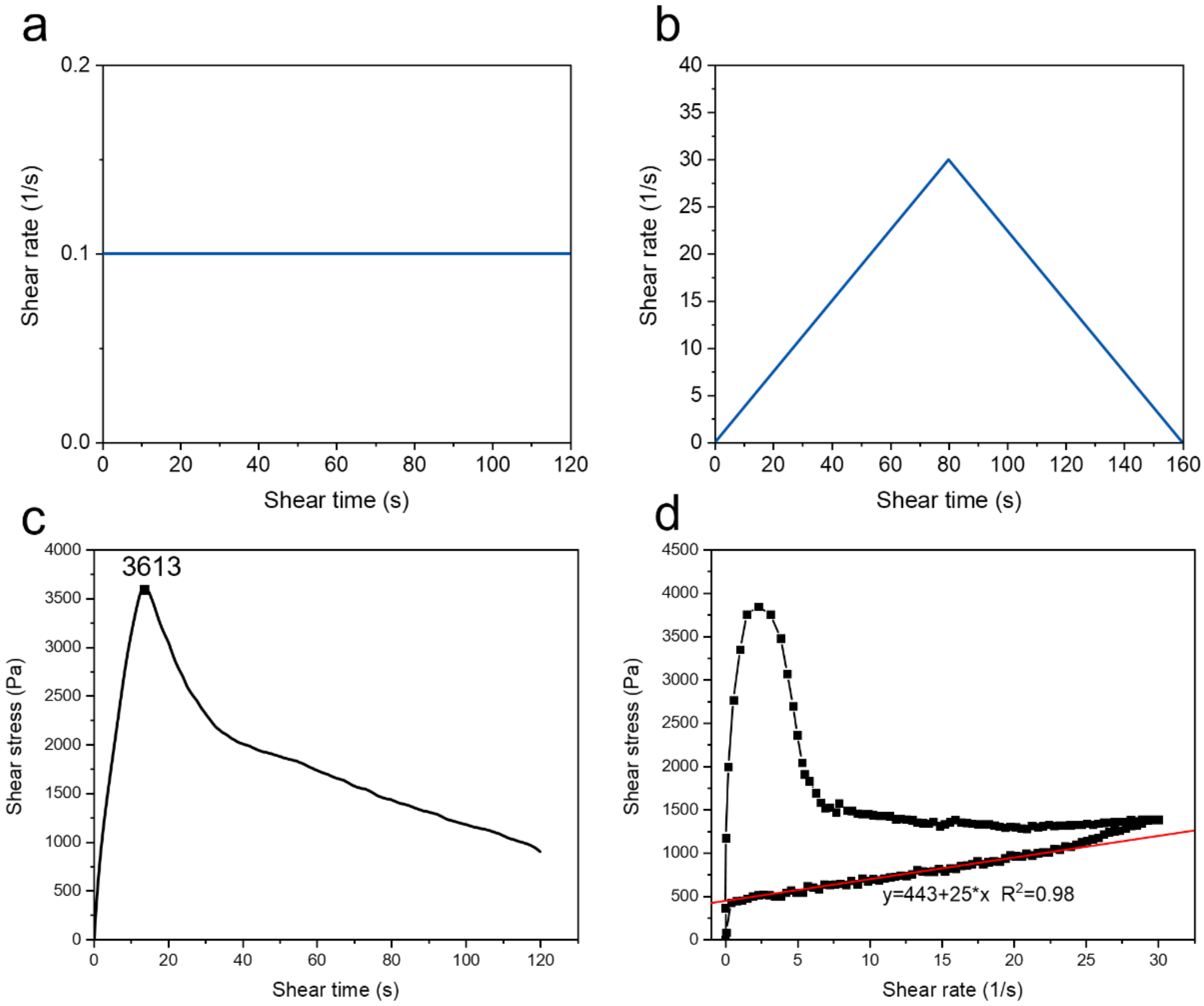

Supplementary Fig. 2. Rheological properties of the prepared fresh cement paste. These results were tested by a rotational rheometer RST-SST (Brookfield, USA) with a 20 mm×10 mm vane spindle. **a** Test protocol for static yield stress. The shear rate was kept constant at 0.1/s for 120 s. **b** Test protocol for thixotropic ring. the shear rate increased linearly from 0 to 30 s^{-1} over 80 s, then decreased linearly back to 0 over the subsequent 80 s [1]. **c** Static yield stress of the cement paste used for puff pastry cementitious architected material (PPAC) preparation was approximately 3600 Pa. **d** Plastic viscosity of the cement paste used for PPAC preparation was approximately 25 Pa·s. Dynamic yield shear stress of the cement paste used for PPAC preparation was approximately 440 Pa. The decreasing branch of the shear stress curve was fitted using the Bingham model [2].

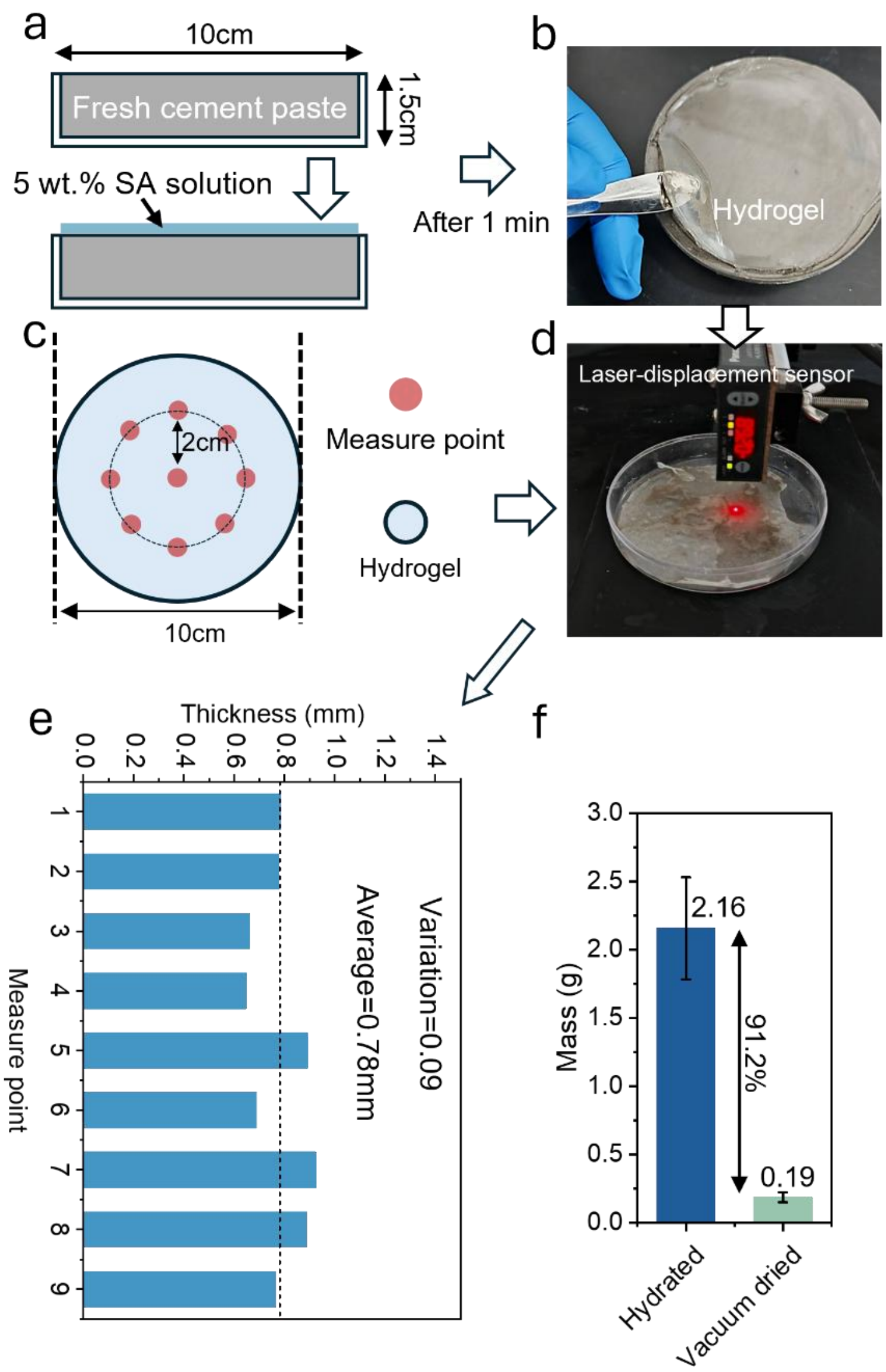

Supplementary Fig. 3. In situ generated hydrogel on fresh cement paste surface via sodium alginate crosslinking. a The fresh cement paste was cast into a circular Petri dish with a diameter of 10 cm and a height of 1.5 cm. Then, approximately 2.5 g of a 5 wt% SA solution was evenly applied to the surface of the cement paste. **b** After 1 minute, the SA solution had formed a hydrogel. **c-d** The thickness of the hydrogel was measured using a laser displacement sensor. **e** The results showed that the hydrogel had an average thickness of 0.78 mm. **f** Comparison on the mass of the generated hydrogel at the initial state (hydrated) and after vacuum dried. Data are presented as mean ± SD from n=9 measure points. The result indicates that the water content of the hydrogel is about 91.2%.

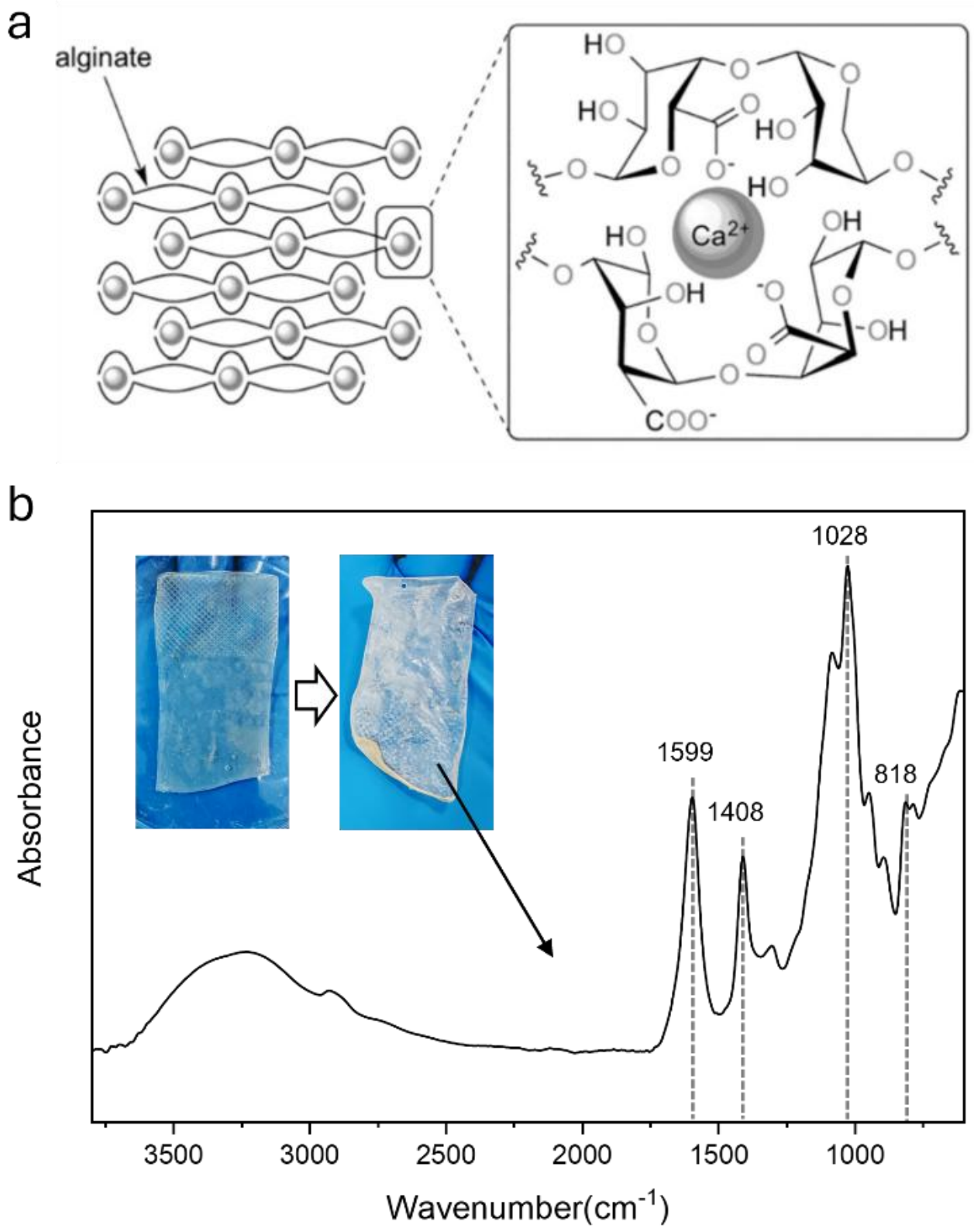

Supplementary Fig. 4. Chemical structure of the SA crosslinked product. a The “egg-box” structure formed by SA molecules with calcium ions[3]. **b** After drying, the hydrogel formed a film, and its Fourier Transform Infrared Spectrometer (FTIR) spectrum exhibited characteristic peaks fully consistent with calcium alginate[4]. The hydrogel presented here corresponds to the specimen after testing shown in Supplementary Fig. 7. The FTIR test was performed by a ThermoFisher Scientific Nicolet iS20 (USA).

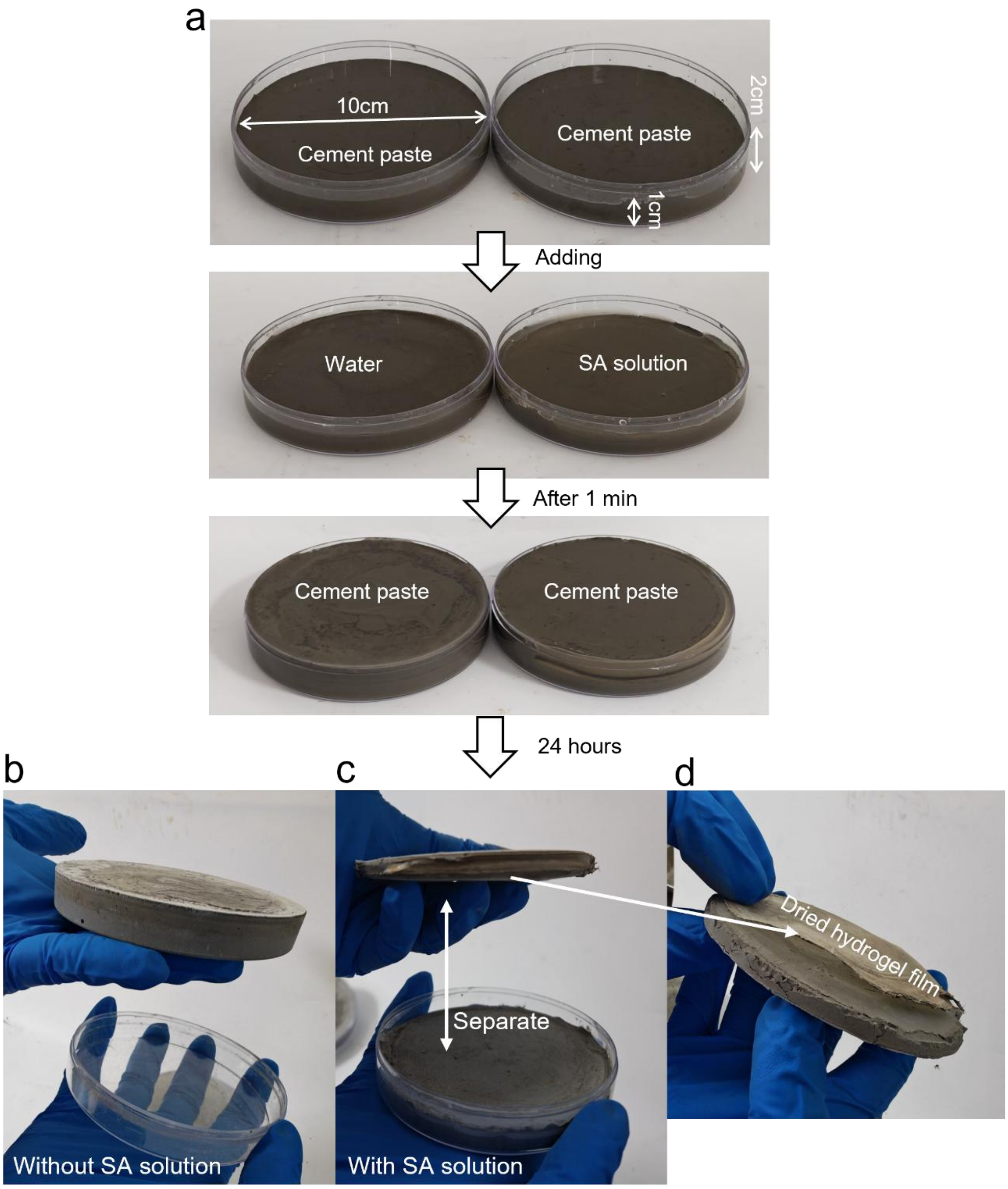

Supplementary Fig. 5. Barrier capability of the SA-crosslinked hydrogel between cement layers. a Two Petri dishes (10 cm in diameter, 2 cm in depth) were filled with 1 cm-thick cement paste. Equal masses of 5 wt% SA solution and water were then added onto the surfaces of the cement pastes, respectively. After 1 minute, another 1 cm-thick layer of cement paste was poured on each top. **b** After 24 hours, the two cement paste layers in the dish with added water were fully bonded together. **c** After 24 hours, the two cement paste layers in the dish with added SA solution were separated. **d** A distinct thin film formed after drying was observed on the bottom surface of the separated upper cement layer.

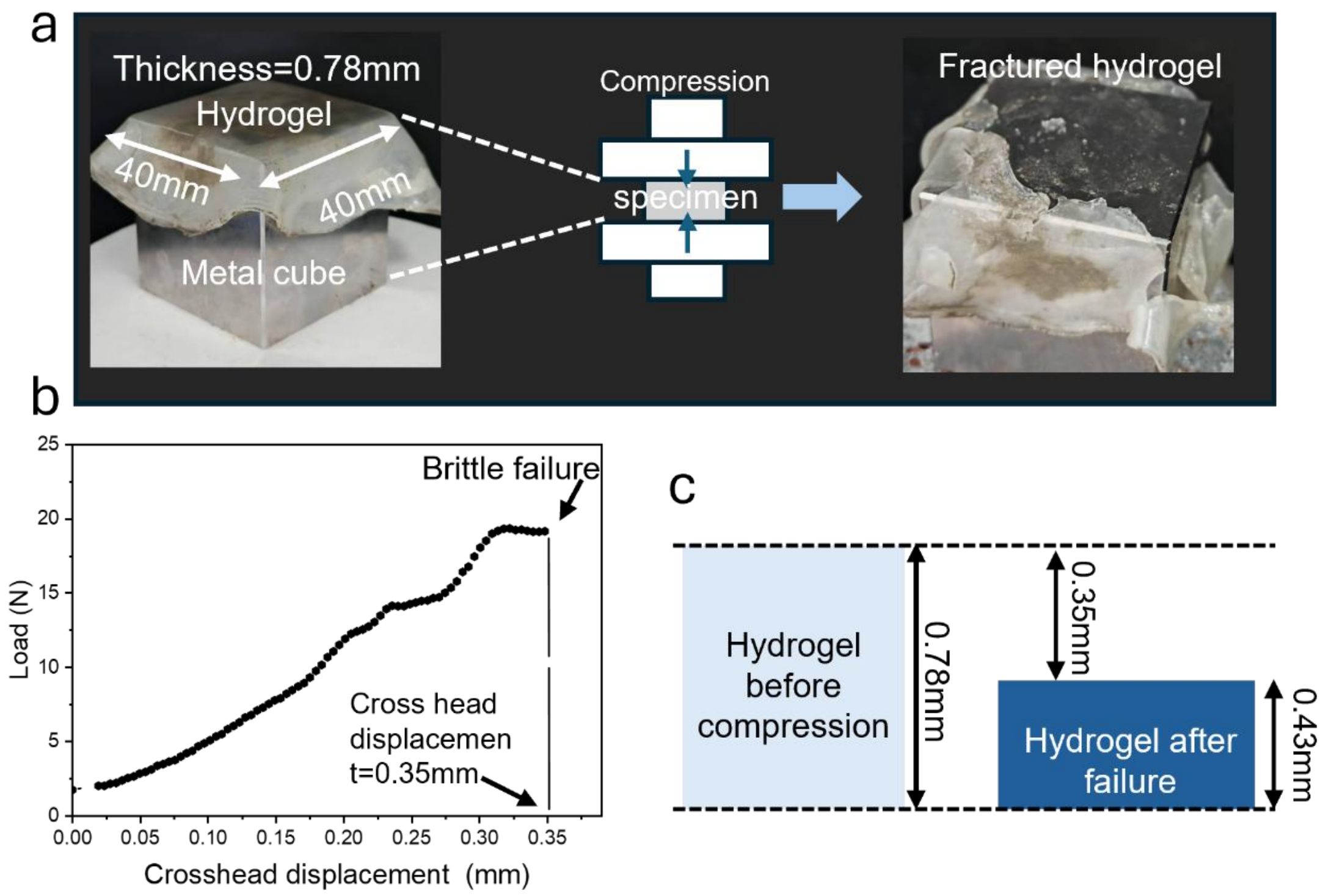

Supplementary Fig. 6. Behavior of the hydrogel under compression. a The hydrogel obtained in supplementary Fig. 3 was placed on a metal cube with dimensions of 40 mm × 40 mm × 40 mm and then compressed until fracture. **b** The load − displacement curve indicated that the hydrogel exhibited brittle failure, with an ultimate load of only ~20 N and a corresponding displacement of ~0.35 mm. **c** After compression failure, the hydrogel thickness was approximately 0.4 mm, which determines the cavity thickness inside the puff pastry architected cementitious material (PPAC) after rolling–folding.

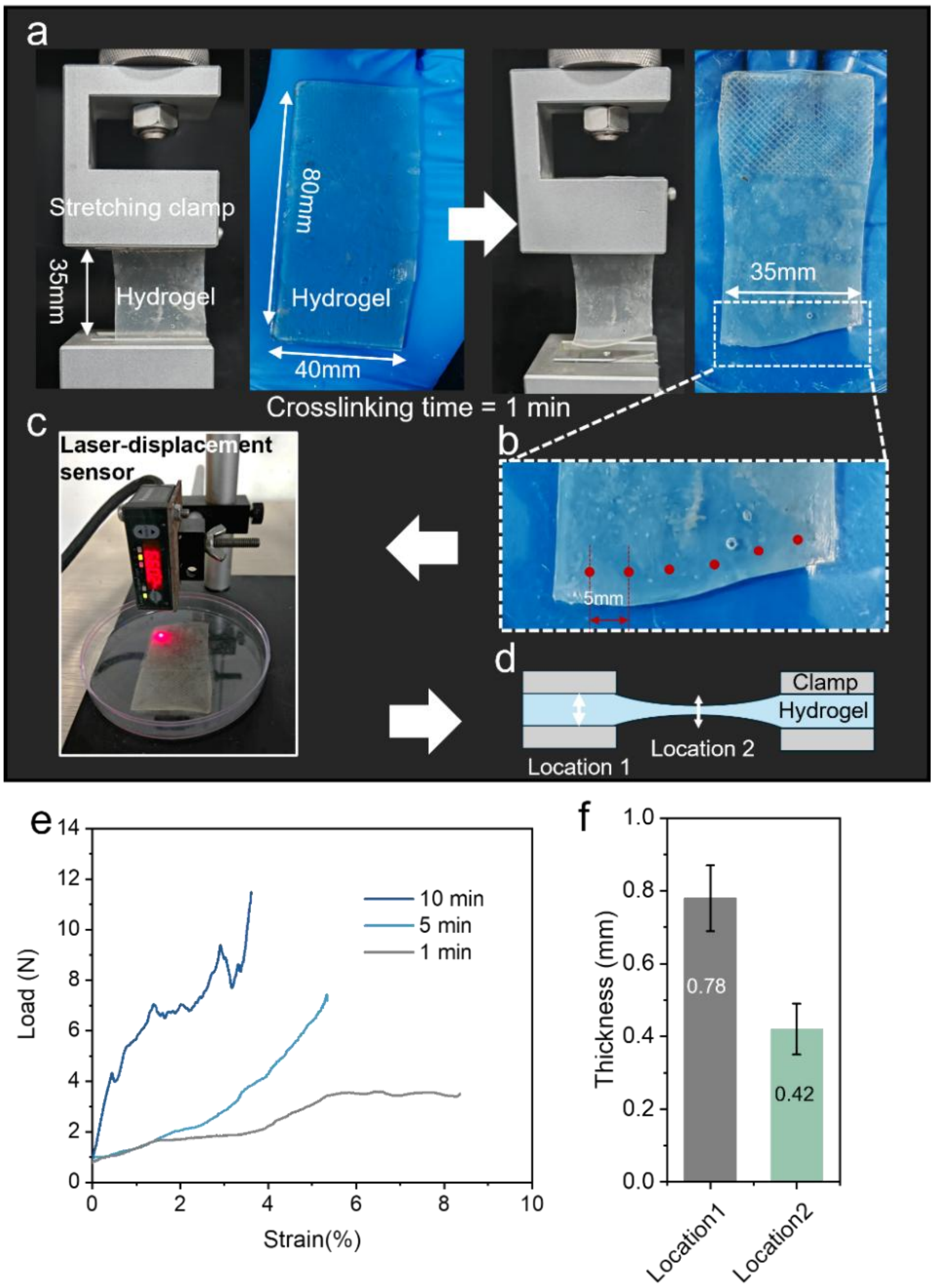

Supplementary Fig. 7. Behavior of the hydrogel under tension. a The hydrogel was cut into a rectangular strip measuring 80 mm × 40 mm. Both ends were clamped with a 35 mm gauge length in the middle, and the strip was stretched at a rate of 1 mm/min until fracture. **b-d** The hydrogel thickness at the fracture site was measured using a laser displacement sensor. **e** Tensile load – strain curves of hydrogels at different crosslinking time. The crosslinking time was determined by the interval between applying the SA solution to the cement paste surface and peeling off the hydrogel, as displaced in supplementary Fig. 3. The results show that the hydrogel also exhibited brittle fracture under tensile loading. Prolonged crosslinking time increased the hydrogel's tensile strength but also enhanced its brittleness. Under a 1 min crosslinking condition, the hydrogel's critical tensile load was less than 4 N, with a strain of under 10%. However, during the rolling process, the cement paste can experience strains up to 87.5% (see Supplementary Fig. 1). The low tensile strength and small critical tensile strain also contributed to the hydrogel's fracture behavior during rolling-folding. **f** The thickness of the hydrogel at the fracture site after 1 minute of crosslinking was approximately 0.42 mm, similar to the thickness of the hydrogel after compression fracture shown in Supplementary Fig. 6, which also determines the cavity thickness inside the puff pastry architected cementitious material (PPAC). Data are presented as mean ± SD from n=3 independent specimens.

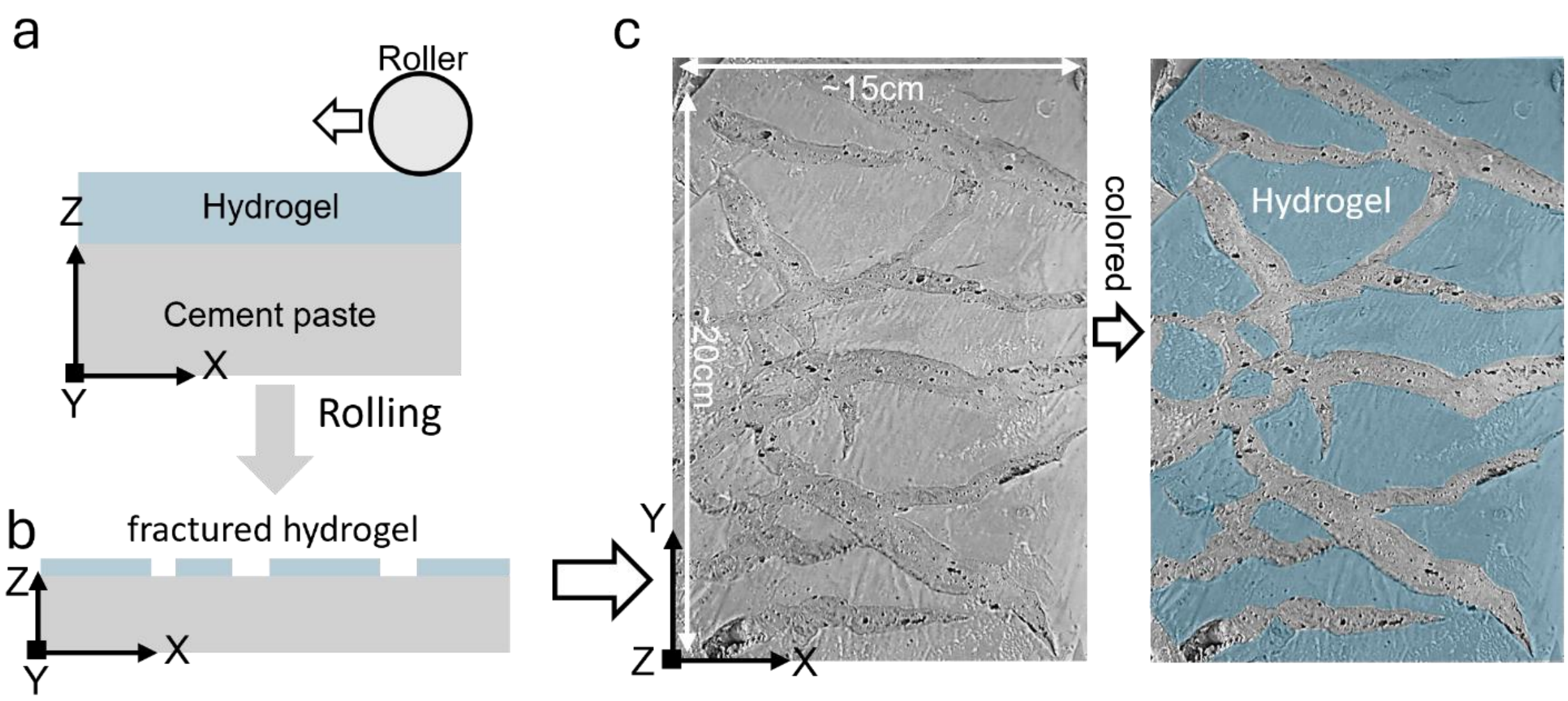

Supplementary Fig. 8. Hydrogel rupture during rolling process. a-b Schematic diagram of the hydrogel rupture during rolling. **c** Photo of ruptured hydrogel on cement paste surface after rolling procedure.

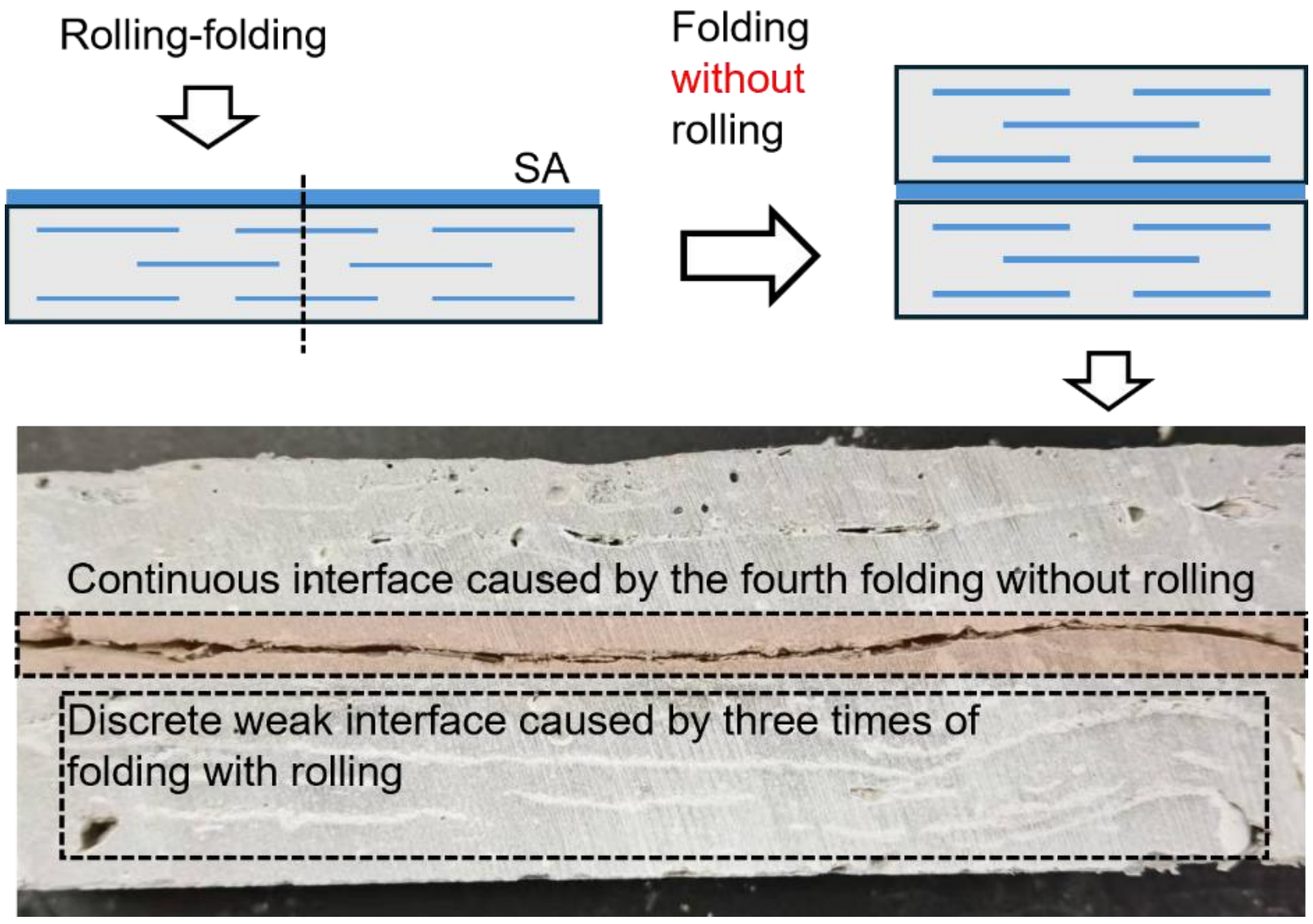

Supplementary Fig. 9. The importance of hydrogel fracture during the rolling process in maintaining the structural integrity of the material. The figure shows a specimen subjected to four folding cycles. The first three cycles involved complete rolling-folding operations, while the fourth included only folding without rolling. It can be observed that the first three rolling steps caused discrete weak interfaces, and interlocking sections still existed between the cement lamellae, maintaining the material’s integrity. In contrast, the absence of rolling in the final cycle led to the formation of a continuous weak interface through the middle of the material, which divided it into two parts and compromised its overall integrity.

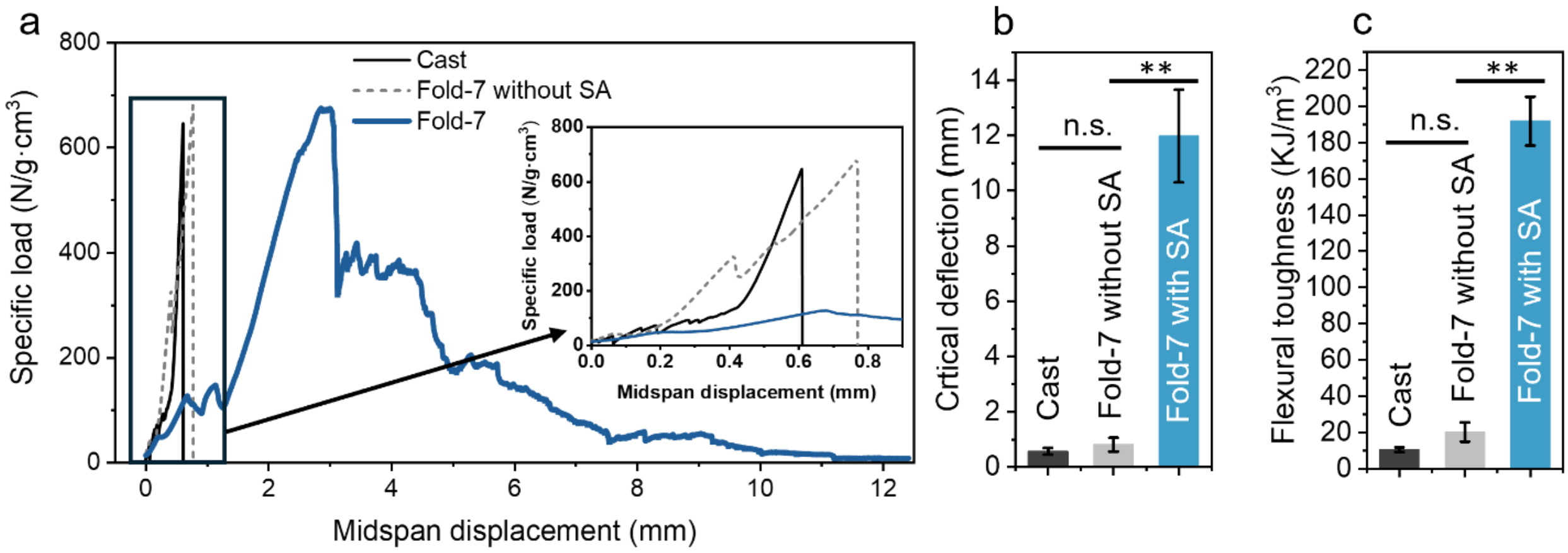

Supplementary Fig. 10. Mechanical properties of PPAC without incorporation of SA. The preparation procedure of Fold-7 without SA is essentially identical to that of the PPAC Fold-7 described in the main text, except that the step of applying the SA solution prior to each rolling cycle is omitted. **a** 3-point bending (3PB) test curves. **b-c** Comparison on critical deflection and flexural toughness between Cast, Fold-7 without SA and Fold-7 with SA. Data are presented as mean ± SD from n=3 independent specimens. * depicts $p < 0.05$ and ** depicts $p < 0.01$, which indicate statistically significant differences. *P* value is obtained from T-test. These results show no statistically significant difference between the cast and Fold-7 without SA in both critical deflection and flexural toughness. Meanwhile, critical deflection and flexural toughness of Fold-7 without SA are significantly lower than Fold-7 with SA. The significance analysis results are listed in **Supplementary Table 1.**

Supplementary Table 1. Data set and t-test significance analysis results of the mechanical properties in Supplementary Fig. 10.

Sub Table a. Data set (mean±SD).

Properties	Cast	Fold-7 without SA	Fold-7	Size (n)
Critical deflection	0.57±0.12	0.81±0.25	11.97±1.68	3
Flexural toughness	10.44±1.28	20.16±5.23	191.84±13.52	3

Sub Table b. Significance analysis on critical deflection.

Comparison	Mean difference	t-value	df	p-value	Significance
Fold-7 without SA vs Cast	0.24	1.50	3	0.23	n.s.
Fold-7 vs Fold-7 without SA	11.16	11.32	2	0.007	**

Sub Table c. Significance analysis on Flexural toughness.

Properties	Mean difference	T-value	df	p-value	Significance
Fold-7 without SA vs Cast	9.72	3.13	2	0.089	n.s.
Fold-7 vs Fold-7 without SA	171.68	19.20	2	0.003	**

* (p<0.05) indicates a significant difference; ** (p<0.01) indicates a highly significant difference; n.s. means no significant difference.

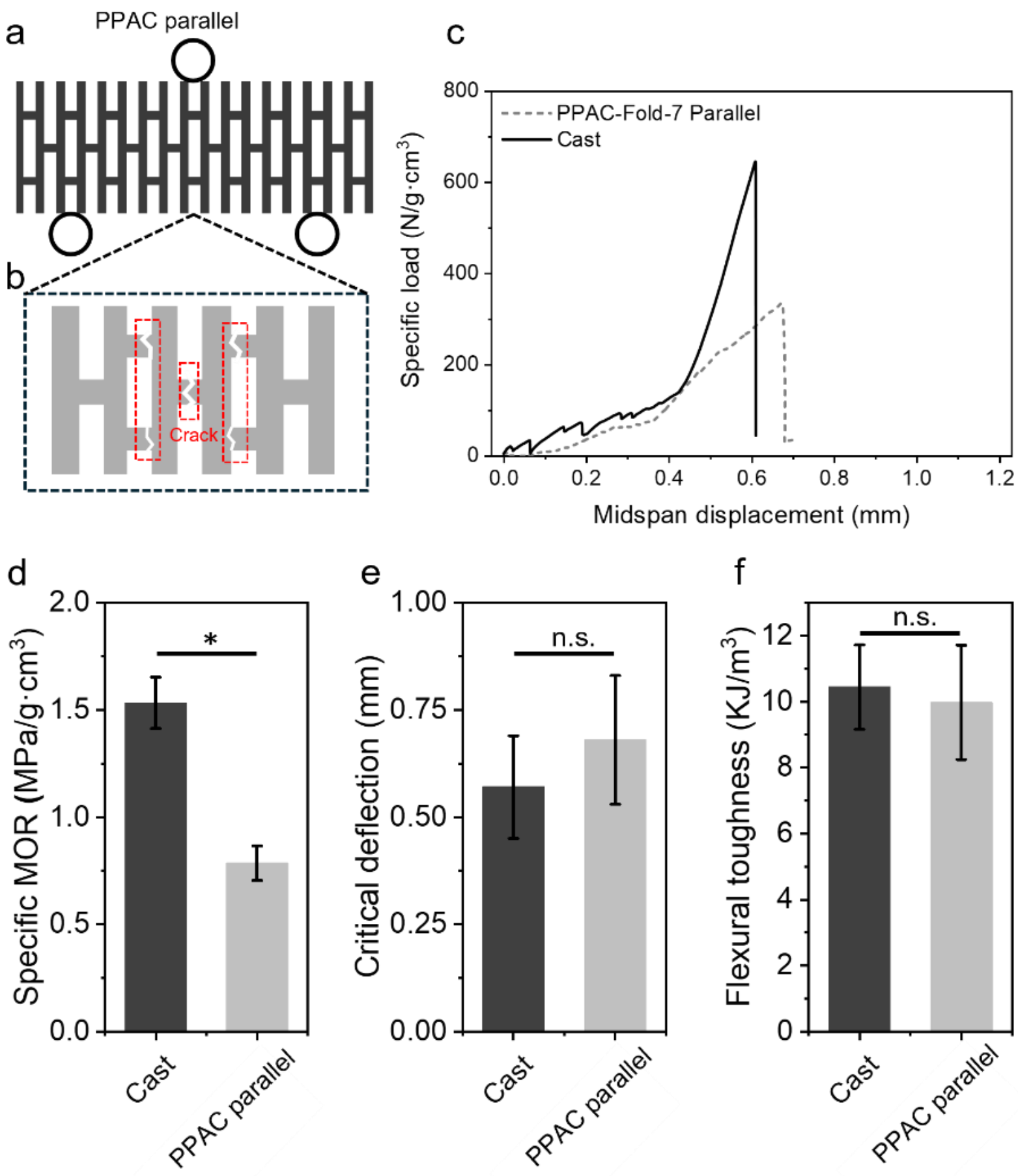

Supplementary Fig. 11. Mechanical properties of PPAC-Fold-7 when bending load is applied parallel to the weak interfaces. a Schematic diagram of the loading direction. **b** Schematic diagram of the potential fracture behavior at the material architectural scale. **c** 3-point bending (3PB) test curves. **d-f** Comparison on specific MOR, critical deflection and flexural toughness between cast and the PPAC parallel, respectively. Data are presented as mean ± SD from n=3 independent specimens. * depicts $p < 0.05$ and ** depicts $p < 0.01$, which indicate statistically significant differences. P value is obtained from T-test. The results indicate that PPAC does not exhibit a toughness enhancement when loaded parallel to the interfaces. Moreover, as the SA-induced interfaces act as preferential weakness for cracking, the MOR of PPAC can be significantly lower than that of the cast counterpart. The statistical significance analysis is listed in **Supplementary Table 2.**

Supplementary Table 2. Data set and t-test significance analysis results of the mechanical properties in Supplementary Fig. 11

Sub Table a. Data set (mean±SD)

Properties	Cast	PPAC perpendicular	Size (n)
Critical deflection	0.57±0.12	0.68±0.13	3
Specific MOR	1.53±0.12	0.79±0.08	3
Flexural toughness	10.44±1.28	9.98±1.73	3

Sub Table b. Significance analysis (Cast vs PPAC perpendicular)

Properties	Mean difference	T-value	df	p-value	Significance

Critical deflection	0.11	0.99	3	0.39	n.s.
Specific MOR	0.74	8.88	3	0.003	**
Flexural toughness	0.46	0.37	3	0.74	n.s.

* (p<0.05) indicates a significant difference; ** (p<0.01) indicates a highly significant difference; n.s. means no significant difference.

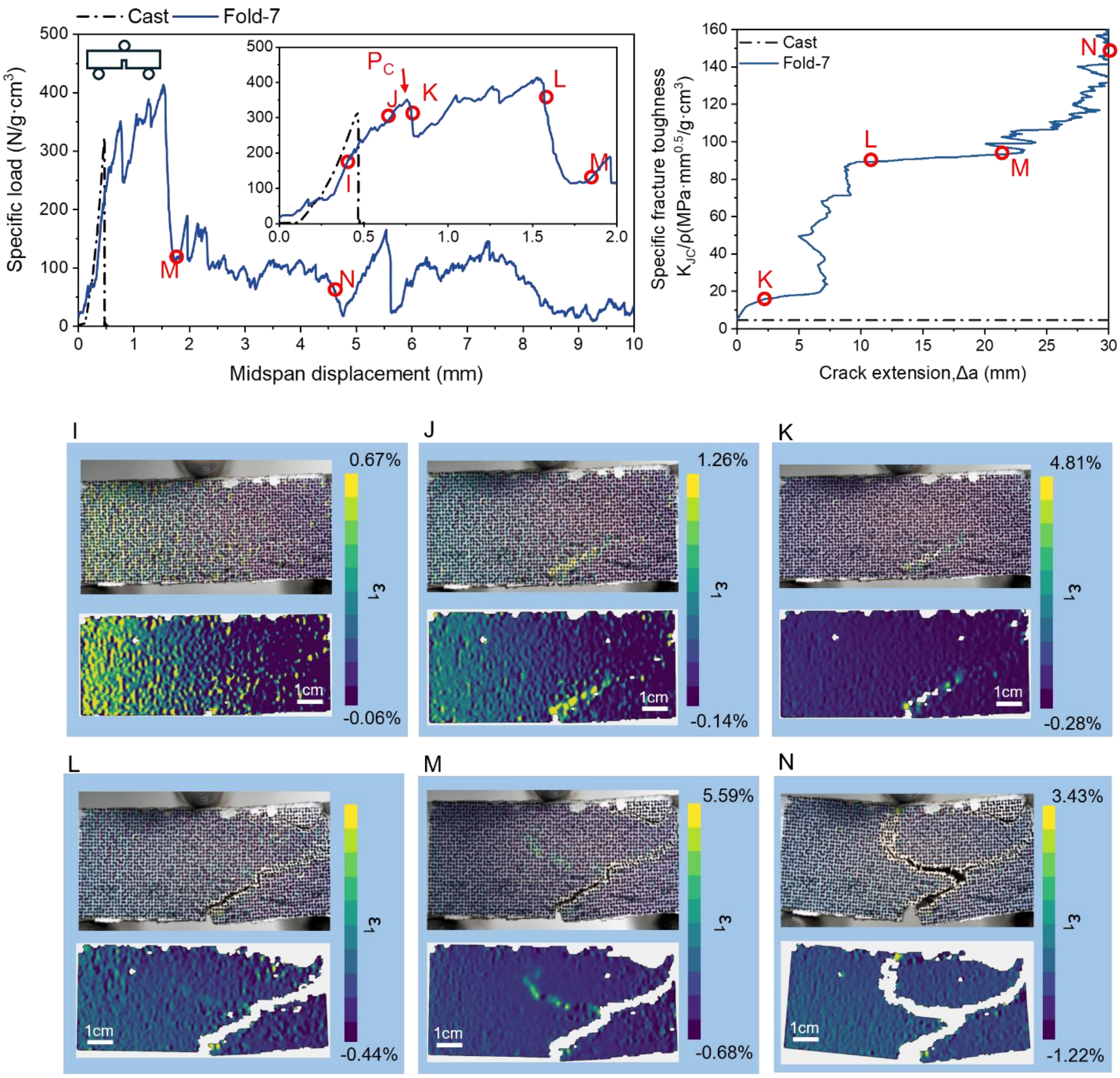

Supplementary Fig. 12. Fracture behavior of puff pastry architected cementitious material (PPAC) Fold-7 during SENB test. **I** At the initial loading stage, no significant strain occurs at the notch, and the strain distribution within the specimen is relatively uniform. **J** Noticeable strain occurs at the notch but no crack initiation is observed, representing the critical state before cracking. **K** Crack has formed at the notch. The load corresponding to the peak between J and K can be identified as P_C, the load at crack initiation. The crack shows a pronounced deflection trend. **L** The first crack has almost fully propagated. The crack propagation between K and L corresponds to the first significant rising segment of the R-curve, indicating that crack growth is significantly resisted. **M** Critical state for the initiation of the second crack. The crack again exhibits deflection. **N** The second crack has fully propagated. Crack propagation between M and N corresponds to the second significant rising segment of the R-curve, indicating that crack growth continues to be substantially resisted.

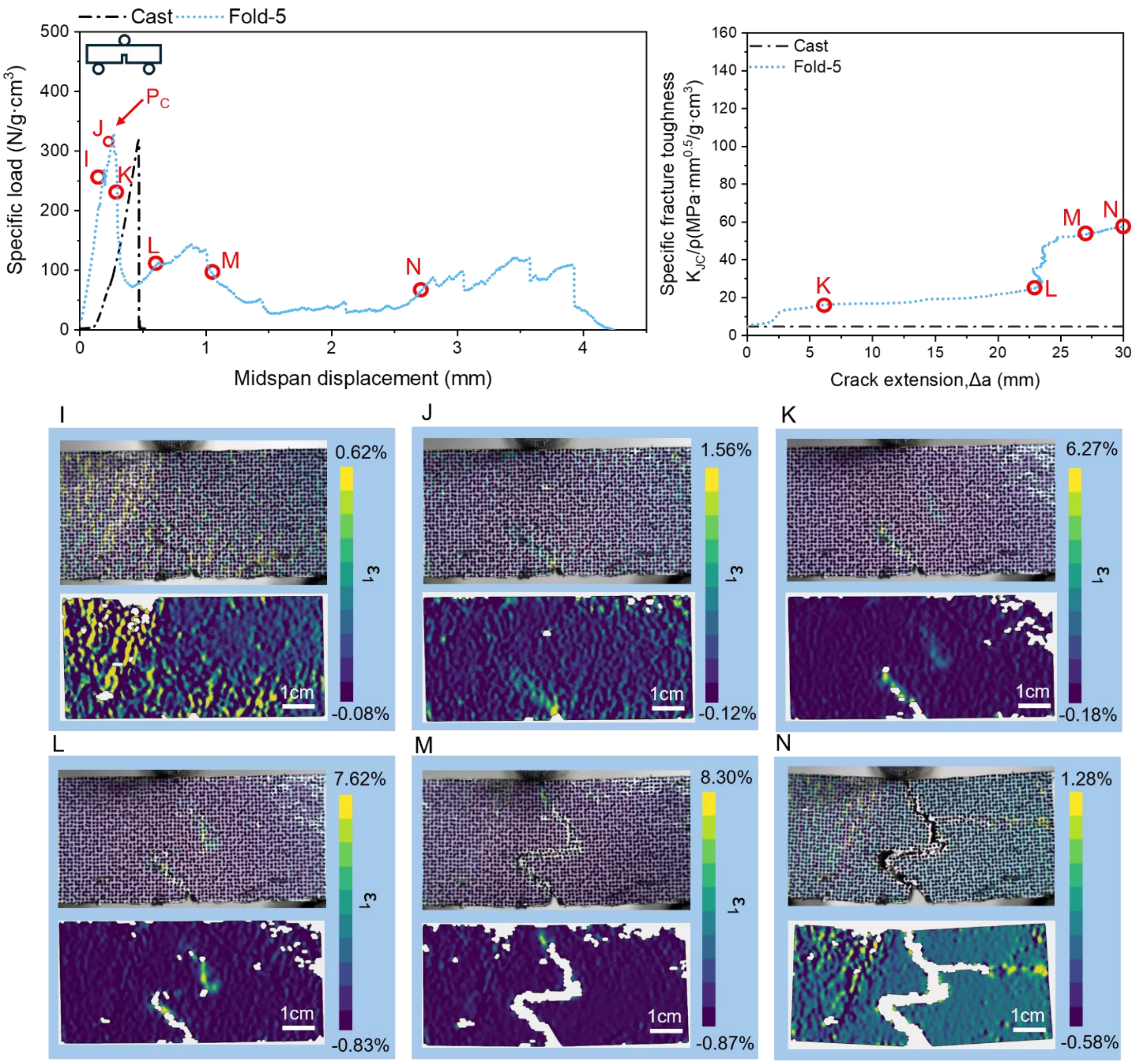

Supplementary Fig. 13. Fracture behavior of puff pastry architected cementitious material (PPAC) Fold-5 during SENB test. I At the initial loading stage, no significant strain occurs at the notch, and the strain distribution within the specimen is relatively uniform. **J** Noticeable strain occurs at the notch but no crack initiation is observed, representing the critical state before cracking. **K** Crack has formed at the notch. The load corresponding to the peak between J and K can be identified as P_C, the load at crack initiation. The crack exhibits a deflection trend, but to a lesser extent than in Fold-7 as presented in supplementary Fig. 12. **L** The crack begins to propagate along the deflected path. Between K and L, the R-curve shows a relatively weak rising trend. **M** The crack is nearly fully propagated. Between L and M, the R-curve exhibits a visible rising trend, but the magnitude is still smaller than that of Fold-7. **N** The crack has fully traversed the specimen.

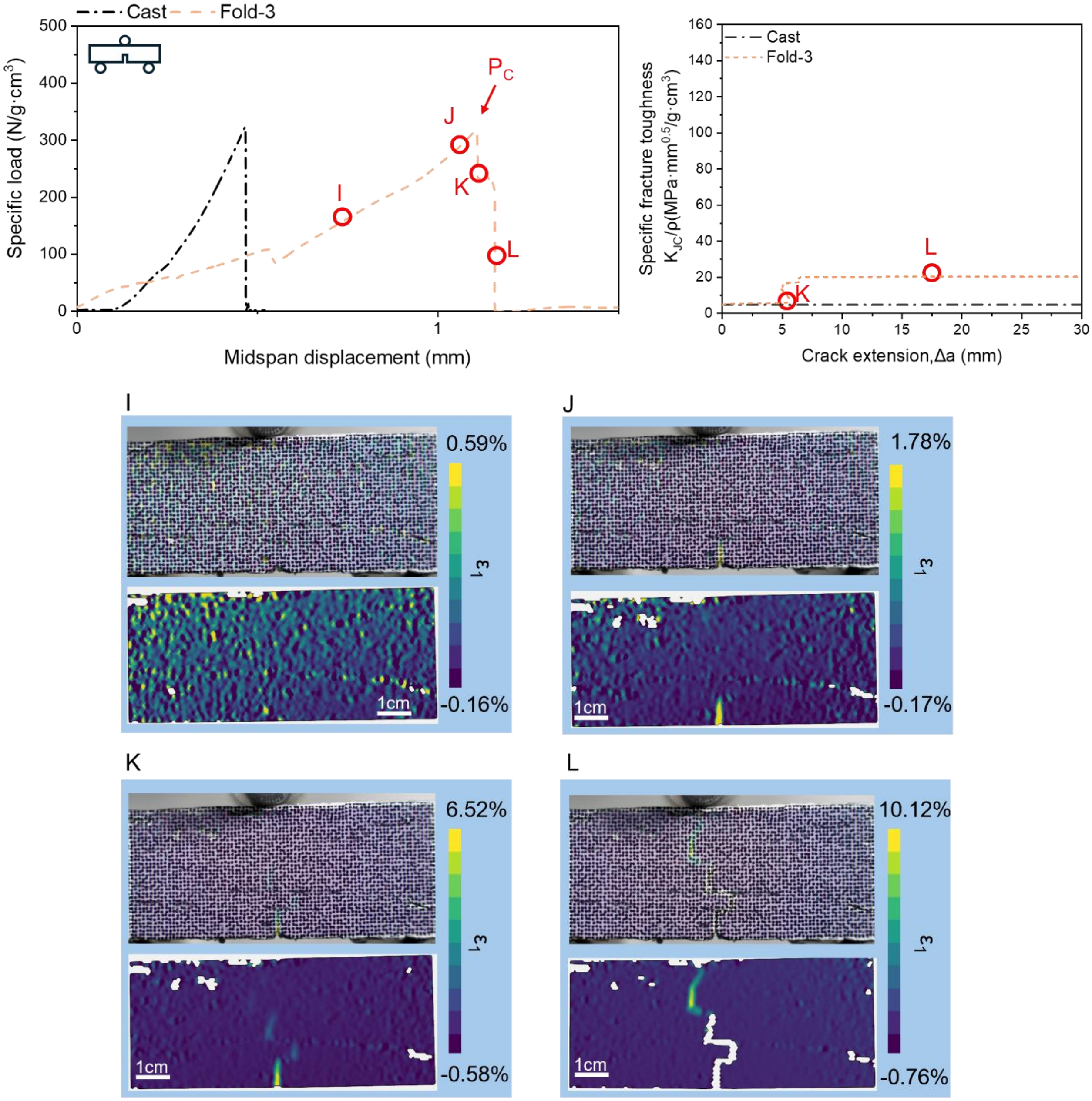

Supplementary Fig. 14. Fracture behavior of puff pastry architected cementitious material (PPAC) Fold-3 during SENB test. I At the initial loading stage, no significant strain occurs at the notch, and the strain distribution within the specimen is relatively uniform. **J** Noticeable strain occurs at the notch but no crack initiation is observed, representing the critical state before cracking. **K** Crack has formed at the notch. The load corresponding to the peak between J and K can be identified as P_C, the load at crack initiation. **L** The crack shows a certain degree of deflection but propagates almost instantaneously. Between K and L, the R-curve exhibits only a slight rising stage, indicating a very low level of resistance to crack propagation.

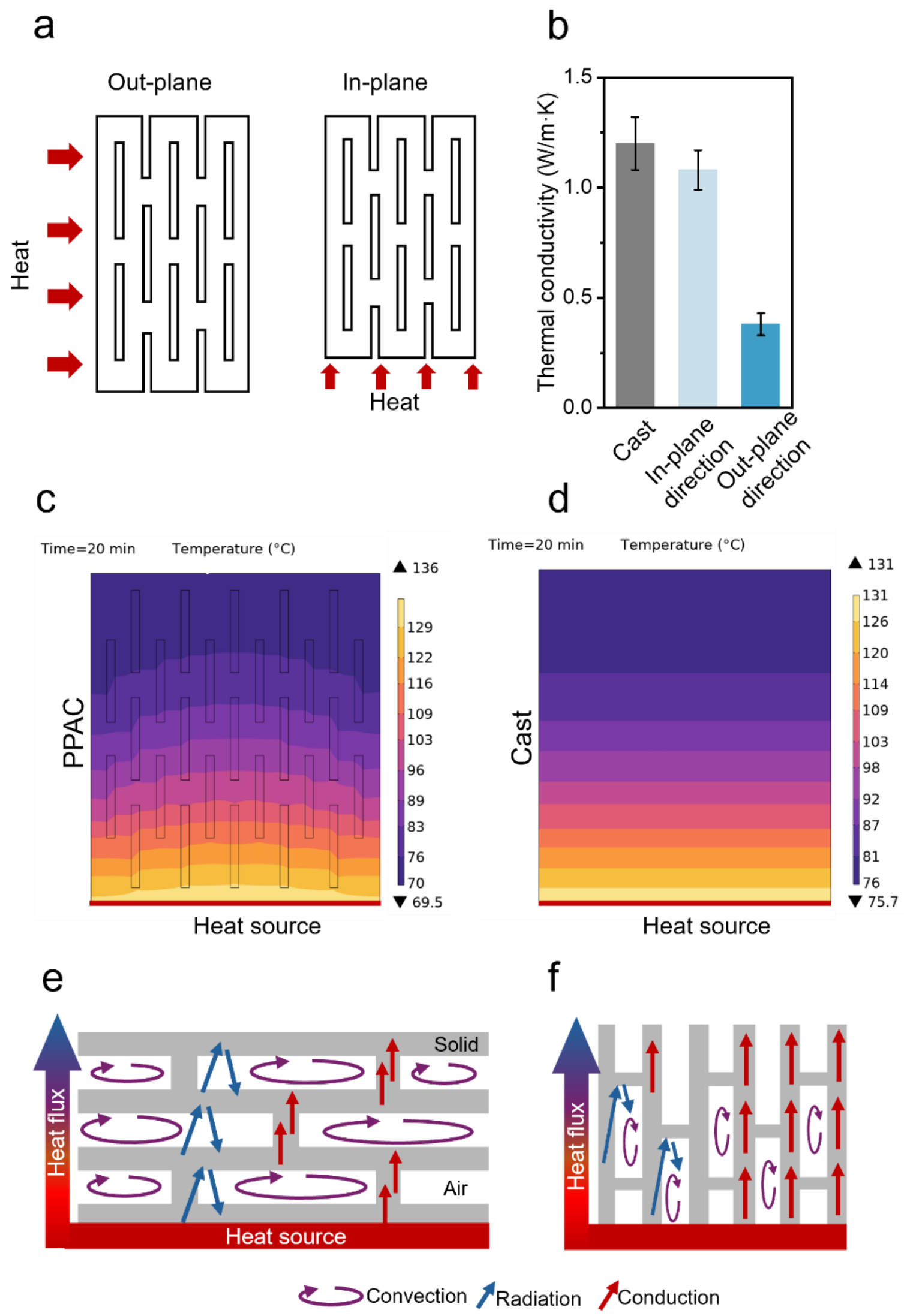

Supplementary Fig. 15 Anisotropic thermal isolation performance of puff pastry architected cementitious material (PPAC) fold-7. a Hot disk testing direction. The out-plane direction indicates heat flow perpendicular to the lamellar structure. **b** Thermal conductivity from the cast specimen and different testing directions of PPAC fold-7. Data are presented as mean ± SD from n=3 independent specimens. * depicts $p < 0.05$ and ** depicts $p < 0.01$, which indicate statistically significant differences. *P* value is obtained from T-test. The results suggest that the thermal isolation performance in the out-plane direction is remarkably better than that in the in-plane direction. **c-d** Heat transfer simulation results. In the in-plane direction, the insulating effect of the sheet-like cavity on heat transfer is significantly weakened. As a result, under the same heating duration, the backside temperature of PPAC differs from that of the cast specimen by only 6.2 °C, indicating comparable heat conduction efficiency. This observation is consistent with the thermal conductivity results. **e-f** Schematic illustrations of the thermal insulation mechanism in the out-plane and in-plane direction, respectively. The thermal insulation mechanism in the out-plane direction has been discussed in the main text. In contrast, in the in-plane direction, the continuous long-range cement lamellae provide effective pathways for enhanced heat conduction, while the reduced number of cement paste–air interfaces weaken the reflection of thermal radiation[5].

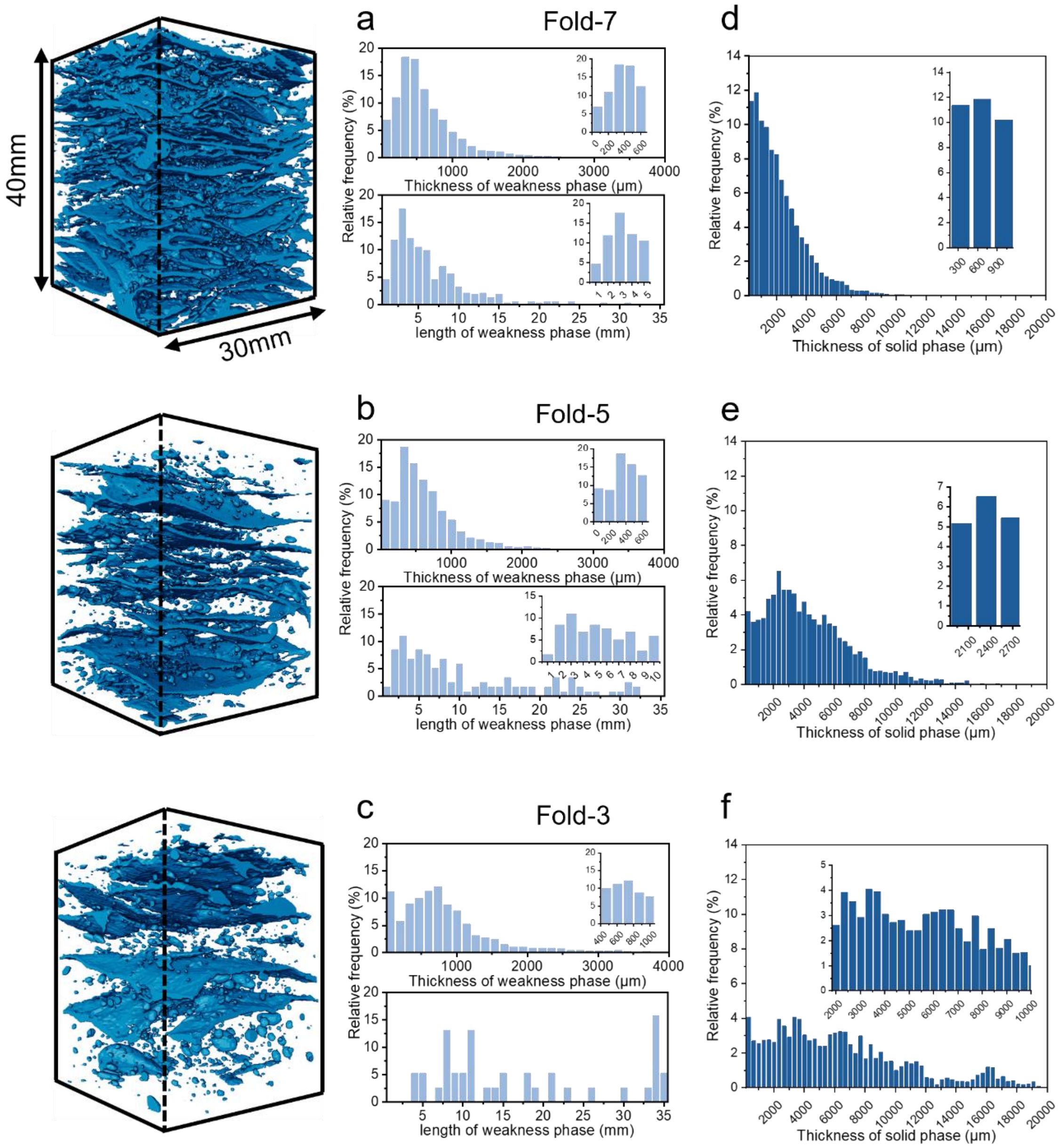

Supplementary Fig. 16. Architectural characteristics of puff pastry architected cementitious material (PPAC) materials with different times of rolling-folding cycles. a-c In Fold-7, Fold-5, and Fold-3, the weak phase thickness is predominantly distributed within several hundred μm, primarily determined by the fracture behavior of the hydrogel during rolling. Additionally, more rolling-folding cycles lead to a narrower distribution of weakness phase lengths, indicating that repeated rolling promotes more complete hydrogel rupture, forming a greater number of discrete small hydrogel lamellae. **d-f** For the solid phase thickness, increasing the number of rolling-folding cycles results in a more concentrated distribution within a smaller thickness range, with the most probable value reaching 600 μm in Fold-7.

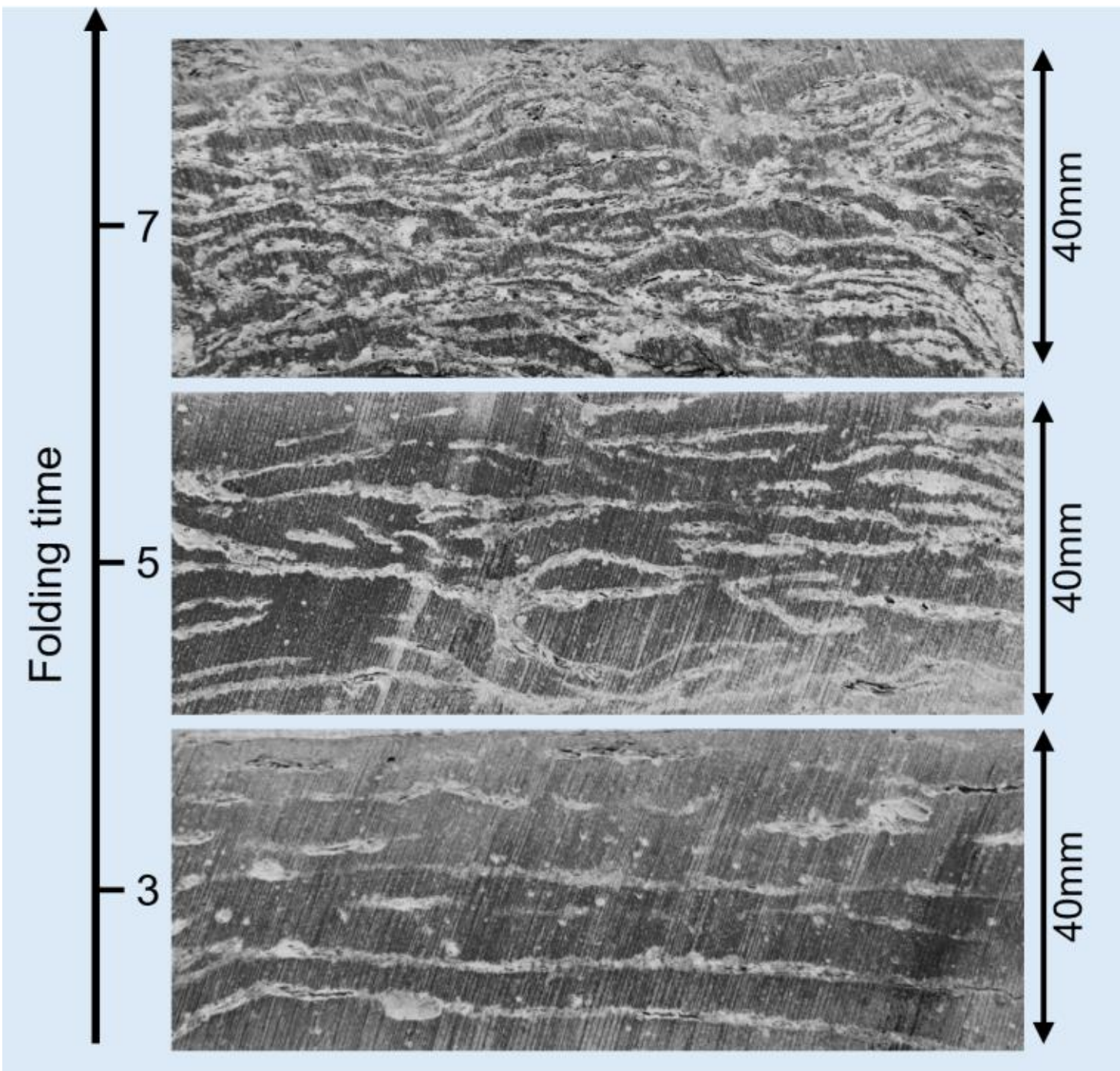

Supplementary Fig. 17. Cross-sections of puff pastry architected cementitious material (PPAC) materials with different rolling-folding cycles. As the number of rolling-folding cycles increases, the number of weak interfaces increases sharply, while the continuous weak interface length and the continuous solid phase thickness decreases.

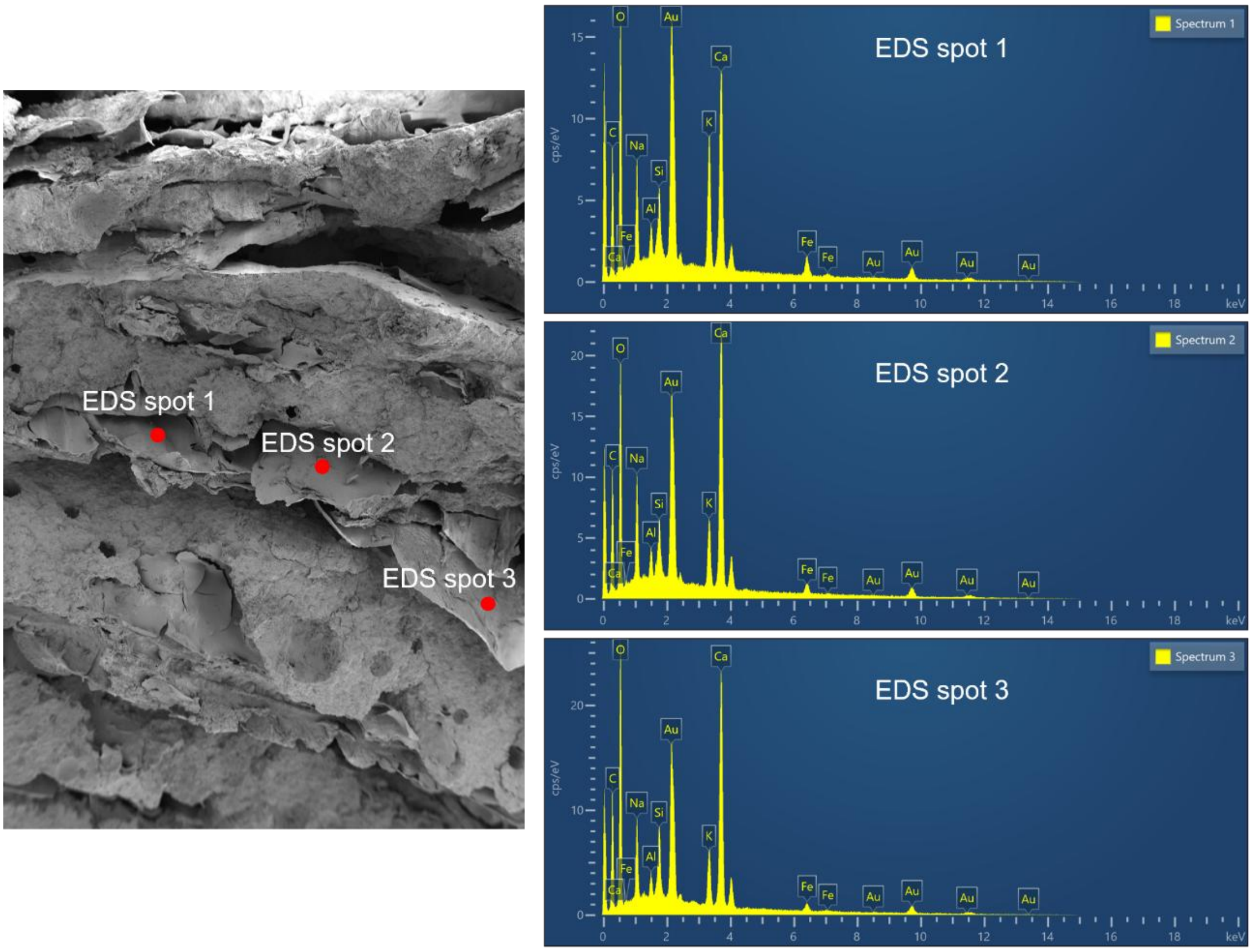

Supplementary Fig. 18. EDS point-scan results. The high carbon atomic ratio confirms that the film-like structures observed in the SEM images correspond to the organic component formed by sodium alginate.

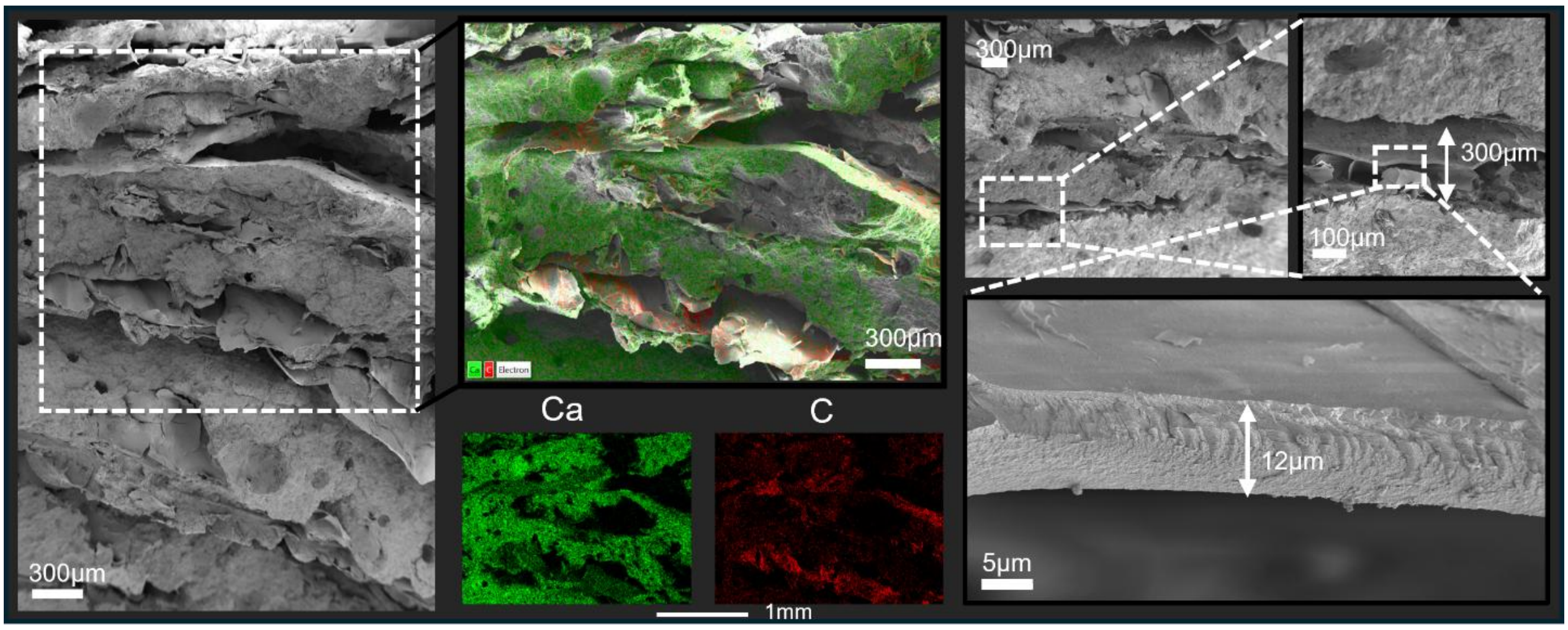

Supplementary Fig. 19. Original version of the SEM & EDS results.

Supplementary Table 3. Elemental composition of EDS spots in supplementary Fig. 13

Spectrum 1			Spectrum 2			Spectrum 3		
**Element**	**Weight %**	**Atomic %**	**Element**	**Weight %**	**Atomic %**	**Element**	**Weight %**	**Atomic %**
C	29.00	41.86	C	28.08	40.70	C	27.96	39.63
O	39.74	43.06	O	40.19	43.73	O	44.14	46.97
Na	4.52	3.41	Na	4.90	3.71	Na	3.83	2.84
Al	1.04	0.67	Al	0.99	0.64	Al	0.80	0.51
Si	1.73	1.07	Si	1.53	0.95	Si	2.04	1.23
K	7.04	3.12	K	3.81	1.70	K	3.30	1.44
Ca	12.71	5.50	Ca	17.94	7.79	Ca	15.97	6.79
Fe	4.23	1.31	Fe	2.55	0.79	Fe	1.96	0.60
Total	100.00	100.00	Total	100.00	100.00	Total	100.00	100.00

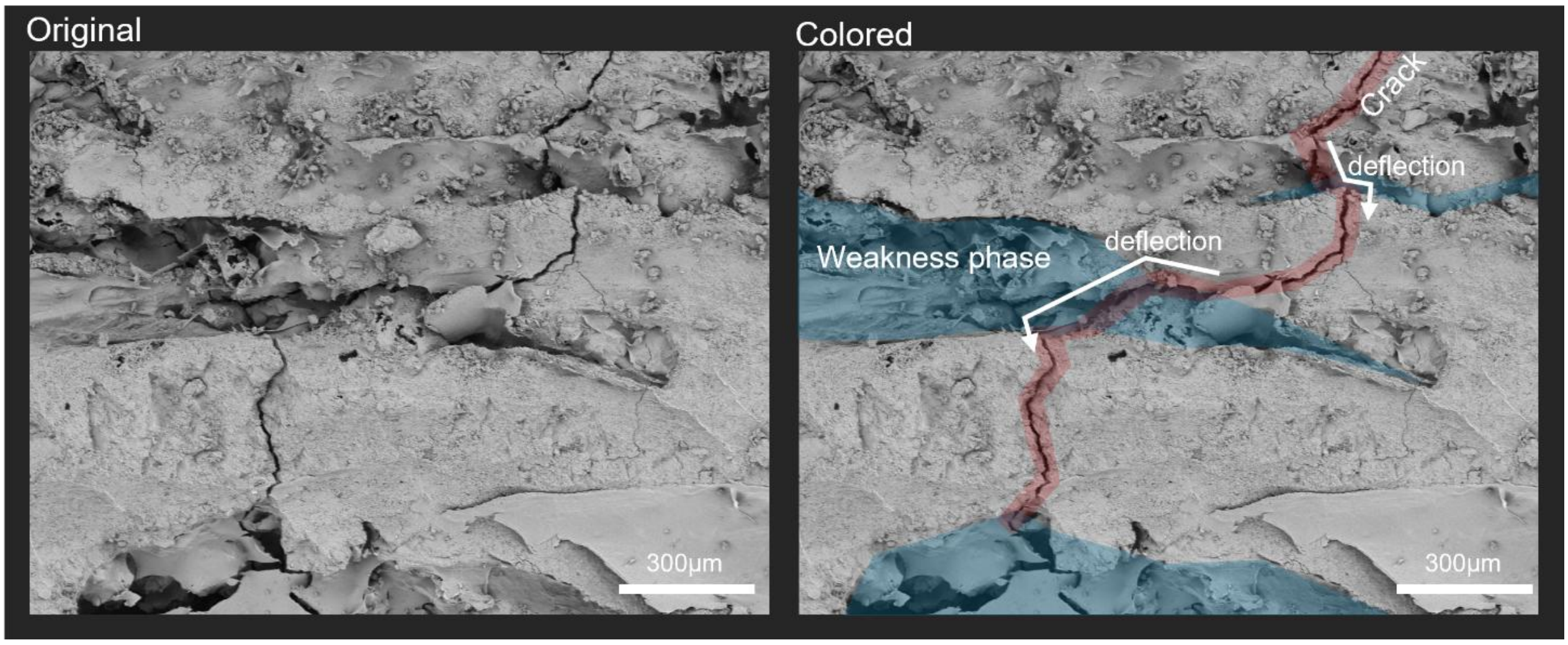

Supplementary Fig. 20. Crack deflection observed in SEM. When the crack propagated through the weakness phase, a pronounced direction deflection can be observed, resulting in a tortuous crack path.

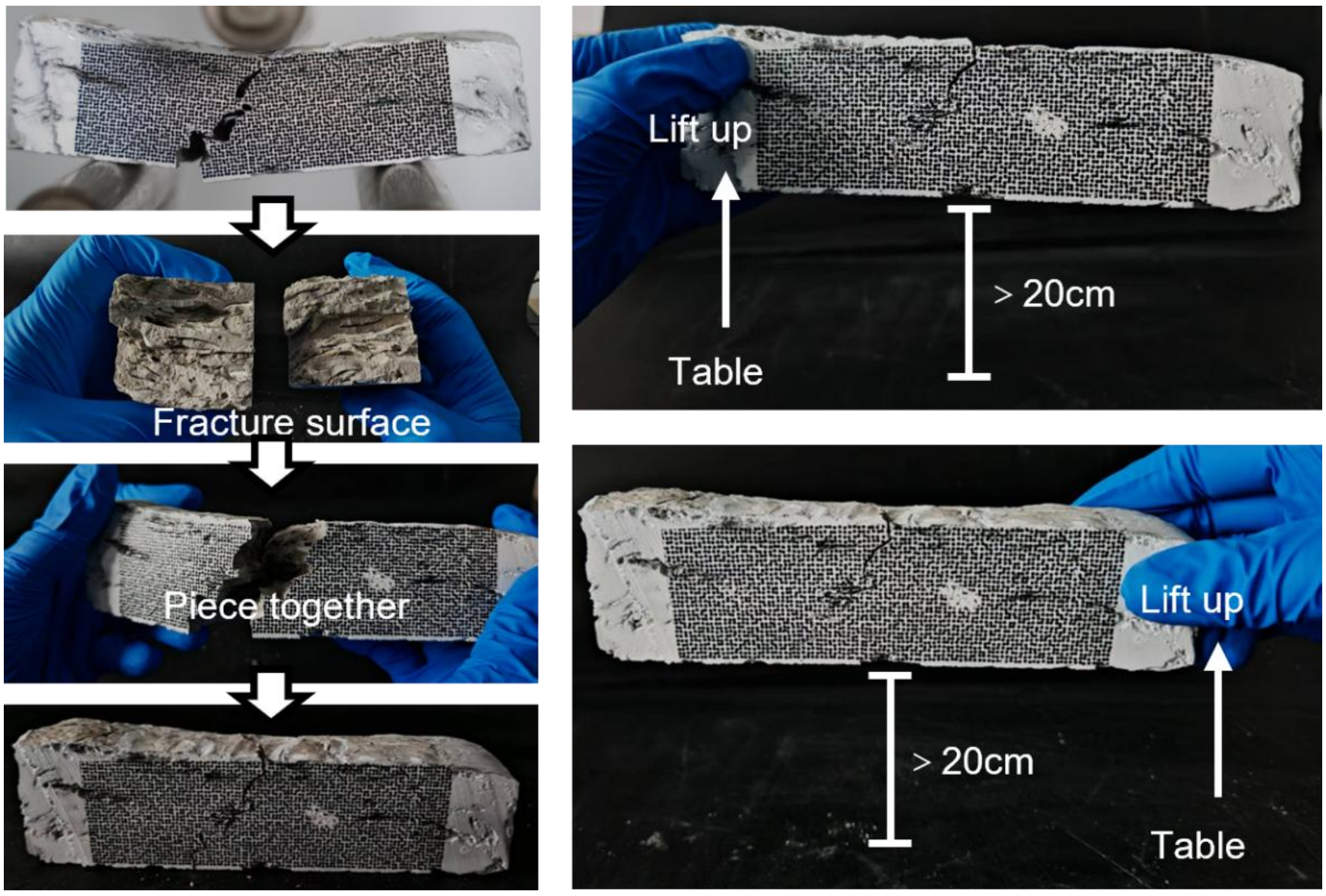

Supplementary Fig. 21. Specimen integrity enabled by mechanical interlocking. After fracture, the fractured parts were pieced together, and when held by one end, it could still be lifted horizontally off the table, demonstrating its structural integrity.

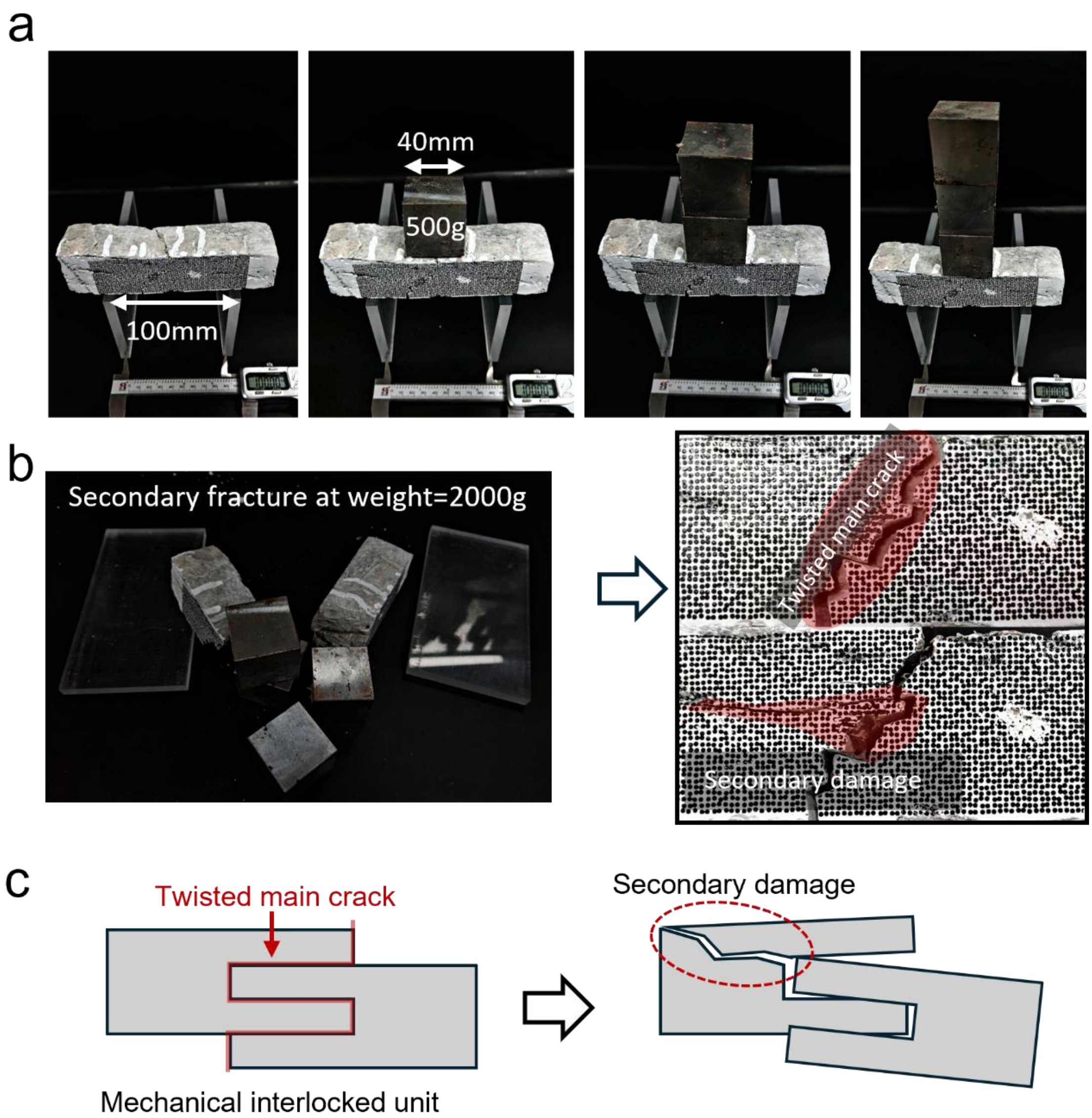

Supplementary Fig. 22. Secondary loading performance of the specimen enabled by mechanical interlocking. a After reassembling the fractured specimen, it could still withstand bending loads. The specimen was placed on two acrylic supports, each 1 cm thick, with a span of 100 mm. Metal cubes (40 mm edge length, 500 g mass) were placed at mid-span. When three cubes were stacked (total mass = 1500 g), the specimen remained intact and continued to bear the load. **b** When the number of metal cubes increased to four (total mass = 2000 g), the specimen collapsed, and secondary fracture occurred at the original fracture surface, forming horizontal cracks. **c** The mechanism of secondary damage arises from the interlocking effect. During bending, the interlocked cement lamellae experience pull-out resistance. When the load becomes excessive, the connection between lamellae and matrix fails, resulting in horizontal cracking.

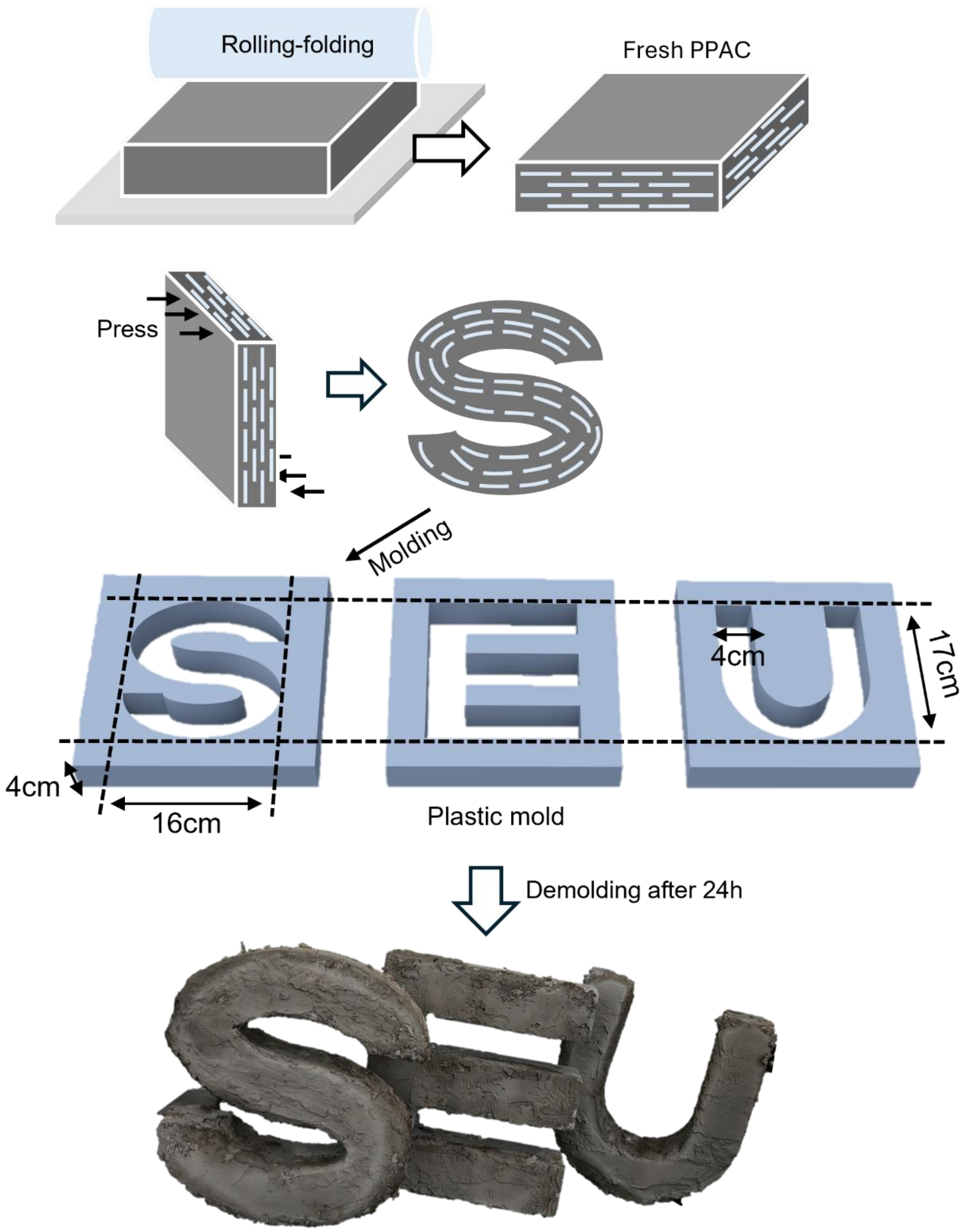

Supplementary Fig. 23. Fabrication of complex-shaped puff pastry architected cementitious material (PPAC) element. Since the time cost for PPAC manufacturing is short, the material remains in a plastic stage after the rolling-folding process. By casting the PPAC into a mold, it can be shaped into complex geometries. The mold shown in the figure was fabricated in advance using polymer 3D printing technology and made of plastic. The letters "SEU" represents the abbreviation of Southeast University.

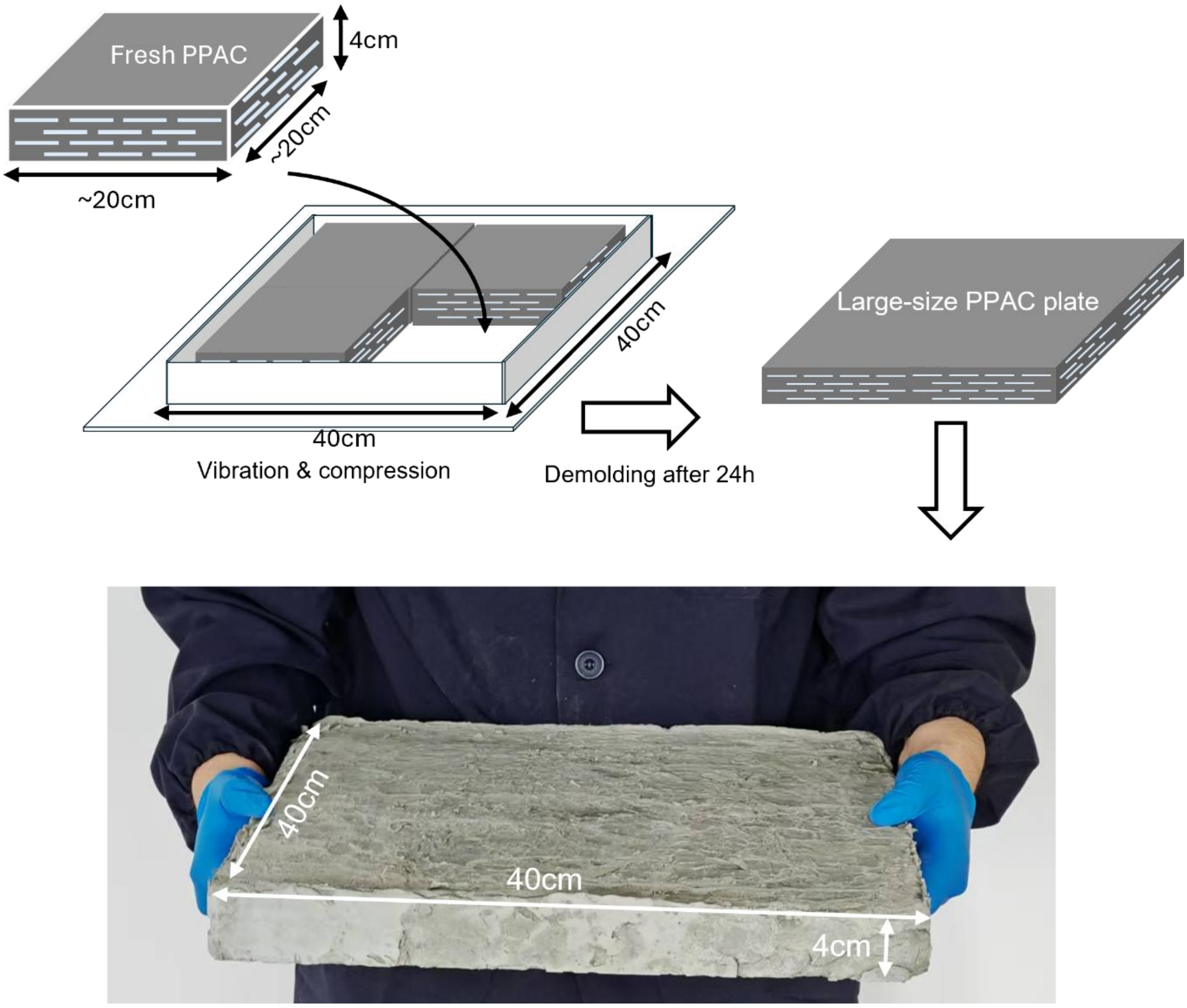

Supplementary Fig. 24. Fabrication of large-scale puff pastry architected cementitious material (PPAC) plate. Multiple PPAC blocks in the plastic stage were placed into a plastic mold measuring 40 cm × 40 cm × 4 cm. The contacting regions between adjacent PPAC blocks bonded together through slight vibration or compression, and after hardening, a large-size PPAC plate was successfully formed.

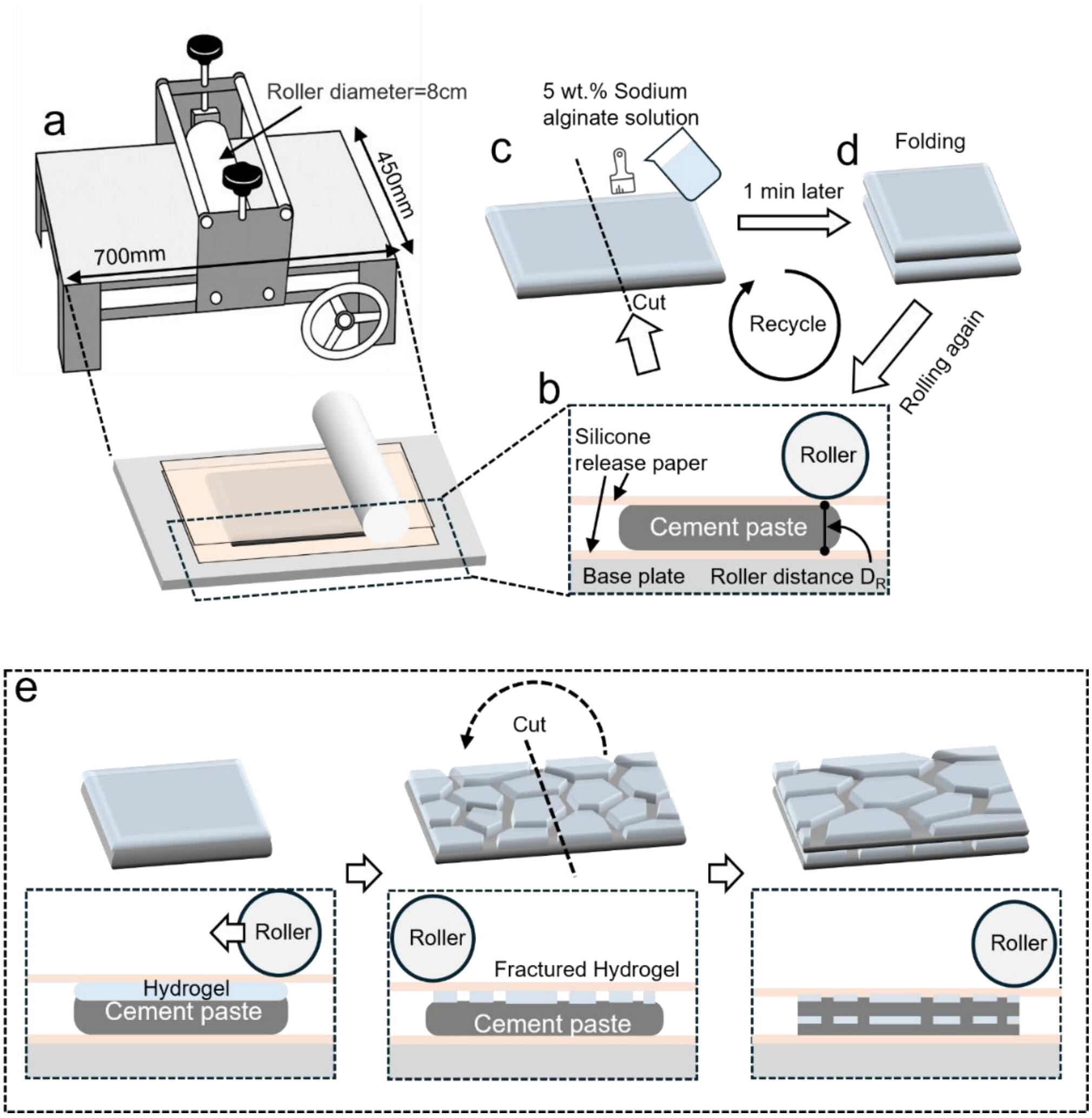

Supplementary Fig. 25. Detailed fabrication process of puff pastry architected cementitious material (PPAC). a A "clay slab roller" was used to perform the rolling-folding process. The base plate for placing the material measured 700 mm × 450 mm, and the roller diameter is 8 cm. **b** Before rolling the cement paste, it was wrapped in silicone paper to prevent adhesion between the paste, the base plate, and the roller. During the rolling process, the distance between the roller and the base plate (DR) was adjusted multiple times. In each rolling–folding cycle of this study, the cement paste was compressed from 40 mm to 20 mm in thickness. To avoid excessive deformation that could damage the PPAC, this reduction was achieved in four rolling steps, decreasing DR by 5 mm each time. **c** After rolling, the cement paste was removed from the silicone paper, and a 5 wt.% sodium alginate solution was brushed on its surface at a dosage of approximately 0.03 g/cm². **d** After 1 minute, the cement paste was cut in half along the middle and folded together, followed by another rolling operation. **e** The sodium alginate can form a hydrogel layer on the surface of the cement paste through in-situ crosslinking. During rolling, the hydrogel will fragment into small pieces due to the expansion of the paste area and applied pressure, exposing parts of the cement paste surface. In subsequent rolling steps, these hydrogel fragments can be embedded between adjacent cement paste layers, forming weak interfaces, while the exposed cement paste regions will bond together, creating interlocking areas that preserved the material's overall integrity.

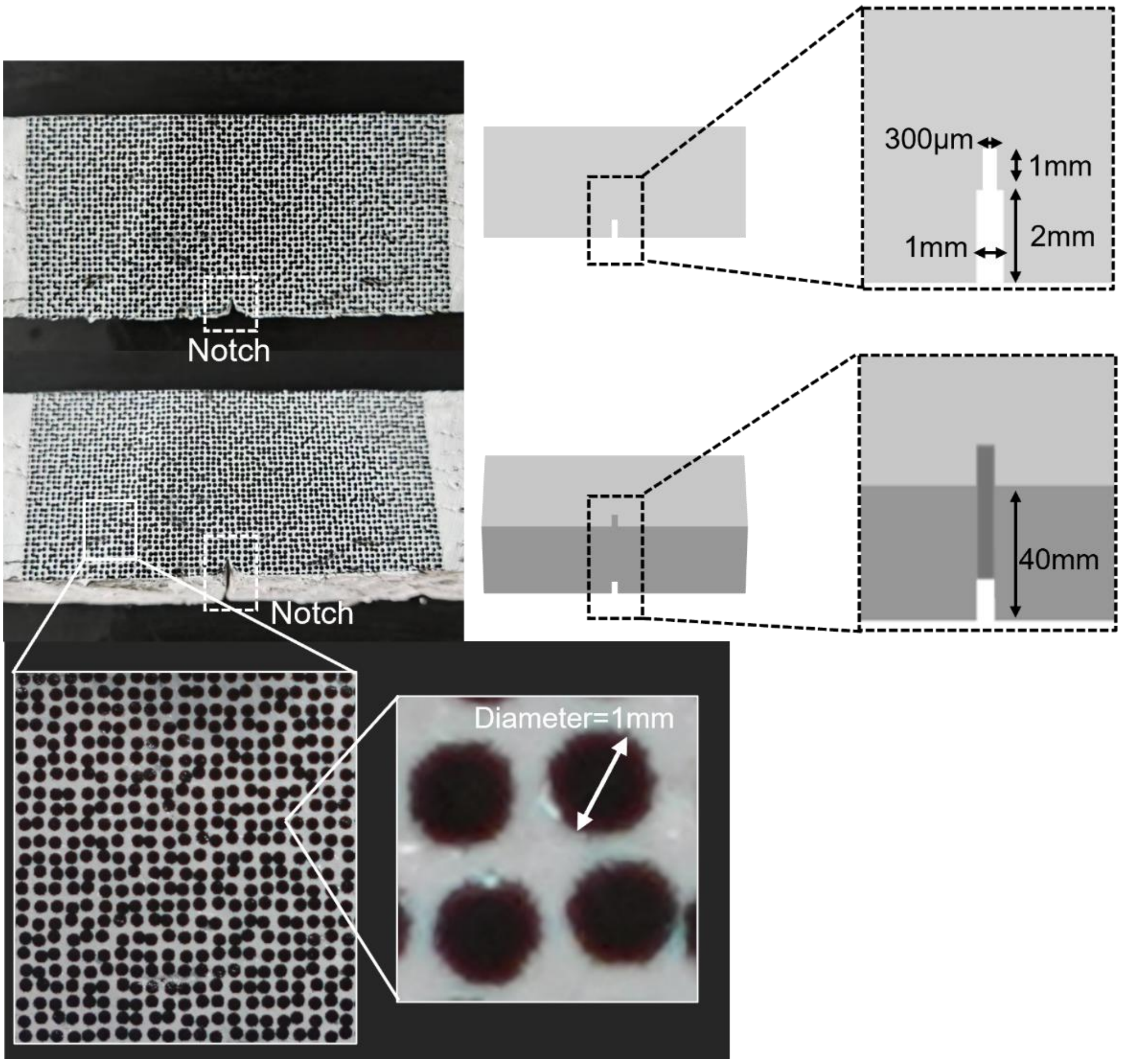

Supplementary Fig. 26. Notch and DIC speckle configurations of the SENB specimens. The notch was first introduced at the bottom of the specimen using a 1 mm thick diamond saw to a depth of 2 mm, followed by extending the notch an additional 1 mm using a 300 μm thick razor blade [6]. The DIC speckle pattern consists of a white background with randomly distributed circular black dots of approximately 1 mm in diameter.

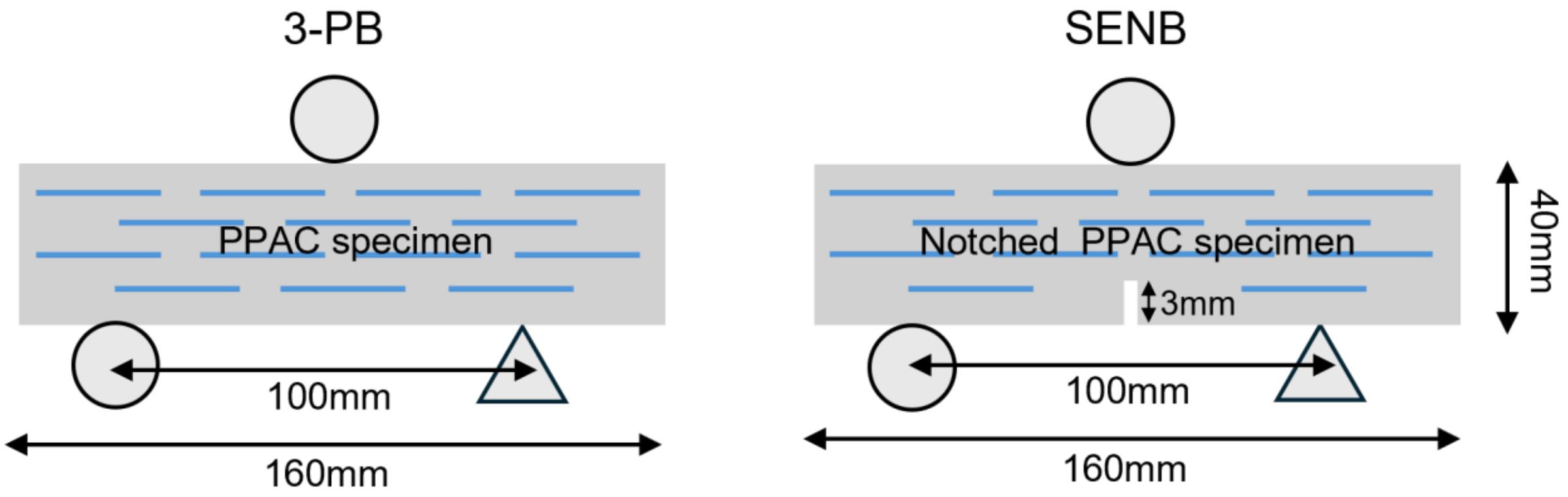

Supplementary Fig. 27. Test setup for 3-point bending (3PB) and single-edge notch bend (SENB). The test setup followed ASTM C293M-16 [7] and ASTM E1820-20b [8]; specimens measured 40 mm × 40 mm × 160 mm, with a span of 100 mm, loaded at 1 mm/min in a direction perpendicular to the lamellar arrangement.

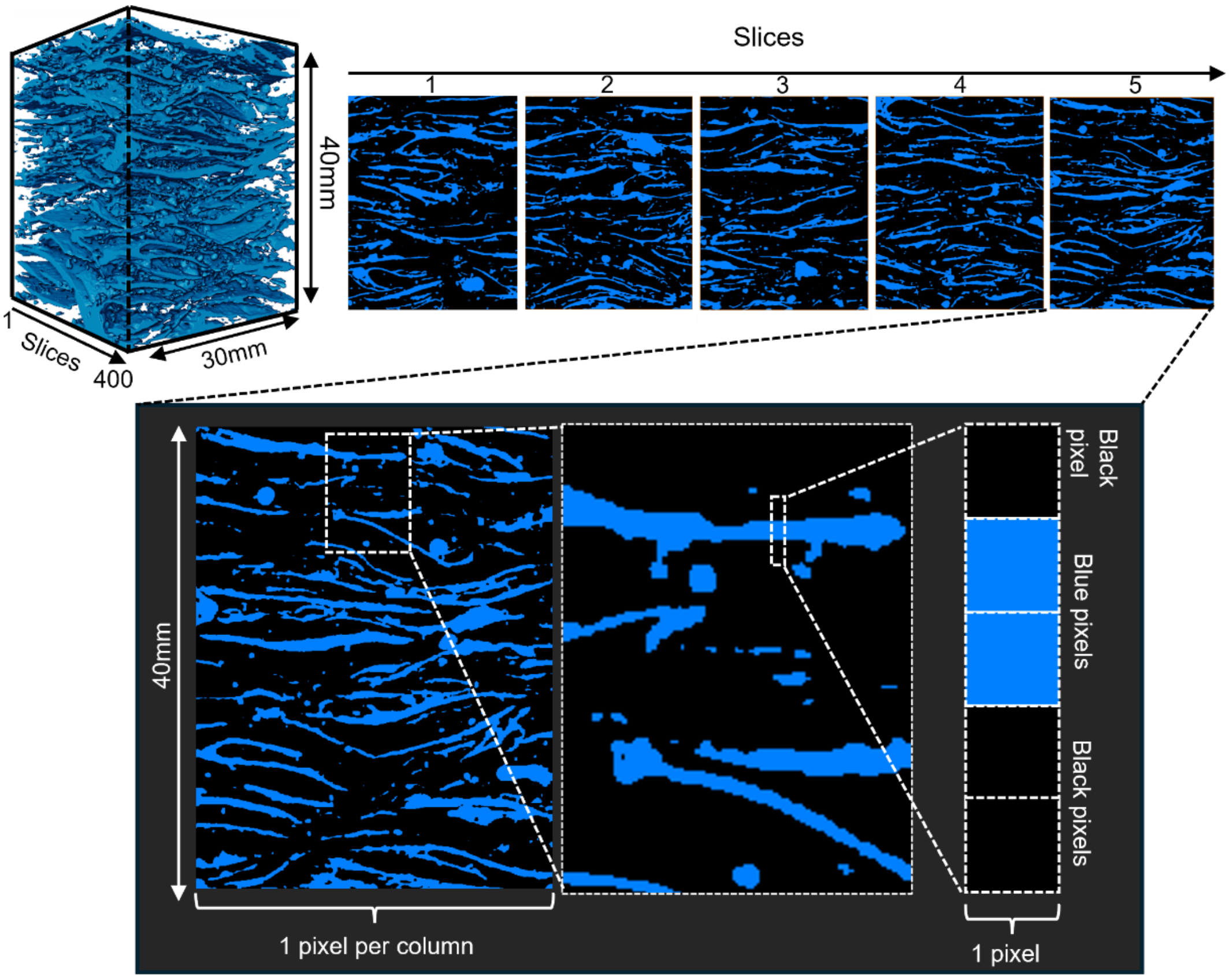

Supplementary Fig. 28. Measurement method for solid phase thickness and weakness phase thickness. The X-CT 3D reconstructed model was sliced along the direction perpendicular to the folding orientation. Based on the resolution of the X-CT scan, approximately 400 slices were obtained. Five slices were selected at equal intervals—specifically, the 1st, 100th, 200th, 300th, and 400th slices. These slices were then pixelated for further analysis. For each slice, pixel columns were defined with a width of one pixel. For each pixel column, the pixels were traversed from top to bottom, and their colors were identified. When a black pixel was detected, the number of consecutive black pixels was recorded; if the following pixel was also black, the count continued. When the next pixel was blue, the counting stopped, and the recorded number of black pixels was defined as one recorded solid phase thickness for that pixel column. The same procedure was applied to blue pixels to determine the weak phase thickness. This process was performed for every pixel column in each selected slice, and the final statistical results were compiled to obtain the distribution frequency of the solid and weak phase thicknesses. The entire workflow can be automated by writing a Python program.

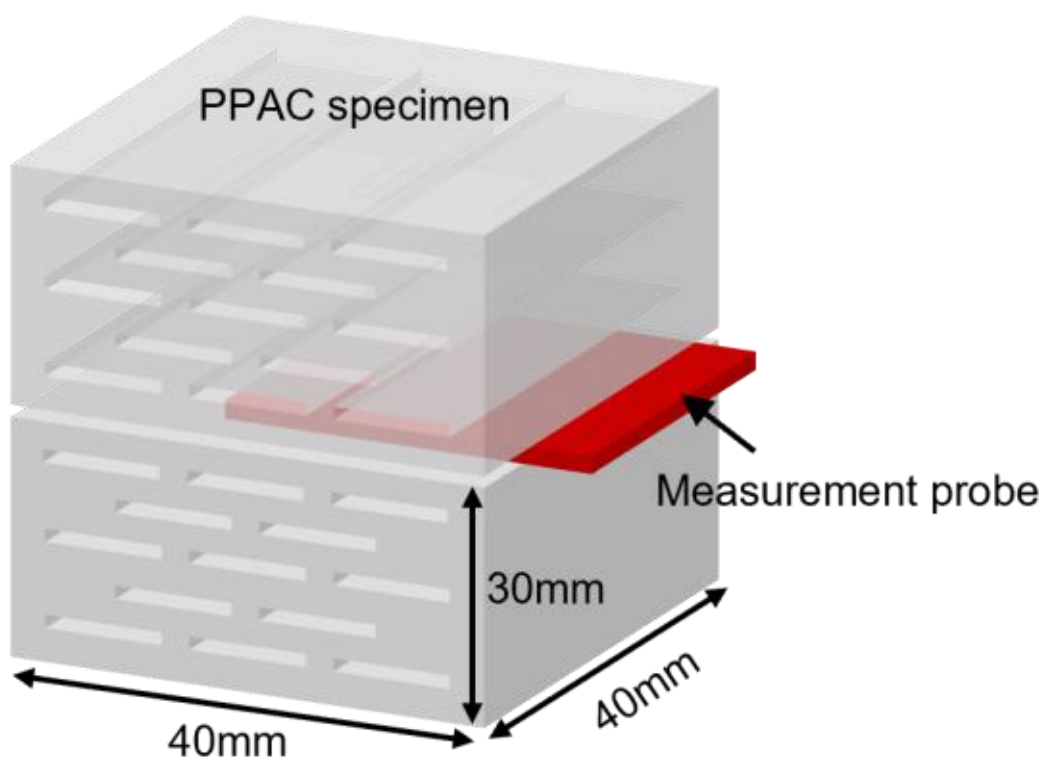

Supplementary Fig. 29. Specimen configuration for hot disk test. The specimens possessed the same dimensions as those in the fire & thermal resistance tests, measuring 40 mm× 40 mm× 30 mm. Two identical samples were used to clamp the measurement probe, with the clamping surfaces parallel to the lamellar arrangement.

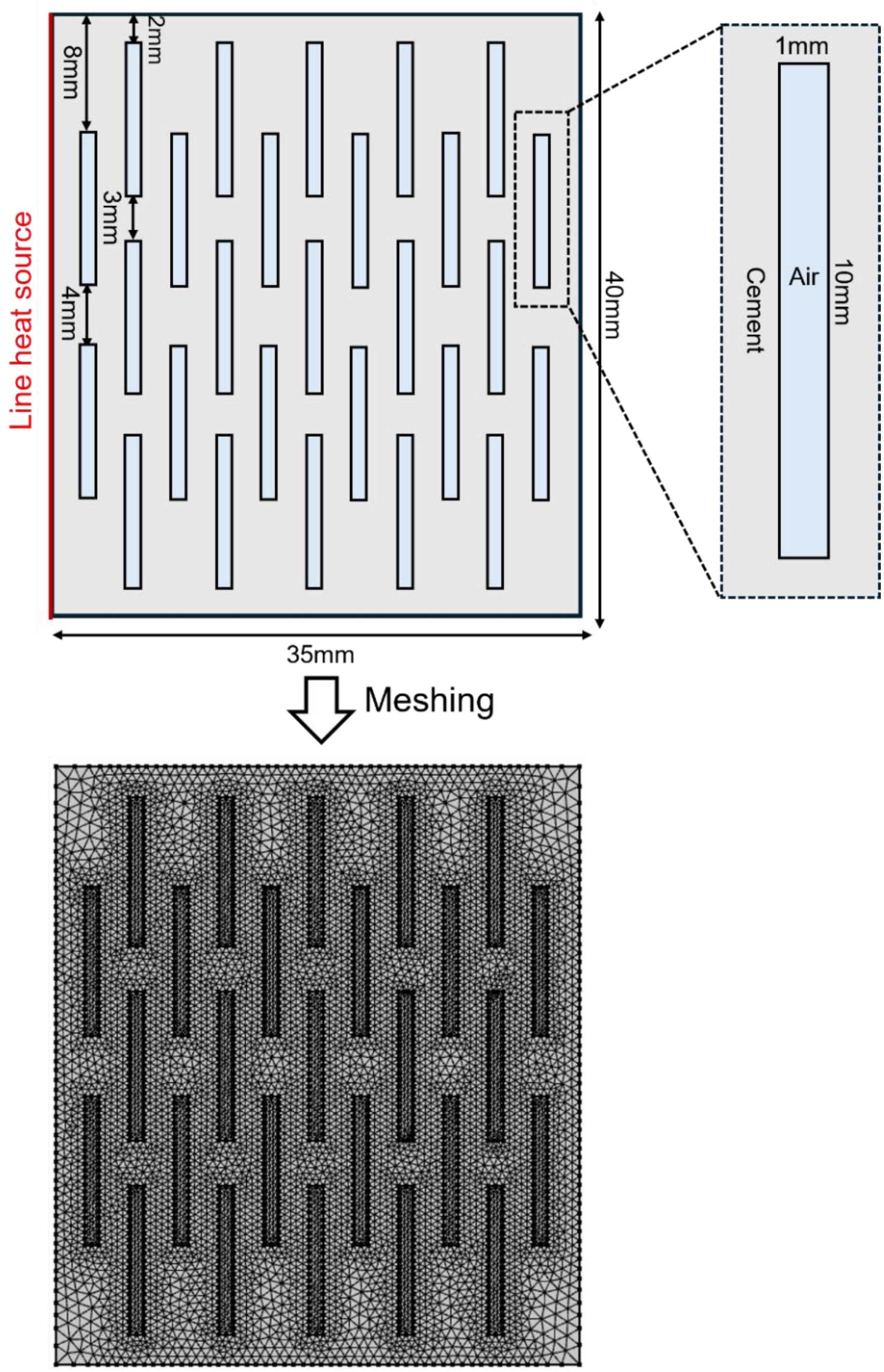

Supplementary Fig. 30. Multiphysics heat transfer simulation model. According to the specimen used for thermal resistance test in this study, the simulation model was constructed with dimensions of 35 mm×40 mm. Based on the length-to-thickness ratio of the weakness phase observed in puff pastry architected cementitious material (PPAC) Fold 7 (see Supplementary Fig. 16), the weakness phase in the model was simplified as a rectangular region with length to width ratio of 10:1. Considering the resolution limitations of the simulation, the weakness phase was simplified to 10 mm×1 mm. The number of weakness phases was determined by simplifying according to the density ratio between PPAC-Fold 7 and Cast, resulting in a volume fraction of approximately 20% for the weakness phase relative to the solid phase. In the simulation, solid–fluid conjugate heat transfer and surface-to-surface radiation interfaces were employed. The weakness phase was modeled as air, while the solid phase was modeled as the cement matrix, with thermal conductivity values based on experimental measurements. The radiative emissivity of the cement matrix was set to 0.95 according to Refs. [9-11].

Supplementary Table 4. Data set and t-test significance analysis results of the strain at failure in Fig. 2 b.

Sub Table a. Data set

Specimen	Mean value	SD	Size (n)
Cast	0.01368	0.00291	3
Fold-3	0.10712	0.02297	3
Fold-5	0.11792	0.01751	3
Fold-7	0.28736	0.04024	3

Sub Table b. Significance analysis

Comparison	Mean difference	T-value	df	p-value	Significance
Fold-3 vs Cast	0.09344	7.02	2	0.018	*
Fold-5 vs Cast	0.10424	10.20	2	0.008	**
Fold-7 vs Cast	0.27368	11.78	2	0.006	**
Fold-3 vs Fold-5	0.01080	0.64	3	0.56	n.s.
Fold-3 vs Fold-7	0.18024	6.74	2	0.019	*
Fold-5 vs Fold-7	0.16944	6.72	2	0.019	*

* ($p<0.05$) indicates a significant difference; ** ($p<0.01$) indicates a highly significant difference; n.s. means no significant difference.

Supplementary Table 5. Data set and t-test significance analysis results of the density in Fig. 2 c.

Sub Table a. Data set

Specimen	Mean value	SD	Size (n)
Cast	2018	32	3
Fold-3	1988	38	3
Fold-5	1809	35	3
Fold-7	1667	29	3

Sub Table b. Significance analysis

Comparison	Mean difference	T-value	df	p-value	Significance
Fold-3 vs Cast	−30	−1.05	3	0.38	n.s.
Fold-5 vs Cast	−209	−7.63	2	0.018	*
Fold-7 vs Cast	−351	−14.08	2	0.006	**
Fold-3 vs Fold-5	179	6.00	2	0.025	*
Fold-3 vs Fold-7	321	11.63	2	0.008	**
Fold-5 vs Fold-7	142	5.41	2	0.030	*

* ($p<0.05$) indicates a significant difference; ** ($p<0.01$) indicates a highly significant difference; n.s. means no significant difference.

Supplementary Table 6. Data set and t-test significance analysis results of the specific MOR in Fig. 2 d.

Sub Table a. Data set

Specimen	Mean value	SD	Size (n)
Cast	1.53281	0.12	3
Fold-3	1.51172	0.19	3
Fold-5	1.48594	0.13	3
Fold-7	1.56797	0.15	3

Sub Table b. Significance analysis

Comparison	Mean difference	T-value	df	p-value	Significance
Fold-3 vs Cast	−0.02109	−0.163	2	0.88	n.s.
Fold-5 vs Cast	−0.04687	−0.459	2	0.67	n.s.
Fold-7 vs Cast	0.03516	0.317	2	0.77	n.s.
Fold-3 vs Fold-5	0.02578	0.194	2	0.86	n.s.
Fold-3 vs Fold-7	0.05625	0.403	2	0.71	n.s.
Fold-5 vs Fold-7	0.08203	0.716	2	0.52	n.s.

* ($p<0.05$) indicates a significant difference; ** ($p<0.01$) indicates a highly significant difference; n.s. means no significant difference.

Supplementary Table 7. Data set and t-test significance analysis results of the specific K_{IC} in Fig. 2 f.

Sub Table a. Data set

Specimen	Mean value	SD	Size (n)
Cast	4.73244	0.42	3
Fold-3	4.68968	0.37	3
Fold-5	4.81797	0.29	3
Fold-7	5.10306	0.38	3

Sub Table b. Significance analysis

Comparison	Mean difference	T-value	df	p-value	Significance
Fold-3 vs Cast	−0.04276	−0.132	3	0.90	n.s.
Fold-5 vs Cast	0.08553	0.29	3	0.78	n.s.
Fold-7 vs Cast	0.37062	1.134	3	0.35	n.s.
Fold-3 vs Fold-5	0.12829	0.473	3	0.67	n.s.
Fold-3 vs Fold-7	0.41338	1.35	3	0.29	n.s.
Fold-5 vs Fold-7	0.28509	1.03	3	0.40	n.s.

* ($p<0.05$) indicates a significant difference; ** ($p<0.01$) indicates a highly significant difference; n.s. means no significant difference.

Supplementary Table 8. Data set and t-test significance analysis results of the flexural toughness in Fig. 2 g.

Sub Table a. Data set

Specimen	Mean value	SD	Size (n)
Cast	10.4	1.28	3
Fold-3	42.9	3.24	3
Fold-5	52.4	7.53	3
Fold-7	191.8	13.52	3

Sub Table b. Significance analysis

Comparison	Mean difference	T-value	df	p-value	Significance
Fold-3 vs Cast	32.5	16.17	2	0.003	**
Fold-5 vs Cast	42.0	9.52	2	0.009	**
Fold-7 vs Cast	181.4	23.11	2	0.001	**
Fold-3 vs Fold-5	9.5	2.01	2	0.16	n.s.
Fold-3 vs Fold-7	148.9	18.54	2	0.002	**
Fold-5 vs Fold-7	139.4	15.59	2	0.003	**

* ($p<0.05$) indicates a significant difference; ** ($p<0.01$) indicates a highly significant difference; n.s. means no significant difference.

Supplementary Table 9. Data set and t-test significance analysis results of the Specific K_{JC} in Fig. 2 h.

Sub Table a. Data set

Specimen	Mean value	SD	Size (n)
Cast	4.73	0.52	3
Fold-3	20	2.5	3
Fold-5	17	4.5	3
Fold-7	91	7.3	3

Sub Table b. Significance analysis

Comparison	Mean difference	T-value	df	p-value	Significance
Fold-3 vs Cast	15.27	10.36	2	0.008	**
Fold-5 vs Cast	12.27	4.69	2	0.041	*
Fold-7 vs Cast	86.27	20.39	2	0.002	**
Fold-3 vs Fold-5	3	1.01	2	0.40	n.s.
Fold-3 vs Fold-7	71	15.92	2	0.003	**
Fold-5 vs Fold-7	74	14.95	2	0.003	**

* ($p<0.05$) indicates a significant difference; ** ($p<0.01$) indicates a highly significant difference; n.s. means no significant difference.

Supplementary Table 10. Data set and t-test significance analysis results of the thermal conductivity in Fig. 3 e.

Sub Table a. Data set

Specimen	Mean value	SD	Size (n)
Cast	1.2	0.12	3
Lightweight concrete	0.73	0.15	3
Fold-7	0.38	0.05	3

Sub Table b. Significance analysis

Comparison	Mean difference	T-value	df	p-value	Significance
Lightweight concrete vs Cast	−0.47	−4.24	3	0.021	*
Fold-7 vs Cast	−0.82	−10.93	2	0.005	**
Fold-7 vs Foam concrete	−0.35	−3.83	2	0.028	*

* ($p<0.05$) indicates a significant difference; ** ($p<0.01$) indicates a highly significant difference; n.s. means no significant difference.

Supplementary discussion 1. Compressive properties of PPAC.

Due to the presence of the internal sheet-like cavities, the compressive strength is lower than that of cast counterparts with the same mix proportion (**Supplementary Fig. 31 b**). In addition, owing to the pronounced orientation of its lamellar structure, the compressive strength of PPAC shows significant anisotropy between the in-plane and out- plane directions (**Supplementary Fig. 31 a**). Specifically, when the load is applied parallel to the weak interfaces and lamellar structure (in-plane direction), the compressive strength is markedly higher than when the load is applied perpendicular to the lamellar structure (out-plane direction). The underlying mechanism is reasonably straightforward. Under out-plane loading, stress concentration is more likely to occur at the tips of the thin interlayer cavities[12], and the interlocking regions between lamellae become the critical sites for failure. In contrast, when loading is applied in the in-plane direction, stress concentration within the sheet-like cavities is alleviated, and the lamellae can dominate the failure process, resulting in a larger effective load-bearing area (**Supplementary Fig. 31 a**), thereby leading to higher compressive strength (**Supplementary Fig. 31 b**). However, for the unique compressive behavior observed in PPAC, we only provide a preliminary interpretation based on the experimental results, and the underlying mechanisms require further investigation.

Considering that compressive strength is also important for infrastructure materials in practical engineering applications, we conducted preliminary investigations into several approaches to enhance compressive strength by adjusting material composition, include:

1. Mortar: Sand was introduced into the cement paste system. The sand used was fine quartz sand with a maximum particle size of approximately 0.6 mm, while all other components remained consistent with those used in the cement paste-based PPAC. The detailed mix proportions are as follows:

Supplementary Table 11. Mix proportion of PPAC-cement mortar (g/1000g binder)

Cement	Water	Sand	PCE	HPMC
1000	350	1500	3.5	20

2. UHPC mortar: PPAC was fabricated using UHPC mortar without steel fibers. Based on the previously described cement mortar system, silica fume and slag were further incorporated. The detailed mix proportions are as follows:

Supplementary Table 12. Mix proportion of PPAC-UHPC mortar (g/1000g binder)

Cement	Silica fume	Fly ash	PCE	Water	Sand
680	140	180	14	170	900

The experimental results indicate that the utilization of UHPC mortar can effectively enhance the compressive strength of PPAC, especially when loading is applied along the in-plane direction (**Supplementary Fig. 31 b**). The higher strength in the in-plane direction may be advantageous for certain engineering applications (**Supplementary Fig. 31 c**). For example, when used as a wall material to exploit its thermal insulation properties, the lamellar voids would be oriented perpendicular to the ground, and the primary gravitational load would be applied parallel to the weak interfaces. In contrast, when subjected to out-plane loading, such as in roofs or slabs, the imposed loads (e.g., human activity or snow loads) are typically much smaller than the self-weight of the structure, and structural performance is more strongly governed by flexural strength.

These results demonstrate the tunability and controllability of PPAC, as well as its potential for further performance enhancement. However, it should be noted that the primary objective of this study is to

propose the underlying concept. More detailed optimization strategies and material design considerations will require more comprehensive and systematic investigations in future work.

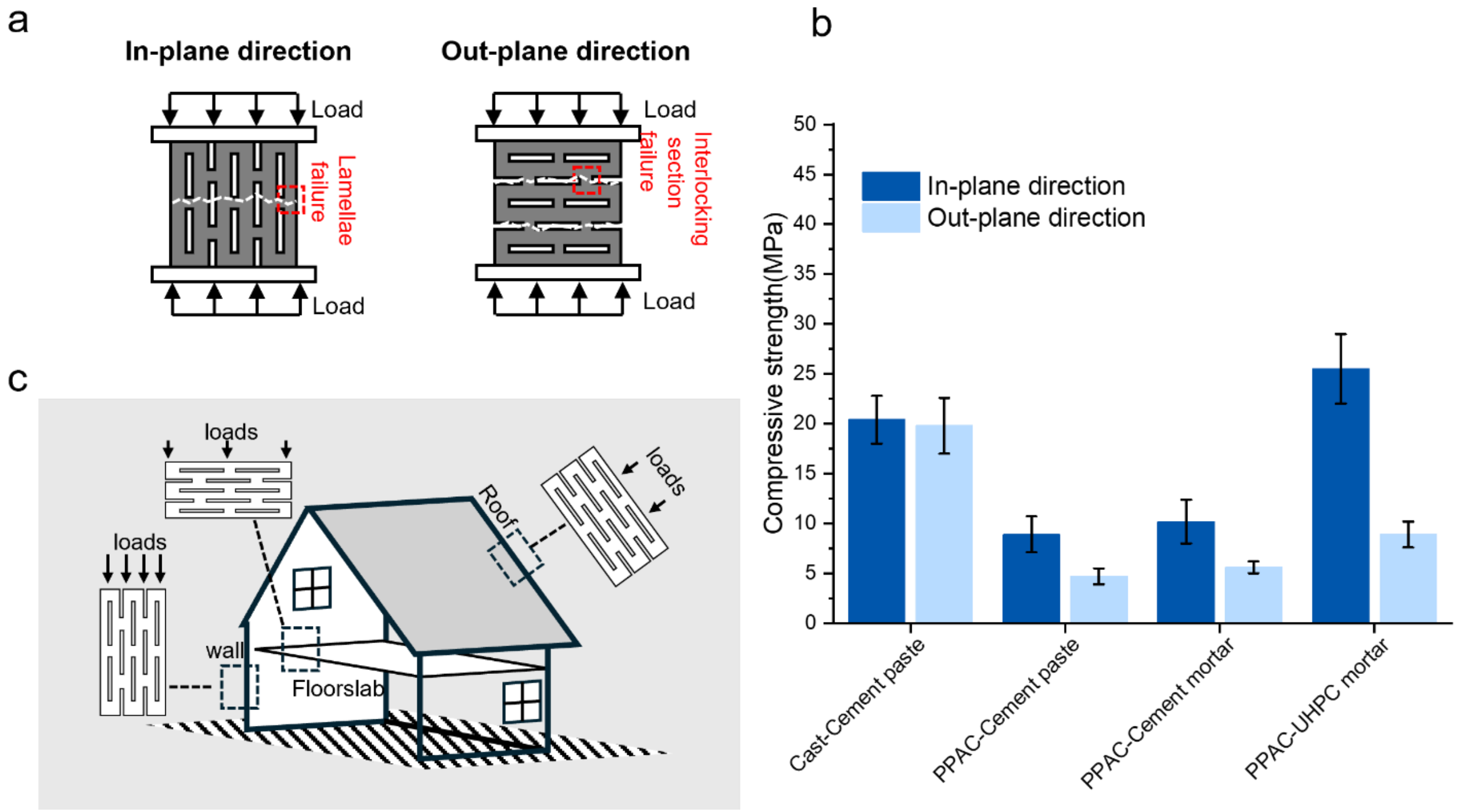

Supplementary Fig. 31. Compressive strength of PPAC from different loading directions. a Schematic illustration of in-plane and out-plane loading directions. **b** Compressive strength test results of PPAC specimens fabricated with different paste compositions. Specimens with dimensions of about 40mm×40mm×40mm were used. Data are presented as mean ± SD from n=3 independent specimens. The Folding-time of these specimens are 7. **c** Schematic diagram of potential loading situations in housing applications.

Supplementary discussion 2. Size effect on PPAC performance.

To evaluate the influence of size effect on PPAC performance, specimens with different thicknesses (20 mm, 40 mm and 60 mm, **Supplementary Fig. 32 a**) were investigated. The 40 mm data correspond to the PPAC fold-7 specimens presented in the main text. To minimize variables, all specimens were prepared following the same procedure. Specifically, specimens were first fabricated using the protocol described in the main text with 7 rolling–folding cycles (same as PPAC Fold-7). During the plastic stage, two 40 mm thick specimens were stacked and cured to obtain an 80 mm thick specimen, which was subsequently cut to 60 mm. The 20 mm thick specimens were obtained by cutting the original 40 mm thick PPAC fold-7 specimens. This preparation strategy ensures that all specimens share a comparable layered architecture, with specimen thickness being the primary variable.

Single-edge notched bending (SENB) tests were then performed (**Supplementary Fig. 32 b**). The notch depth and span-to-thickness ratio were kept consistent with those used in the main text. The results show that the K_{IC} values do not exhibit significant differences across different thicknesses (**Supplementary Fig. 32 c, Supplementary Table 11**), which is expected, as crack initiation is primarily governed by intrinsic material properties rather than specimen dimensions. The load–displacement curves (**Supplementary Fig. 32 b**) indicate that the 20 mm thick specimens exhibit smaller deflection at failure, mainly due to their reduced thickness.

Due to the differences in specimen thickness, ASTM limit was not used to compare K_{JC} values. Instead, the K_{JC} values at final failure were used (**Supplementary Fig. 32 d**). The results show that the 60 mm and 40 mm thick specimens exhibit no significant difference in K_{JC} values, whereas the 20 mm thick specimens show significantly lower K_{JC} (**Supplementary Fig. 32 e, Supplementary Table 12**). This behavior can be attributed to the underlying toughening mechanism of PPAC, which relies on crack deflection along weak interlayer interfaces. When the layer thickness remains similar, thicker specimens can contain more interfaces along the crack propagation path, leading to increased energy dissipation and enhanced toughness. In contrast, the 20 mm thick specimens likely contain approximately half the number of layers compared to the 40 mm thick counterparts, resulting in reduced toughening effect. Meanwhile, the absence of further improvement from 40 mm to 60 mm suggests that the relationship between interface number and toughness may not be strictly linear.

Overall, these results indicate the dependence of PPAC performance on specimen thickness and the number of internal layers. However, this analysis remains preliminary. A comprehensive understanding of size effects will require more extensive experimental data and systematically designed test groups, which warrants further investigation.

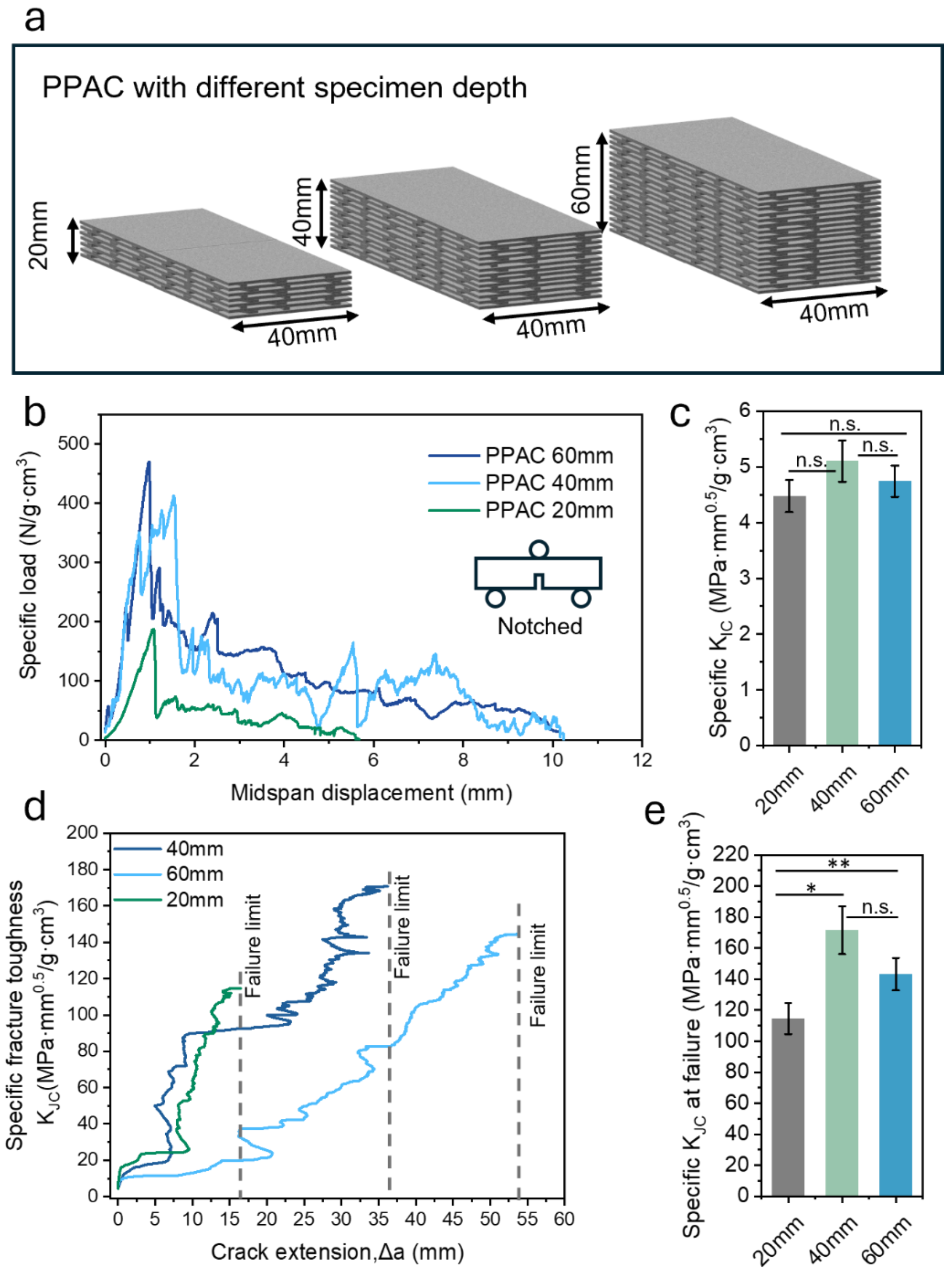

Supplementary Fig. 32 Size effect on the mechanical properties of PPAC. a Schematic diagram of specimen dimension. **b** SENB test results of PPAC with different specimen depth. **c** Specific K_{IC} results. **d** Crack-resistance curves (J-R curves) of PPAC with different specimen depth. **e** Specific K_{JC} value at failure limit. Data in the bar charts are presented as mean ± SD from three independent specimens (n=3). * depicts $p < 0.05$ and ** depicts $p < 0.01$, which indicate statistically significant differences. *P* value is obtained from T-test.

Supplementary Table 13. Data set and t-test significance analysis results of the Specific K_{IC} ($MPa \cdot mm^{0.5}/g \cdot cm^3$) results in Supplementary Fig. 32

Sub Table a. Data set

Specimen	Mean value (Specific K_{IC})	SD	Size (n)
20mm	4.48053	0.29358	3
40mm	5.10306	0.37649	3
60mm	4.76486	0.27802	3

Sub Table b. Significance analysis

Comparison	Mean difference	T-value	df	p-value	Significance
40 mm vs 20 mm	0.62253	2.37	4	0.091	n.s.
60 mm vs 20 mm	0.28433	1.22	4	0.290	n.s.
40 mm vs 60 mm	0.33820	1.34	4	0.284	n.s.

* (p<0.05) indicates a significant difference; ** (p<0.01) indicates a highly significant difference; n.s. means no significant difference.

Supplementary Table 14. Data set and t-test significance analysis results of the Specific K_{JC}-failure ($MPa \cdot mm^{0.5}/g \cdot cm^3$) results in Supplementary Fig. 32

Sub Table a. Data set

Specimen	Mean value (Specific K_{JC} at failure)	SD	Size (n)
20mm	114.89	10.01	3
40mm	171.53	15.38	3
60mm	143.21	10.43	3

Sub Table b. Significance analysis

Comparison	Mean difference	T-value	df	p-value	Significance
40 mm vs 20 mm	56.64	5.35	3	0.009	**
60 mm vs 20 mm	28.32	3.39	4	0.027	*
40 mm vs 60 mm	28.32	2.64	4	0.066	n.s.

* (p<0.05) indicates a significant difference; ** (p<0.01) indicates a highly significant difference; n.s. means no significant difference.

Supplementary discussion 3. Durability of PPAC

Considering the long-term service conditions of infrastructure, durability is a critical property of cementitious materials. Moisture ingress is one of the primary causes of durability-related degradation[13]. In PPAC, the presence of numerous weak interfaces can facilitate water transport, as these interfaces may act as preferential pathways for moisture diffusion (**Supplementary Fig. 33 a**). This study primarily aims to establish and validate the toughness and thermal insulation performance of PPAC, and thus does not present a systematic investigation of durability. However, here we offer preliminary insights and explore the feasibility of potential strategies for improving its durability. Surface hydrophobic treatment is a commonly adopted and effective strategy to improve the durability of cementitious materials[14, 15]. We applied similar approach to PPAC. First, the PPAC specimens were dried to constant mass. Subsequently, a commercial sodium methyl silicate–based silane hydrophobic agent was uniformly applied to the specimen surfaces. After 24 hours, the PPAC surface exhibited pronounced hydrophobicity. Even in regions with weak interfaces, water droplets were effectively prevented from penetrating into the material (**Supplementary Fig. 33 b, c**). To quantitatively evaluate water absorption, untreated cast specimens, untreated PPAC specimens, and treated PPAC specimens were dried and tested according to ASTM C1585. The specimens were placed on a support within a water reservoir, allowing one surface to be in contact with water, and the mass change was measured at specified time intervals to determine water uptake. The water exposure direction for PPAC was consistent with that shown in **Supplementary Fig. 33 c**. As shown in **Supplementary Fig. 33 d**, due to the presence of weak interfaces, PPAC exhibits a higher water absorption rate than the cast counterpart. However, after hydrophobic treatment, the water absorption of PPAC decreases remarkably and becomes notably lower than that of the cast specimens. These results demonstrate that hydrophobic protection can be an effective strategy for enhancing the durability of PPAC and provides a promising direction for its future development.

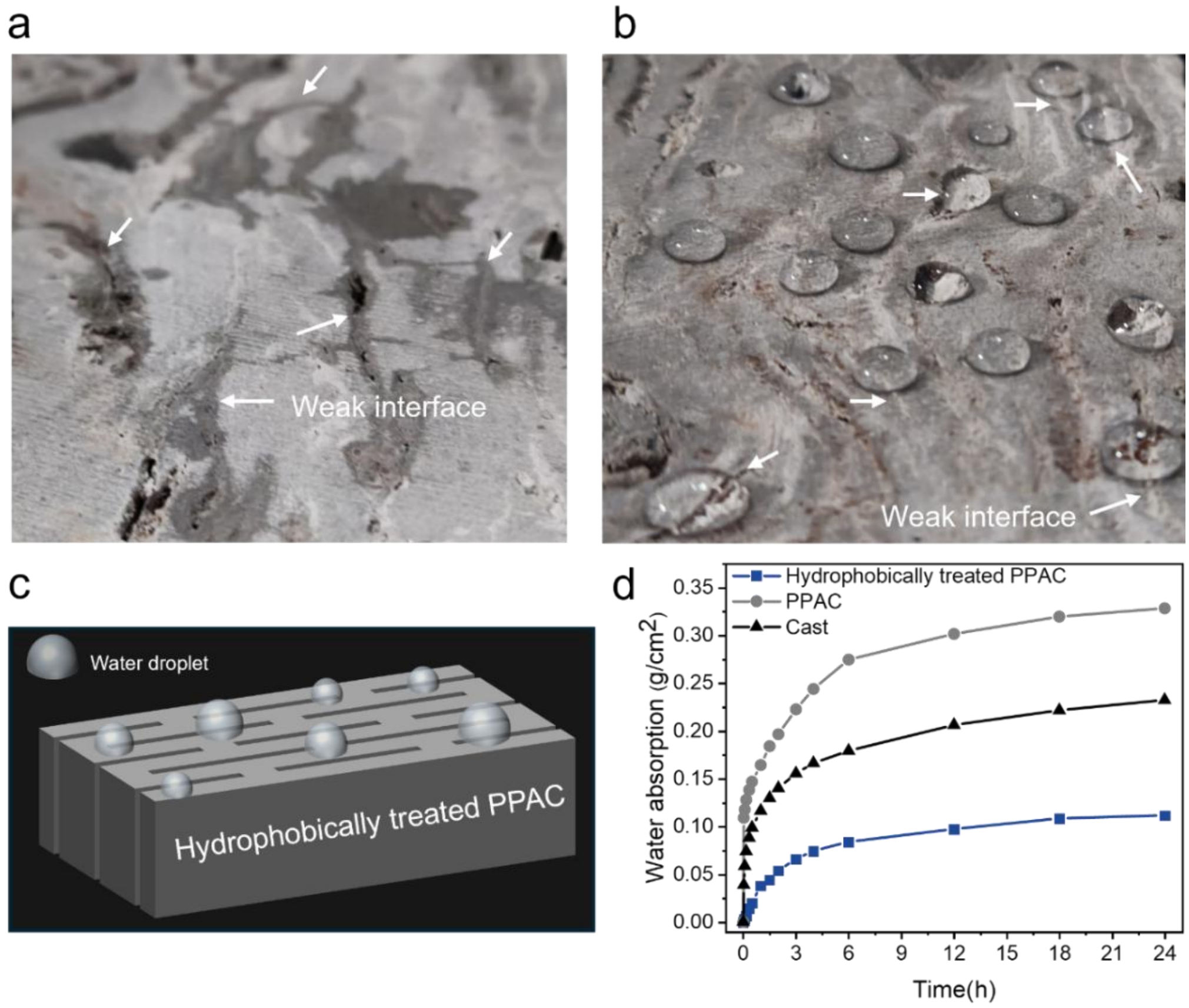

Supplementary Fig. 33. Preventing water ingress into PPAC through surface hydrophobic treatment. a Water droplet absorption on the PPAC surface without hydrophobic treatment. **b** hydrophobically treated PPAC surface. **c** Schematic illustration of a water droplet on the hydrophobically treated PPAC surface. **d** Water absorption test results.

Supplementary discussion 4. Evaluation of processing simplicity and Industrial feasibility

Reproducibility: The PPAC fabrication process proposed in this study mainly consists of four steps: preparation of the sodium alginate solution, cement paste mixing, rolling–folding, and curing. The materials used in PPAC are all conventional constituents in cement-based materials and commercially available industrial products. No complex pre-treatment of raw materials is required during processing, which ensures that the material composition can be readily fixed and remains stable across repeated production.

The equipment involved in this study includes a mixer for preparing the sodium alginate solution, a mixer for preparing cement paste, and a machine for the rolling–folding process, both of which are relatively low-cost. The preparation of the sodium alginate solution involves a simple powder dissolution and stirring process without the need for heating, making it readily scalable for industrial production. Similarly, cement paste mixing is a well-established and robust process, and the reproducibility can be ensured by using standardized mixing equipment and maintaining consistent mixing durations.

However, we acknowledge that the rolling-folding process presents certain challenges in terms of reproducibility and stability, but these challenges may be addressed by industrial design and optimization. In this study, due to limitations of the available equipment, several steps in the rolling process were performed manually. Extending the fabrication process in this study to an industrial-scale operational workflow could significantly improve process stability.

Overall, the PPAC fabrication process does not involve any intrinsically difficult-to-control physicochemical processes, such as freeze–thaw cycling, pore-forming, or granulation. Given that the formation of the lamellar structure in PPAC is governed by a relatively simple underlying mechanism, its fabrication is expected to benefit significantly from mechanization and automation, thereby enhancing reproducibility and controllability in large-scale production.

Dimensional Control: The dimensional control of PPAC is relatively straightforward. The thickness of PPAC can be precisely regulated by adjusting the distance between the rollers and the base plate in the rolling equipment. For the fabrication of larger structural elements, such as wall panels, slabs or beams, this can be achieved either by employing larger-scale rolling equipment or by adopting rolling-folding equipment with larger size or using assembly strategy at the plastic stage of PPAC. Given the inherently straightforward underlying mechanisms of the PPAC fabrication process, the development of more precise and controllable large-scale manufacturing approaches in industrial settings is expected to be feasible, primarily relying on equipment advancement and process standardization.

Potential Industrial production line: Based on the PPAC fabrication process, industrial production line can be speculated with the aid of automatic and robotic equipment. For example, during the rolling-folding process, the roller height and motion can be precisely and automatically controlled by motors. The spraying of sodium alginate (SA) solution on material surface can be automatically accomplished using a robotic arm. Subsequently, the material can be subjected to cutting and folding operations using robotic arms or other automated mechanisms. Then, the material can be returned to the rolling stage, where the aforementioned sequence—SA spraying, cutting, and folding—can be repeated. By controlling the number of production cycles, plastic PPAC with a designed number of lamellar interfaces can be fabricated.

Supplementary method 1. Calculation process of mechanical parameters

Modulus of Rupture (MOR): The MOR was determined from the 3-point bending test results of unnotched specimens. According to ASTM C293M-16[7], the calculation equation is:

$$MOR = \frac{3P_{max}L}{2WD^2} \tag{1}$$

Where *L*, *W* and *D* are the span length, width and depth of the specimen, respectively. P_{max} is the peak load of the load-midspan displacement curve.

Flexural Toughness: The flexural toughness ($KJ \cdot m^{-3}$) generally represents the energy absorbed by the material per unit volume of the bent region. According to literature[16, 17], the flexural toughness can be calculated by the area enclosed by the flexural stress-strain curve of the material during bending. The strain here is actually equivalent strain for the 3-point bending test specimen, which is calculated by:

$$\varepsilon_e = \frac{6\delta D}{L^2} \tag{2}$$

Where ε_e is the equivalent strain, δ is the midspan deflection, *D* is the depth of the specimen.

In 3-point bending, the flexural stress can be calculated by:

$$\sigma = \frac{3pL}{2WD^2} \tag{3}$$

Where *p* is the crosshead load. Then, the flexural toughness is calculated by:

$$Flexural\ Toughness = \sum_{n=0}^{Failure} \sigma_n \times \varepsilon_{e_n} \tag{4}$$

Fracture Toughness: The fracture toughness was determined from the single-edge notch bend (SENB) test results according to ASTM E1820-20b[8]. The crack initiation fracture toughness, K_{IC}, is calculated by:

$$K_{IC} = \frac{P_c L}{WD^{1.5}} * f(\frac{a_0}{D}) \tag{5}$$

Where *L*, *W* and *D* are the span length, width and depth of the specimen, respectively. a_0 is the notch length, which is 3mm in this study. P_C is the load corresponding to the crack initiation. Digital Image Correlation (DIC) was used to monitor the occurrence of crack initiation, and the corresponding load at that moment was taken as the P_C.

The function $f\left(\frac{a_0}{D}\right)$ is defined by the geometrical parameters and the notch length of the SENB specimen, and is calculated by:

$$f\left(\frac{a_0}{D}\right) = \frac{3\left(\frac{a_0}{D}\right)^{\frac{1}{2}}\left(1.99 - (\frac{a_0}{D})(1-\frac{a_0}{D})\left[2.15 - 3.93\left(\frac{a_0}{D}\right) + 2.7(\frac{a_0}{D})^2\right]\right)}{2\left(1+\frac{2a_0}{D}\right)\left(1-\frac{a_0}{D}\right)^{\frac{3}{2}}} \tag{6}$$

In this study, the $f\left(\frac{a_0}{D}\right)$ is equal to 1.442438249.

The Maximum fracture toughness, K_{JC}, was determined by:

$$K_{JC} = \left[(J_{el} + J_{pl})E'\right]^{\frac{1}{2}} \tag{7}$$

Where J_{el} is the elastic component of J-integral, J_{pl} is the plastic component of J-integral, E' is the plain strain Young's modulus.
According to Refs.[6, 18], E' value for plane strain was calculated by employing the rule of mixture for a binary composite system. In this study, the puff pastry architected cementitious material (PPAC) can be regarded as a binary system, in which the two phases are the cement matrix and air. The equation is:

$$E' = \frac{E'_{cement}D_{cement} + E'_{air}D_{air}}{D_{cement} + D_{air}} \tag{8}$$

Where E'_{cement} and E'_{air} are the plane strain Young's modulus of cement paste matrix and air. D_{cement} and D_{air} are the total cumulative thickness of cement matrix phase and air cavity phase. The E'_{cement} is given by:

$$E'_{cement} = E_{cement}(1 - v^2) \tag{9}$$

Where E_{cement} is the Young's modulus of hardened cement paste, v is the Poisson's ratio. In this study, the E'_{cement} is 27 GPa [6, 19-22] and The E'_{air} is 0. The E' is calculated according to the density ratio of PPAC/Cast. The E' of PPAC-Fold 3, PPAC-Fold 5 and PPAC-Fold7 are 26.5, 24.2 and 22.3 GPa, respectively.
The elastic component of J-integral, J_{el} was based on linear elastic fracture mechanics, which is calculated by:

$$J_{el} = \frac{K_{IC}^2}{E'} \tag{10}$$

The plastic component of J-integral, J_{pl} was calculated by:

$$J_{pl} = \frac{\eta_{pl}A_{pl}}{W(D - a_0)} \tag{11}$$

Where η_{pl} is the dimensionless function of geometry and is equal to 1.9 according to ASTM E1820-20b. A_{pl} is the area under the load-midspan displacement curve beyond the load corresponding to the initiation of the crack (P_C):

$$A_{pl} = \sum_{n=P_C}^{Failure} P_n \times \delta_n \tag{12}$$

The R-curve can be obtained by plotting K_J as a function of crack extension, Δa, which can be calculated by:

$$\Delta a = a_n - a_0 \tag{13}$$

$$a_n = a_{n-1} + \frac{D - a_n}{2}\frac{C_n - C_{n-1}}{C_n} \tag{14}$$

Where C_n is the instantaneous compliance of the specimen, which is defined as:

$$C_n = \frac{u_n}{P_n} \tag{15}$$

Where u_n is the mid-span displacement and P_n is the corresponding load value.
According to ASTM E1820-20b[8], the maximum crack extension capacity for the specimen is defined as:

$$a_{max} = 0.25(D - a_0) \tag{16}$$

The K_J value in the R-curve corresponding to a_{max} was used for calculating the fracture toughness (K_{JC}).

Supplementary References

[1] Y. Chen, K. Xia, Z. Jia, Y. Gao, Z. Zhang, Y. Zhang, Extending applicability of 3D-printable geopolymer to large-scale printing scenario via combination of sodium carbonate and nano-silica, Cement and Concrete Composites 145 (2024).
[2] Q. Yu, B. Zhu, X. Li, L. Meng, J. Cai, Y. Zhang, J. Pan, Investigation of the rheological and mechanical properties of 3D printed eco-friendly concrete with steel slag, Journal of Building Engineering 72 (2023).
[3] D. Kühbeck, J. Mayr, M. Häring, M. Hofmann, F. Quignard, D. Díaz Díaz, Evaluation of the nitroaldol reaction in the presence of metal ion-crosslinked alginates, New Journal of Chemistry 39(3) (2015) 2306-2315.
[4] M.J. Costa, A.M. Marques, L.M. Pastrana, J.A. Teixeira, S.M. Sillankorva, M.A. Cerqueira, Physicochemical properties of alginate-based films: Effect of ionic crosslinking and mannuronic and guluronic acid ratio, Food Hydrocolloids 81 (2018) 442-448.
[5] Z.B. Zhang, H.L. Gao, S.M. Chen, S.H. Yu, Anisotropically Thermal-Protective Porous Ceramics Enabled by Nacre-Like Framework, Adv Mater (2025) e2506308.
[6] S. Gupta, H.S. Esmaeeli, R. Moini, Tough and Ductile Architected Nacre-Like Cementitious Composites, Advanced Functional Materials (2024).
[7] ASTM C293/C293M-16: Standard Test Method for Flexural Strength of Concrete (Using Simple Beam with Center-Point Loading), ASTM International, West Conshohocken, PA, 2016.
[8] ASTM E1820-20b: Standard Test Method for Measurement of Fracture Toughness, ASTM International, West Conshohocken, PA, 2020.
[9] A.E. Torres-García, R.O. Agbaoye, L. Carlosena, G. Goracci, C. Lezaun, J.S. Dolado, M. Beruete, Towards cooling concrete: Evaluation of cement and cement composites under realistic climatic conditions, Applied Thermal Engineering 265 (2025).
[10] D. Liu, J.C.O. Zepper, D. Fan, Q. Yu, Photocatalytic cement-based passive cooling composites: The synergy between radiative cooling and self-cleaning mechanisms, Journal of Building Engineering 85 (2024).
[11] A. Urzędowski, D. Wójcicka-Migasiuk, B. Buraczyńska, Visual Effects of Surface Emissivity in Thermal Imaging, Advances in Science and Technology Research Journal 14(2) (2020) 215-222.
[12] Y. Chen, Y. Zhang, Y. Xie, Z. Zhang, N. Banthia, Unraveling pore structure alternations in 3D-printed geopolymer concrete and corresponding impacts on macro-properties, Additive Manufacturing 59 (2022).
[13] N.S. Martys, C.F. Ferraris, Capillary transport in mortars and concrete, Cement Concrete Res 27(5) (1997) 747-760.
[14] L. Basheer, D.J. Cleland, A.E. Long, Protection provided by surface treatments against chloride induced corrosion, Materials and Structures 31(7) (1998) 459-464.
[15] E. Mora, G. González, P. Romero, E. Castellón, Control of water absorption in concrete materials by modification with hybrid hydrophobic silica particles, Construction and Building Materials 221 (2019) 210-218.
[16] Y. Chen, Y. Zheng, Y. Zhou, W. Zhang, W. Li, W. She, J. Liu, C. Miao, Multi-layered cement-hydrogel composite with high toughness, low thermal conductivity, and self-healing capability, Nature Communications 14(1) (2023).
[17] J. Chen, Z. Liu, S. Zhang, S. Hu, F. Wang, Energy-Efficient Fabrication of Biomimetic Materials for Sustainable Infrastructure Applications, Advanced Science (2025).

[18] E. Sparks, Advances in Military Textiles and Personal Equipment, ELSEVIER2012.
[19] C.J. Haecker, E.J. Garboczi, J.W. Bullard, R.B. Bohn, Z. Sun, S.P. Shah, T. Voigt, Modeling the linear elastic properties of Portland cement paste, Cement Concrete Res 35(10) (2005) 1948-1960.
[20] D. Aniszewska, M. Rybaczuk, Predicting the Young's Modulus of Concrete Using a Particle-Based Movable Cellular Automata Method, Applied Sciences 15(9) (2025).
[21] R. Narayan Swamy, Dynamic Poisson's ratio of portland cement paste, mortar and concrete, Cement Concrete Res 1(5) (1971) 559-583.
[22] L.J. Parrott, Lateral strains in hardened cement paste under short- and long-term loading, Magazine of Concrete Research 26(89) (1974) 198-202.
