# Supplementary material for: Tough, thermal insulating and scalable puff pastry-inspired architected cementitious material via simple rolling-folding
Source: Nat Commun. 2026 Aug 27;17:10244. doi: 10.1038/s41467-026-77120-z (PMC13616957; doi:10.1038/s41467-026-77120-z)
Supplement: Supplementary file 2 [file 41467_2026_77120_MOESM2_ESM.pdf]

Description of Additional Supplementary Files:

Supplementary Movie 1. Ductile fracture process of PPAC with different number of rolling-folding cycles;

Supplementary Movie 2. Mechanical interlocking effect of PPAC after crack penetration;

Supplementary Movie 3. Simulated heat transfer process within Cast and PPAC specimens.
