# Supplementary material for: Tough, thermal insulating and scalable puff pastry-inspired architected cementitious material via simple rolling-folding
Source: Nat Commun. 2026 Aug 27;17:10244. doi: 10.1038/s41467-026-77120-z (PMC13616957; doi:10.1038/s41467-026-77120-z)
Supplement: Supplementary file 3 [file 41467_2026_77120_MOESM6_ESM.pdf]

nature portfolio

Peer Review File

Tough, thermal insulating and scalable puff pastry-inspired architected cementitious material via simple rolling-folding

Corresponding Author: Professor Yamei Zhang

This file contains all reviewer reports in order by version, followed by all author rebuttals in order by version.

Version 0:

Reviewer comments:

Reviewer #1

(Remarks to the Author)
The most noteworthy results of this manuscript are the compelling processing–structure–property relationships that are demonstrated. The baking-inspired processing technique produces a well-defined lamellar microstructure within the cement paste, which imparts exceptional mechanical and thermal properties compared to cast cement samples. A hydrated sodium alginate (SA) layer is used to maintain separation between layers, creating a low-density interlayer.

To my knowledge, this is an original and interesting concept. Rolling cement paste without inducing significant cracking or mechanical degradation is likely nontrivial, which further highlights the novelty of the approach. It does raise the question of whether an additional control—such as rolled cement layers without the SA interlayer—could help isolate and quantify the contribution of the biopolymer. While the work clearly fits within the scope of traditional civil engineering and concrete materials science, it also has strong relevance from a processing and materials science perspective. In particular, there appear to be opportunities to strengthen connections with the polymer science community, as discussed below.

Overall, the majority of the conclusions and claims are supported by the presented data. However, there are several areas where explanations could be clarified or claims strengthened through additional discussion or measurements:

1. Role and behavior of the SA interlayer.
The contributions of the SA layer are not described in sufficient detail. While I recognize this is not the main focus of the study, this represents a missed opportunity to engage more deeply with the polymer science community. This issue is most apparent in the schematic of the “cracked” polymer film in Fig. 1b, which appears unrealistic from a polymer mechanics perspective. If the SA layer is well hydrated and the system is not excessively dry, the compressed film may stretch, deform, or locally rupture—but it may also remain intact if the SA chains can relax under applied stress. Clarifying this behavior requires a better understanding of the structure–property relationships of the SA layer itself. For example, is the SA a crosslinked hydrogel with a storage modulus exceeding its loss modulus? If so, and if deformation occurs sufficiently rapidly, rupture could occur—though it would likely produce rounded voids rather than a brittle, map-cracked morphology. If not, viscous flow would be expected and rupture would be unlikely.

2. Surface crosslinking of SA (line 95).
The authors state that SA crosslinks on the surface. Are there any observations or measurements to support this claim? It seems plausible that the SA layer could bond to both the lower and upper cement paste layers during processing, but this mechanism is not clearly demonstrated.

3. Water transport from the SA layer (line 194).
The phrase “SA hydrogel undergoes water separation” is unclear. Terms such as “dehydration” or “fluid desorption from the SA layer” would be more precise. This discussion would also benefit from referencing the concept of hydrogel-based internal curing of concrete. In this established approach, water diffuses from hydrogel inclusions into the cement matrix during early hydration as internal relative humidity decreases, driven by osmotic pressure gradients. This controlled water release mitigates self-desiccation and shrinkage, reduces microcracking, and improves mechanical properties—particularly in low water-to-cement ratio systems like the w/c = 0.3 mixture used here. It is therefore plausible that the SA layers act as internal curing agents over time, which may help explain the excellent mechanical performance of the PPAC. Definitively testing this hypothesis would require a control in which the polymer layer neither swells nor releases water (e.g., an inert

plastic interlayer), which may be challenging and is not necessarily required for this study.

Relevant references from the hydrogel-based internal curing literature include:
https://www.sciencedirect.com/science/article/abs/pii/S0008884621002970
https://link.springer.com/article/10.1617/s11527-014-0308-5
https://www.sciencedirect.com/science/article/abs/pii/S0950061816300927
https://link.springer.com/article/10.1617/s11527-018-1241-9

There do not appear to be any major issues with data analysis, interpretation, or overall conclusions. The methodology is sound, aligns with standards in the civil engineering and concrete materials science community, and is described with sufficient detail to allow reproducibility.
Several minor points could further improve clarity and presentation:
1. The cartoons in Fig. 5d are visually appealing but not essential.
2. Line 227: correct material names to polypropylene and polycarbonate.
3. Line 56: consider noting that similar layered architectures can also be achieved via 3D printing, referencing existing citations (e.g., Ref. 24).
4. The manuscript is generally well written, but minor grammatical and word-choice edits would further improve clarity.

Reviewer #2

(Remarks to the Author)
The current work introduces “puff pastry” architected material with improved mechanical and thermal insulating properties. This work is nicely executed and contributes to the field of tough and ductile architected cementitious materials. The mechanical testing (MOR, ductility, and fracture toughness) is comprehensive, and the results are effectively supported by microstructural analysis. The use of Micro-CT and SEM to connect toughening mechanisms (such as crack deflection and interlocking) to fracture behavior is nicely performed. The microstructural analysis and the corresponding discussion regarding the relative length of the weakness phase compared to the thickness of the solid phase explain the toughening mechanisms in specimens with different folds (3, 5, & 7 folds).

The study also demonstrates additional applications through thermal insulation characterization and connects these results to microstructural features. The fabrication process based on rolling during the plastic stage is new and interesting. Furthermore, rheological analysis of cement paste and investigation of sodium alginate hydrogel interactions with cement paste are valuable additions. However, there are the following questions that should be addressed. The work shows strong potential; however, I recommend a major revision to adequately address the following comments.

1. Since this study uses hydrogels, it would be beneficial to include a discussion of recent hydrogel-cementitious composite studies, such as Wang, Han, et al., npj Materials Sustainability 3.1 (2025): 28 and Wang, Han, et al., Cement and Concrete Research 195 (2025): 107926.

2. Number of folds appears to significantly influence performance. Fold-7 shows better performance than Fold-3 and Fold-5, while the difference between Fold-3 and Fold-5 in terms of specific KJC and MOR is not significant (Fold-5 even shows slightly lower averages). Since Fold-7 demonstrates clear improvement, it would be useful to comment on whether increasing the number of folds further could continue improving fracture toughness, or whether fabrication limits exist due to material properties. Microstructural observations suggest that higher folds reduce the relative thickness of the solid phase and enhance interlocking, which may further improve fracture toughness in higher folds (8, 9, 10, and so on) specimens.

3. The size effect is important in cementitious materials. Although the total number of folds is increased while keeping specimen size constant, fracture testing is performed only on samples with 40 mm thickness. The large-scale PPAC plate (40 cm × 40 cm × 4 cm) presented in the Supplementary Information is a good addition, but discussion of size effects on fracture properties and strength would strengthen the study.

4. How many repetitions were performed for each test? Were statistical significance tests performed for the results shown in Figure 2? For example, Line 122 states that a parameter “was not significantly affected.” Please clarify whether this refers to statistical significance, and please include tests if they were not conducted.

5. Please clarify how flexural toughness is calculated (Line 132). Also, please justify the use of a span-to-depth ratio of 2.5, as flexural tests and SENB typically use ratios of 3 or higher. With a lower span-to-depth ratio, the flexural test becomes shear dominated.

6. In Fig. 2j (R-curve), crack extension may decrease locally due to compliance changes, which is understandable. However, KJC also decreases at certain points for Fold-7 in the R-curve. According to Supplementary Equation 8, Apl increases with crosshead displacement while other parameters (W, D, a0) remain constant, so Jpl and KJC should increase. Please review the plot or clarify whether instantaneous crack length is used in Equation 8.

7. How would the samples perform when loaded in the other two perpendicular orientations? A brief discussion would be helpful.

8. Lines 144 and 146: Please clarify the meaning of “hierarchical failure”. Typically, hierarchical cracks refer to multi-scale

fracture networks composed of interconnected cracks of different lengths. The DIC results appear to show one primary deflected crack and one branch; describing this as hierarchical may be excessive.

9. Line 169 and Fig. 3b: Please specify whether the reported value corresponds to the maximum or average backside temperature.

10. In this study, the samples are fabricated using cement paste; however, the large infrastructure materials are fabricated using mortar or concrete. Concrete/mortar potentially shows complex plastic behavior due to the presence of aggregates, which may impact the fabrication process used in this study. Can you address the potential extension of the fabrication process to mortar and concrete? In addition, adding brief comments in the Conclusion section on scalability to infrastructure applications, including potential use of robotics or hybrid additive manufacturing approaches, could be beneficial.

11. The viscosity value (75000) in Supplementary Line 24 and Line 282 is missing a unit, and there is a typo in Supplementary Line 24: “power” should be “powder.” Please review the manuscript for proofreading.

Reviewer #3

(Remarks to the Author)
Reviewer’s Opinion – 673946_0_other_1_t6swm9

The manuscript presents a novel and conceptually strong approach supported by substantial experimental evidence and with clear potential impact. However, key aspects related to durability, broader mechanical characterization, scalability, and the limitations of thermal testing and modeling require deeper discussion and clarification before the work can be accepted. The study is promising and likely publishable after substantial but feasible revisions.
Recommendation: Major Revision
The manuscript presents a novel puff pastry architected cementitious material (PPAC) fabricated via a simple rolling–folding process conducted entirely under ambient conditions. Inspired by laminated pastry processing, the approach employs in situ crosslinked sodium alginate to generate discrete weak interfaces that, after fragmentation and drying, form a lamellar architecture with embedded cavities. The material exhibits markedly enhanced specific fracture toughness (≈ 90 MPa·mm^0.5/g·cm³), pronounced ductile behavior, and significantly reduced thermal conductivity (0.38 W/m·K), while maintaining comparable specific strength to cast cement paste. The concept is original, experimentally well supported, and potentially impactful for multifunctional infrastructure materials.
The experimental program is comprehensive, combining 3PB and SENB testing, R-curve analysis with DIC monitoring, X-CT and SEM/EDS characterization, flame-based thermal testing, and multiphysics heat-transfer modeling. The correlation between architecture, crack deflection, mechanical interlocking, and macroscopic toughening is convincingly demonstrated.
However, several issues require clarification. First, claims regarding scalability and infrastructure applicability would benefit from a more rigorous discussion of process reproducibility, dimensional control, and industrial feasibility. Second, mechanical characterization is limited to monotonic flexure; compressive behavior, fatigue resistance, and durability (e.g., permeability, freeze–thaw resistance, and the chemical stability of alginate films in alkaline environments) are not addressed. Third, the flame test is illustrative but not standardized for structural fire resistance, and potential thermal anisotropy is not quantified. Finally, the thermal model relies on geometric simplifications whose limitations should be more explicitly acknowledged.
Overall, the study introduces a compelling and energy-efficient architectural strategy for cementitious materials, achieving substantial improvements in toughness and thermal insulation. With a strengthened discussion of durability, scalability, and testing limitations, the manuscript would represent a significant contribution to architected cement-based materials.

Version 1:

Reviewer comments:

Reviewer #1

(Remarks to the Author)
I am satisfied with the changes the authors have made in the revised document. All my comments and questions were addressed. The changes are substantial and add value and greater insight. This is a high quality and interesting study, now worthy of publication in this journal.

Reviewer #2

(Remarks to the Author)
The authors have addressed my comments and incorporated the corresponding revisions. Therefore, I recommend publication of the manuscript, subject to the following minor comment, which should be addressed:

- Please include the units of the reported properties in Supplementary Tables 13 and 14. In addition, please clarify whether the significance analysis was performed for specific KIC and KJC values or for KIC and KJC.

Reviewer #3

(Remarks to the Author)
Reviewer's Opinion – 673946_0_other_1_t6swm9

The manuscript presents a novel and conceptually strong approach supported by substantial experimental evidence and with clear potential impact. However, key aspects related to durability, broader mechanical characterization, scalability, and the limitations of thermal testing and modeling require deeper discussion and clarification before the work can be accepted. The study is promising and likely publishable after substantial but feasible revisions.
Recommendation: Major Revision
The manuscript presents a novel puff pastry architected cementitious material (PPAC) fabricated via a simple rolling–folding process conducted entirely under ambient conditions. Inspired by laminated pastry processing, the approach employs in situ crosslinked sodium alginate to generate discrete weak interfaces that, after fragmentation and drying, form a lamellar architecture with embedded cavities. The material exhibits markedly enhanced specific fracture toughness (≈ 90 MPa·mm^0.5/g·cm³), pronounced ductile behavior, and significantly reduced thermal conductivity (0.38 W/m·K), while maintaining comparable specific strength to cast cement paste. The concept is original, experimentally well supported, and potentially impactful for multifunctional infrastructure materials.
The experimental program is comprehensive, combining 3PB and SENB testing, R-curve analysis with DIC monitoring, X-CT and SEM/EDS characterization, flame-based thermal testing, and multiphysics heat-transfer modeling. The correlation between architecture, crack deflection, mechanical interlocking, and macroscopic toughening is convincingly demonstrated. However, several issues require clarification. First, claims regarding scalability and infrastructure applicability would benefit from a more rigorous discussion of process reproducibility, dimensional control, and industrial feasibility. Second, mechanical characterization is limited to monotonic flexure; compressive behavior, fatigue resistance, and durability (e.g., permeability, freeze–thaw resistance, and the chemical stability of alginate films in alkaline environments) are not addressed. Third, the flame test is illustrative but not standardized for structural fire resistance, and potential thermal anisotropy is not quantified. Finally, the thermal model relies on geometric simplifications whose limitations should be more explicitly acknowledged.
Overall, the study introduces a compelling and energy-efficient architectural strategy for cementitious materials, achieving substantial improvements in toughness and thermal insulation. With a strengthened discussion of durability, scalability, and testing limitations, the manuscript would represent a significant contribution to architected cement-based materials.

Open Access This Peer Review File is licensed under a Creative Commons Attribution 4.0 International License, which permits use, sharing, adaptation, distribution and reproduction in any medium or format, as long as you give appropriate credit to the original author(s) and the source, provide a link to the Creative Commons license, and indicate if changes were made.
In cases where reviewers are anonymous, credit should be given to 'Anonymous Referee' and the source.
The images or other third party material in this Peer Review File are included in the article's Creative Commons license, unless indicated otherwise in a credit line to the material. If material is not included in the article's Creative Commons license and your intended use is not permitted by statutory regulation or exceeds the permitted use, you will need to obtain permission directly from the copyright holder.

To view a copy of this license, visit https://creativecommons.org/licenses/by/4.0/

Response to reviewers

Manuscript title: Tough, thermal insulating and scalable puff pastry architected cementitious material via simple rolling-folding

Manuscript Number: NCOMMS-25-92106

Authors: Kailun Xia, Yuning Chen, Xingzi Liu, Siyuan Qiu, Qian Lin, Yi Tang, Tiantian Xiang, Sule Chen, Enlai Dong, Zijian Jia, Wei Wang, Yu Chen, Yamei Zhang*

Dear Editor and reviewers:

We sincerely appreciate your valuable time and effort for carefully reviewing this manuscript. We have conscientiously considered all the comments and made corresponding modifications and revisions. We believe these comments can help us improve our manuscript a lot. The reviewers' comments are listed point by point with corresponding responses. For the convenience of reviewers and editor, the corresponding modifications have been appended beneath each response, thus you can directly see these modifications. For your convenience. **Please Note:** For the convenience of the editor and reviewers, and to accommodate potential differences in review preferences, we have provided both a **marked-up manuscript with tracked changes** and a **clean version**. **All line numbers and references mentioned in the responses below correspond to the clean version of the manuscript.**

We are looking forward to your kind consideration of the suitability for accepting this manuscript after accomplishing the revision. If there are any further questions, please let us know at your convenience.

Best regards

Yamei Zhang,
Corresponding author, on behalf of all the authors

Reviewer #1

The most noteworthy results of this manuscript are the compelling processing–structure–property relationships that are demonstrated. The baking-inspired processing technique produces a well-defined lamellar microstructure within the cement paste, which imparts exceptional mechanical and thermal properties compared to cast cement samples. A hydrated sodium alginate (SA) layer is used to maintain separation between layers, creating a low-density interlayer. To my knowledge, this is an original and interesting concept. Rolling cement paste without inducing significant cracking or mechanical degradation is likely nontrivial, which further highlights the novelty of the approach. It does raise the question of whether an additional control—such as rolled cement layers without the SA interlayer—could help isolate and quantify the contribution of the biopolymer. While the work clearly fits within the scope of traditional civil engineering and concrete materials science, it also has strong relevance from a processing and materials science perspective. In particular, there appear to be opportunities to strengthen connections with the polymer science community, as discussed below.

Response:

Thank you very much for your positive comments on our work. Your recognition has given us great confidence to further improve this study. We have carefully reviewed all of your comments and found them to be highly valuable and constructive. We have revised the manuscript thoroughly in response to each of your points, as detailed in the responses below. We hope that the revised version has been significantly improved and meets your expectations. We sincerely appreciate your help.

In addition, we appreciate the reviewer's insightful suggestion to include an additional control group consisting of rolled–folded cement without SA. We agree that such a comparison is valuable for clarifying the role of SA, and we have therefore conducted supplementary experiments accordingly. The results indicate that, in the absence of SA, the rolling–folding process does not introduce noticeable changes in the mechanical performance of the cement paste. This is reasonably expected, as the duration of the rolling–folding process is relatively short, and the cement layers tend to readily rebind under the applied pressure during rolling. Given that this control experiment mainly serves to support the mechanistic role of SA rather than providing additional standalone insights, we are uncertain whether it is appropriate to include it in the main text. Therefore, we have presented these results in the **Supplementary Fig. 10 and Supplementary Table 1** for completeness. This result clearly confirms the critical role of the weak interfaces induced by SA in governing the performance of PPAC. Correspondingly, we have added a statement in **lines 124–125, Page 5** of the **main text** to further emphasize this point. We thank you for your valuable suggestion and hope that this modification addresses your concern.

Relevant revised content

Supplementary Fig. 10 and Supplementary Table 1

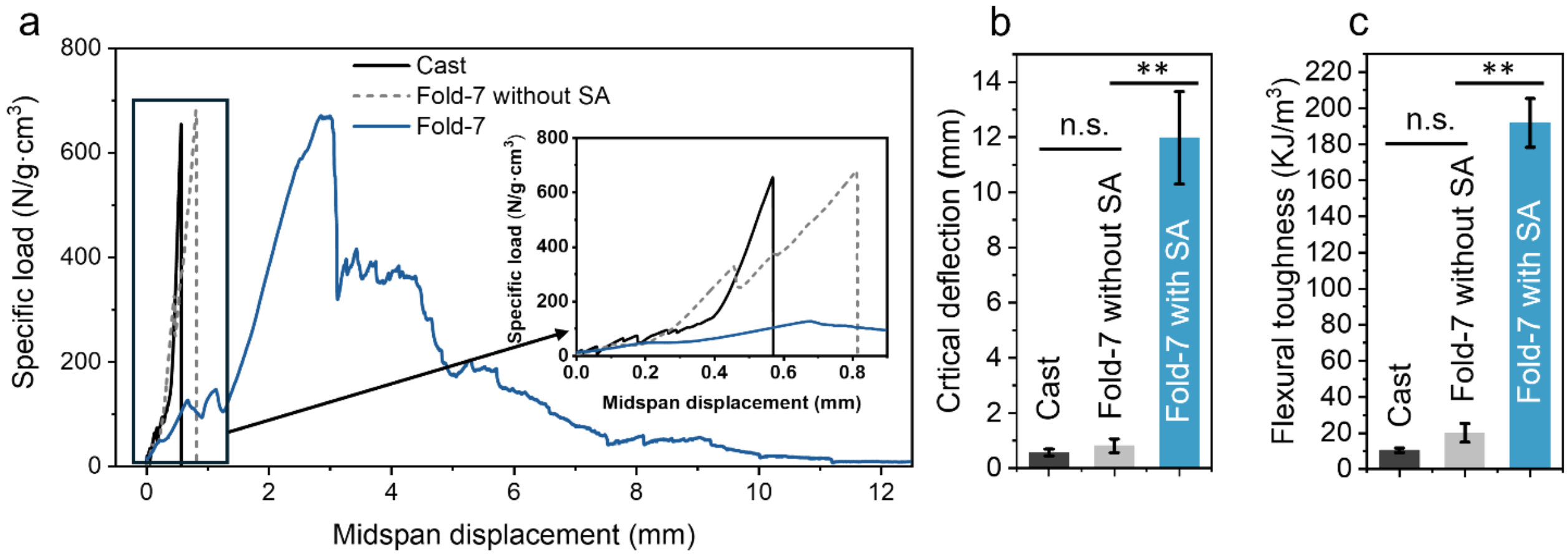

Supplementary Fig. 10. Mechanical properties of PPAC without incorporation of SA. The preparation procedure of Fold-7 without SA is essentially identical to that of the PPAC Fold-7 described in the main text, except that the step of applying the SA solution prior to each rolling cycle is omitted. **a** 3-point bending (3PB) test curves. **b-c** Comparison on critical deflection and flexural toughness between Cast, Fold-7 without SA and Fold-7 with SA. These results show no statistically significant difference between the cast and Fold-7 without SA in both critical deflection and flexural toughness. Meanwhile, critical deflection and flexural toughness of Fold-7 without SA are significantly lower than Fold-7 with SA. The significance analysis results are listed in **Supplementary Table 1.**

Supplementary Table 1. Data set and t-test significance analysis results of the mechanical properties in Supplementary Fig. 10

Sub Table a. Data set (mean±SD).

Properties	Cast	Fold-7 without SA	Fold-7	Size (n)
Critical deflection	0.57±0.12	0.81±0.25	11.97±1.68	3
Flexural toughness	10.44±1.28	20.16±5.23	191.84±13.52	3

Sub Table b. Significance analysis on critical deflection.

Comparison	Mean difference	T-value	df	p-value	Significance
Fold-7 without SA vs Cast	0.24	1.50	3	0.23	n.s.
Fold-7 vs Fold-7 without SA	11.16	11.32	2	0.007	**

Sub Table c. Significance analysis on Flexural toughness.

Properties	Mean difference	T-value	df	p-value	Significance
Fold-7 without SA vs Cast	9.72	3.13	2	0.089	n.s.
Fold-7 vs Fold-7 without SA	171.68	19.20	2	0.003	**

* ($p<0.05$) indicates a significant difference; ** ($p<0.01$) indicates a highly significant difference; n.s. means no significant difference.

lines 125–126, Page 5, Main Text-Clean Version

"As rolling–folding without SA does not significantly alter the mechanical response (**Supplementary Fig. 10**), these features are mechanistically attributed to the weak interfaces introduced by the crosslinked alginate-based hydrogel."

Overall, the majority of the conclusions and claims are supported by the presented data. However, there are several areas where explanations could be clarified or claims strengthened through additional discussion or measurements:

1. Role and behavior of the SA interlayer.

The contributions of the SA layer are not described in sufficient detail. While I recognize this is not the main focus of the study, this represents a missed opportunity to engage more deeply with the polymer science community. This issue is most apparent in the schematic of the "cracked" polymer film in Fig. 1b, which appears unrealistic from a polymer mechanics perspective. If the SA layer is well hydrated and the system is not excessively dry, the compressed film may stretch, deform, or locally rupture—but it may also remain intact if the SA chains can relax under applied stress. Clarifying this behavior requires a better understanding of the structure–property relationships of the SA layer itself. For example, is the SA a crosslinked hydrogel with a storage modulus exceeding its loss modulus? If so, and if deformation occurs sufficiently rapidly, rupture could occur—though it would likely produce rounded voids rather than a brittle, map-cracked morphology. If not, viscous flow would be expected and rupture would be unlikely.

Response:

Thank you very much for your highly valuable comments. In this study, the key role of SA lies in its crosslinking with calcium ions in the cement paste to form an alginate-based hydrogel with an isolating effect, thereby separating adjacent cementitious layers and creating weak interfaces. We have provided strong evidence supporting this mechanism in **Supplementary Figs. 3–5**, and the underlying mechanism has also been well established in our previous studies [1]. A brief description of this behavior, together with the relevant references, has been added in **Lines 99~106 of the main text**.

Your question regarding the fracture morphology of SA is highly insightful. First, we would like to clarify that the schematic illustration shown in Fig. 1 b is not merely speculative, but was drawn based on our actual experimental observations (**Supplementary Fig. 8**, attached below for your reference).

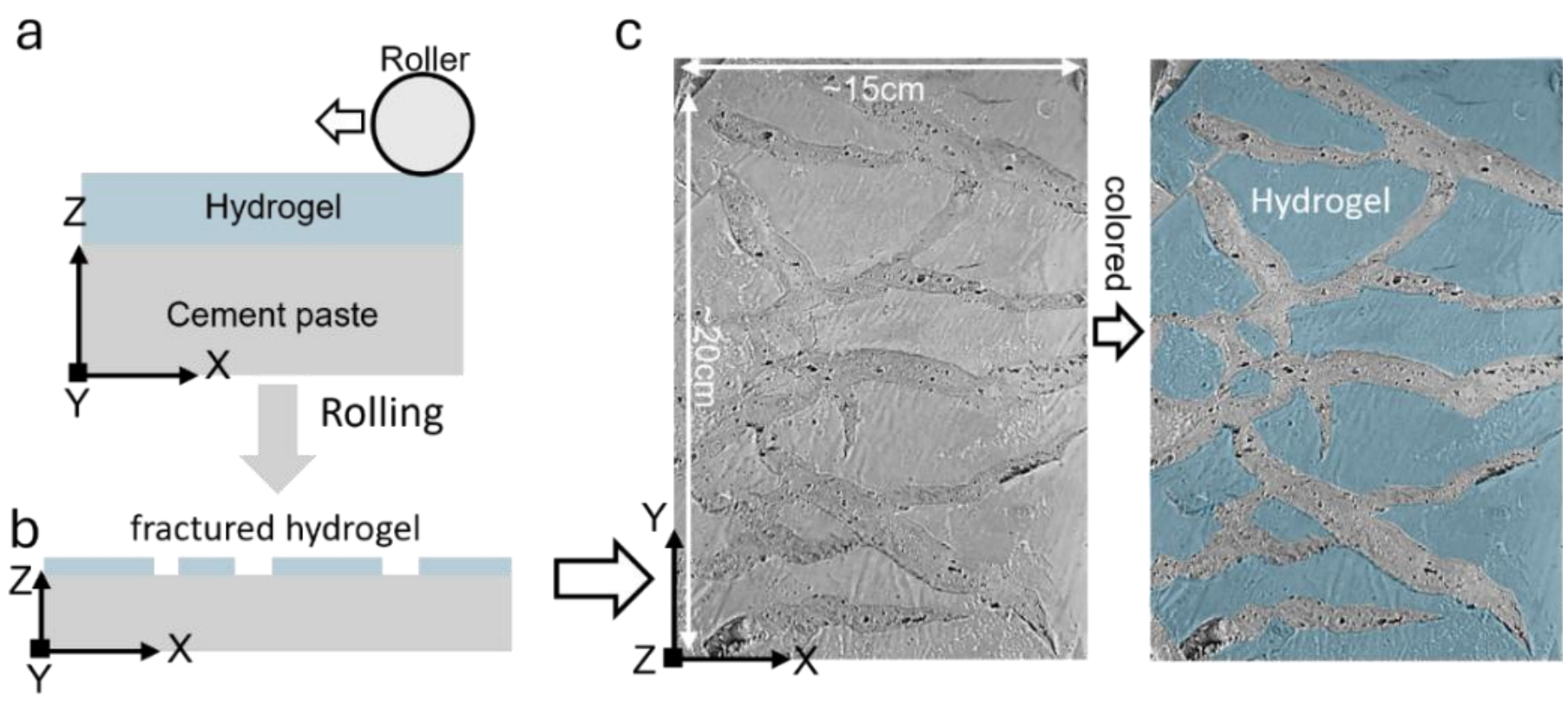

Supplementary Fig. 8. Hydrogel rupture during rolling process. a-b Schematic diagram of the hydrogel rupture during rolling. **c** Photo of ruptured hydrogel on cement paste surface after rolling procedure.

In combination with your comments, we believe that the fracture of the hydrogel can be attributed to the following factors:

(1) First, the alginate-based hydrogel formed in this study is a crosslinked hydrogel with a characteristic

"egg-box" structure, which has been extensively reported in studies [2-3]. This inherently limits its deformability. To support this, we have conducted mechanical characterization of the hydrogel in **Supplementary Figs. 6–7**.

(2) In addition, we have supplemented the water content measurement of the hydrogel in **Supplementary Fig. 3 f**, which shows that the hydrogel possesses high water content (>90%). This further increases its susceptibility to fracture during deformation under repeated mechanical rolling.

(3) Moreover, the cement paste undergoes substantial deformation during the rolling process. Under such conditions, repeated rolling induces significant in-plane elongational deformation of the cement paste surface coated with the SA solution. This elongational deformation eventually leads to in-plane fracture of the hydrogel, resulting in the map-like fracture morphology observed in our experiments.

For enhancing the clarity of relevant text, we have modified corresponding descriptions in **lines 106~114 Page 3, main text**. Following these revisions, we believe that the results currently presented in the Supplementary Information and main text can provide sufficient evidence to support the critical role of SA in the fabrication process of PPAC.

However, as you rightly pointed out, the primary focus of this study is to validate the toughness and thermal performance of PPAC. Therefore, the physicochemical properties of the hydrogel were not investigated in depth in the present work. We highly appreciate your insightful comment and fully agree that this represents a highly valuable research direction. Accordingly, we have added this point in the **"Discussion and open questions"** section and highlighted it as an important direction for future research.

References

[1] K. Xia, Y. Chen, Z. Zhang, W. Wang, Y. Chen, L. Jia, Z. Jia, S. Quan, Y. Zhang, In-situ crosslinked nano SiO2 reinforced alginate bio-textile for mitigating plastic shrinkage in 3D printed concrete, Additive Manufacturing, 2025.

[2] Q. Li, Y. Li, X. Ma, Q. Du, K. Sui, D. Wang, C. Wang, H. Li, Y. Xia, Filtration and adsorption properties of porous calcium alginate membrane for methylene blue removal from water, Chemical Engineering Journal 316 (2017) 623-630.

[3] M.J. Costa, A.M. Marques, L.M. Pastrana, J.A. Teixeira, S.M. Sillankorva, M.A. Cerqueira, Physicochemical properties of alginate-based films: Effect of ionic crosslinking and mannuronic and guluronic acid ratio, Food Hydrocolloids 81 (2018) 442-448.

Relevant revised content

lines 106~114, Page 3, Main Text-Clean Version

"Based on this, lamellar cement-based materials can be created by rolling, cutting, folding, and repeating this process with cement paste coated with the alginate-based hydrogel (Step 1~3 in **Fig. 1 b**). Due to its "egg-box" crosslinked network [66] (**Supplementary Fig. 4**) and its highly hydrated state (water content>90%, **Supplementary Fig. 3 f**), the alginate-based hydrogel generated on the surface of cement paste possess limited deformability (**Supplementary Figs. 6-7**). As a result, it can fracture with the in-plane elongational deformation of plastic cement paste during repeated rolling process (Step 3 in **Fig. 1 b**, **Supplementary Fig. 8**), creating regions that allow mechanical interlocking between adjacent cement paste layers, thereby maintaining the overall integrity of the manufactured material (Step 4 in **Fig. 1 b, Supplementary Figs. 9**)."

Supplementary Fig. 3, Supplementary Information

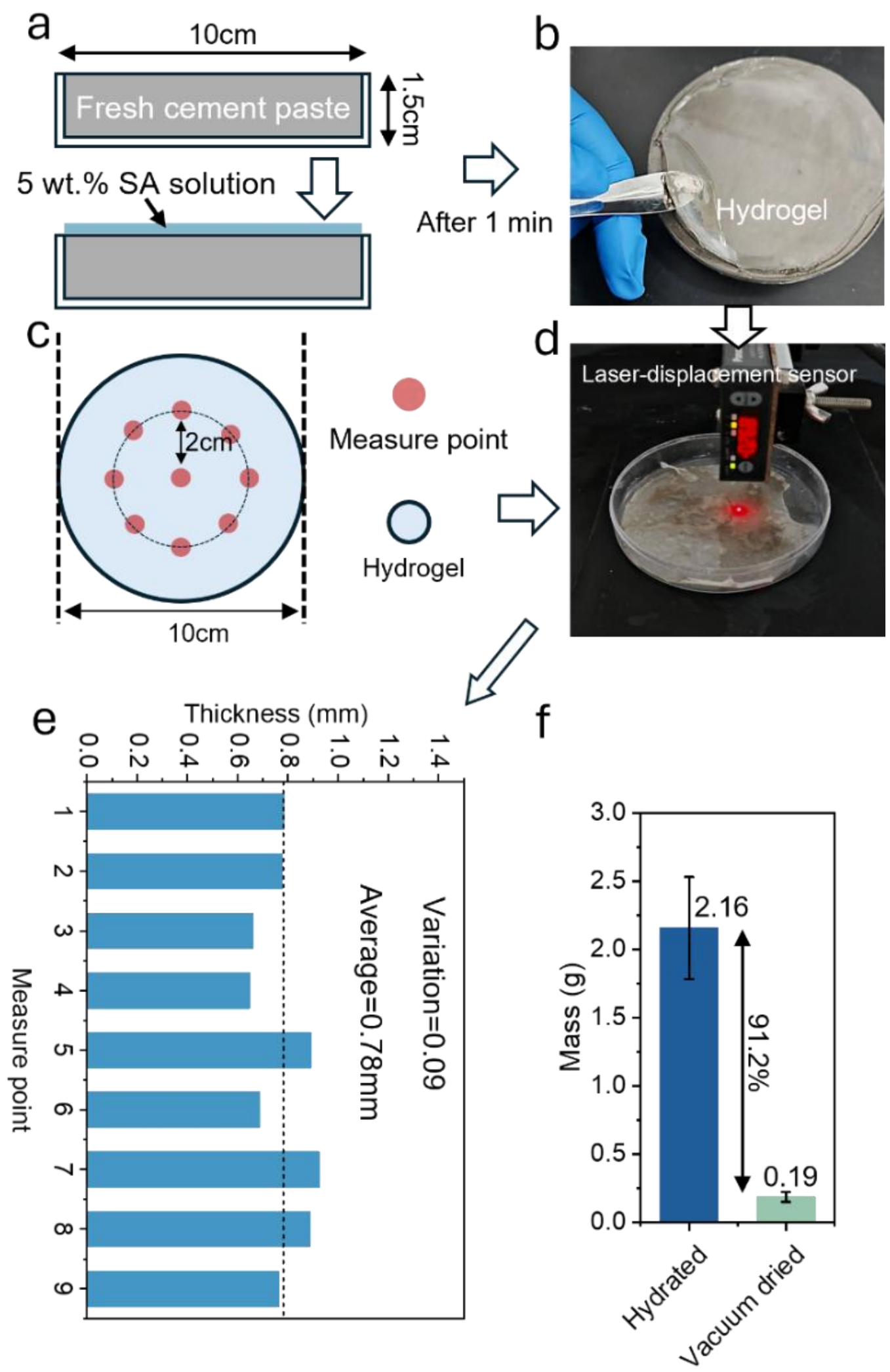

Supplementary Fig. 3. In situ generated hydrogel on fresh cement paste surface via sodium alginate crosslinking. a The fresh cement paste was cast into a circular Petri dish with a diameter of 10 cm and a height of 1.5 cm. Then, approximately 2.5 g of a 5 wt% SA solution was evenly applied to the surface of the cement paste. **b** After 1 minute, the SA solution had formed a hydrogel. **c-d** The thickness of the hydrogel was measured using a laser displacement sensor. **e** The results showed that the hydrogel had an average thickness of 0.78 mm. **f** Comparison on the mass of the generated hydrogel at the initial state (hydrated) and after vacuum dried. The result indicates that the water content of the hydrogel is about 91.2%.

Lines 99~106, Page 3, Main Text-Clean Version

"Meanwhile, sodium alginate (SA) is employed as the "butter" to prevent interlayer bonding between adjacent cement lamellar. SA is a low cost, non-toxic bio-extracted polysaccharide capable of crosslinking with di- and tri-valent metal ions, especially calcium ions, to form stable and water insoluble hydrogels [59-63]. Owing to the abundant release of calcium ions from the surface of fresh cement paste, SA can rapidly and spontaneously crosslink on the surface without the need for any additional treatment [64, 65] (Step 1 in **Fig. 1 b, supplementary Figs. 3-4**). This crosslinked hydrogel provides the basis for preventing interlayer bonding in the cement paste (**Supplementary Fig. 5**) and for creating well-ordered weak interfaces."

lines 320~324, Page 14, Discussion and Open questions, Main Text-Clean Version

"For example, while SA-crosslinked hydrogels have been shown to effectively introduce weak interfaces in cementitious materials, their physicochemical properties and the influence of their tunability on interfacial morphology require further investigation. Moreover, other materials capable of generating similar weak interfaces may exist, offering opportunities to expand the PPAC concept and potentially introduce additional functionalities."

2. Surface crosslinking of SA (line 95).

The authors state that SA crosslinks on the surface. Are there any observations or measurements to support this claim? It seems plausible that the SA layer could bond to both the lower and upper cement paste layers during processing, but this mechanism is not clearly demonstrated.

Response:

Thank you very much for your valuable comments. The crosslinking reaction between SA and calcium ions is a well-recognized phenomenon and has been widely reported in the literature, such as [1-3]. It is also well known that cement-based materials contain abundant calcium ions on their surfaces, and the formation of SA hydrogels via crosslinking on fresh cementitious surfaces has been reported previously [4]. In addition, prior to this study, our group published a work that carefully investigated and confirmed the in-situ crosslinking behavior of sodium alginate on the surface of fresh cementitious materials [5]. We briefly explained the reason for the crosslinking of SA on the surface of the cement paste in **Lines 102-106, Page 3** in the main text and have added the citation of this work to support the crosslinking behavior of SA.

Furthermore, in **Supplementary Fig. 3**, we demonstrate the process in which an SA solution was applied onto the surface of cement paste and the resulting hydrogel layer was peeled off after 1 minute (Please see the response to your previous comment above). We also conducted FTIR analysis on the dried hydrogel product, as presented in **Supplementary Fig. 4.** The results confirm that the product is indeed calcium alginate hydrogel formed by the crosslinking reaction between sodium alginate and calcium ions.

At the same time, your comment made us realize that our study may lack a direct visual demonstration showing that the hydrogel layer effectively prevents bonding between two layers of cement paste. To address this, we conducted an additional experiment and incorporated the results in **Supplementary Fig. 5**, with the corresponding citation of this figure added in the main text. Specifically, cement paste (with the same mix proportion as used in this study) was cast into two circular Petri dishes. For one of them, an SA solution (with the same concentration and dosage per unit area as used in our study) was applied on the surface, while the other was treated with an equal mass of water. After 1 minute, a second layer of cement paste was poured onto the surface of both dishes. After 24 hours, the samples were demolded. We observed that the two layers in the water-treated dish were fully bonded together, whereas the two layers in the dish treated with SA solution remained separated. This clearly demonstrates that the hydrogel formed from the SA solution effectively prevented bonding between the upper and lower cement paste layers. The related results are shown below for your reference.

Once again, we sincerely appreciate your insightful comments, and we hope that our revisions and additional experiments meet your expectations.

References

[1] Q. Li, Y. Li, X. Ma, Q. Du, K. Sui, D. Wang, C. Wang, H. Li, Y. Xia, Filtration and adsorption properties of porous calcium alginate membrane for methylene blue removal from water, Chemical Engineering Journal 316 (2017) 623-630.

[2] M. Yang, Y. Xia, Preparation and characterization of nano-SiO2 reinforced alginate-based nanocomposite films (II), Journal of Applied Polymer Science 134(38) (2017).

[3] M.J. Costa, A.M. Marques, L.M. Pastrana, J.A. Teixeira, S.M. Sillankorva, M.A. Cerqueira, Physicochemical properties of alginate-based films: Effect of ionic crosslinking and mannuronic and guluronic acid ratio, Food Hydrocolloids 81 (2018) 442-448.

[4] D. Snoeck, B. Moerkerke, A. Mignon, N. De Belie, In-situ crosslinking of superabsorbent polymers as external curing layer compared to internal curing to mitigate plastic shrinkage, Construction and Building Materials 262 (2020).

[5] K. Xia, Y. Chen, Z. Zhang, W. Wang, Y. Chen, L. Jia, Z. Jia, S. Quan, Y. Zhang, In-situ crosslinked nano SiO2 reinforced alginate bio-textile for mitigating plastic shrinkage in 3D printed concrete, Additive Manufacturing, 2025.

Lines 102-106, Page 3, Main Text-Clean Version

"Owing to the abundant release of calcium ions from the surface of fresh cement paste, SA can rapidly and spontaneously crosslink on the surface without the need for any additional treatment [64, 65] (Step 1 in **Fig. 1 b, supplementary Figs. 3-4**). This crosslinked hydrogel provides the basis for preventing interlayer bonding in the cement paste (**Supplementary Fig. 5**) and for creating well-ordered weak interfaces."

Relevant revised content

Supplementary Fig. 5, Supplementary Information

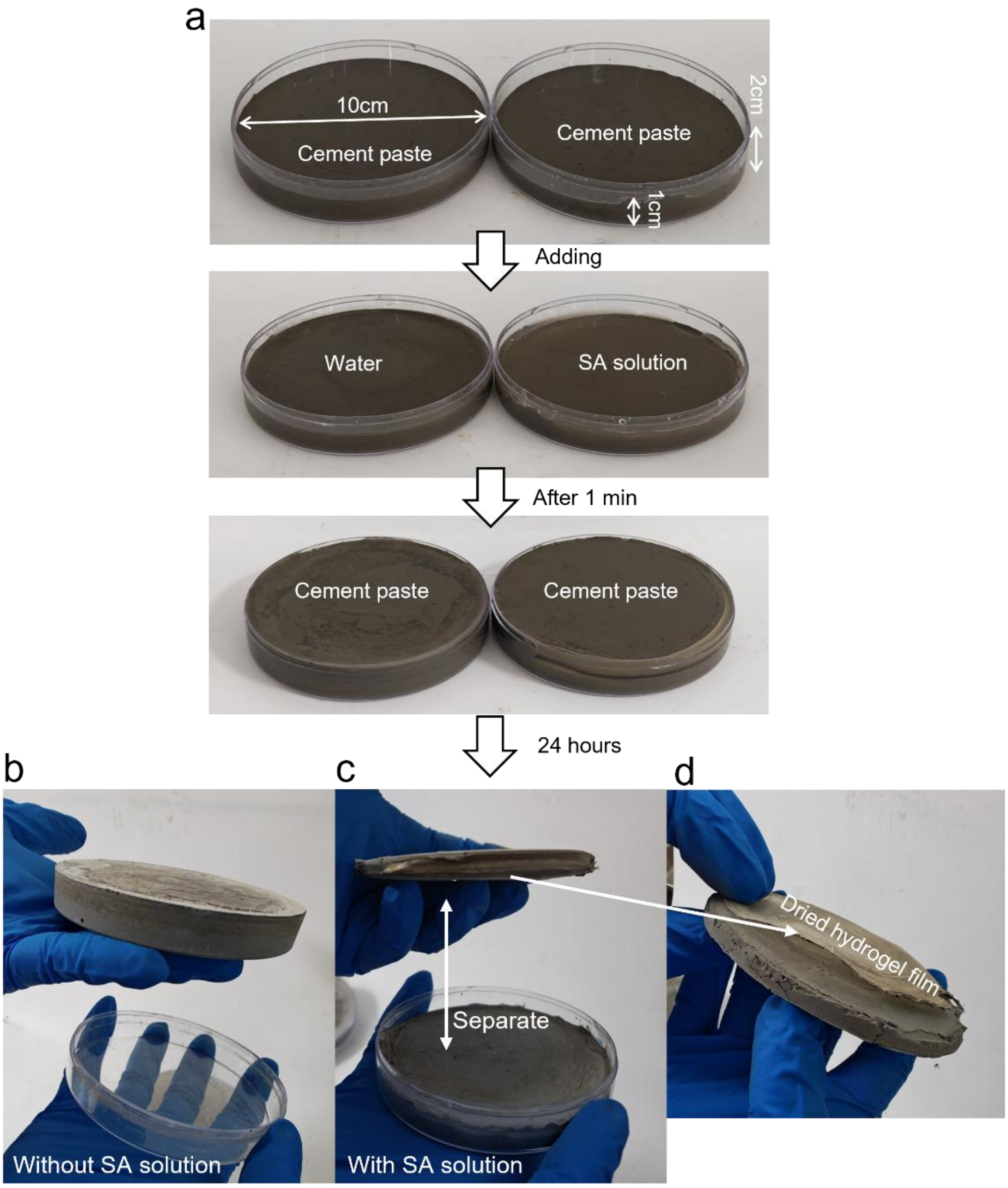

Supplementary Fig. 5. Barrier capability of the SA-crosslinked hydrogel between cement layers. a Two Petri dishes (10 cm in diameter, 2 cm in depth) were filled with 1 cm-thick cement paste. Equal masses of 5 wt% SA solution and water were then added onto the surfaces of the cement pastes, respectively. After 1 minute, another 1 cm-thick layer of cement paste was poured on each top. **b** After 24 hours, the two cement paste layers in the dish with added water were fully bonded together. **c** After 24 hours, the two cement paste layers in the dish with added SA solution were separated easily. **d** A distinct thin film formed after drying was observed on the bottom surface of the separated upper cement layer.

3. Water transport from the SA layer (line 194).

The phrase "SA hydrogel undergoes water separation" is unclear. Terms such as "dehydration" or "fluid desorption from the SA layer" would be more precise. This discussion would also benefit from referencing the concept of hydrogel-based internal curing of concrete. In this established approach, water diffuses from hydrogel inclusions into the cement matrix during early hydration as internal relative humidity decreases, driven by osmotic pressure gradients. This controlled water release mitigates self-desiccation and shrinkage, reduces microcracking, and improves mechanical properties—particularly in low water-to-cement ratio

systems like the w/c = 0.3 mixture used here. It is therefore plausible that the SA layers act as internal curing agents over time, which may help explain the excellent mechanical performance of the PPAC. Definitively testing this hypothesis would require a control in which the polymer layer neither swells nor releases water (e.g., an inert plastic interlayer), which may be challenging and is not necessarily required for this study.

Relevant references from the hydrogel-based internal curing literature include:
https://www.sciencedirect.com/science/article/abs/pii/S0008884621002970
https://link.springer.com/article/10.1617/s11527-014-0308-5
https://www.sciencedirect.com/science/article/abs/pii/S0950061816300927
https://link.springer.com/article/10.1617/s11527-018-1241-9

Response:

Thank you very much for your valuable comment. We fully agree with your suggestion and have revised this term "water separation" in **line 222** to "dehydration" to improve clarity. In addition, following your advice, we have added a brief discussion in **lines 224–229, Page 9, Main Text** on the internal curing effect that can be possibly induced by fluid desorption from the SA layer, and have cited the recommended relevant references to support this point. We find this point particularly interesting and believe it offers valuable insights for future research. However, as it is somewhat beyond the main scope and focus of the present study, we have only included a brief discussion rather than conducting additional experiments to fully substantiate this mechanism. We agree that this is an important topic that would merit a dedicated follow-up study to further explore the benefits of such internal curing behavior. We sincerely appreciate your insightful suggestion and hope that the revised version meets your expectations.

Relevant revised content

Lines 226-231, Page 9, Main Text-Clean Version

"Meanwhile, during early-age hydration of cement, as the internal relative humidity decreases, fluid desorption from the SA layer may also promote the diffusion of water from the hydrogel into the cement matrix. This release of water may partially mitigate self-desiccation and shrinkage of the cement paste, thereby providing an internal curing effect, which can also improve the mechanical properties [68-71]. However, as this is not the primary focus of the present study, it is not discussed in detail here."

Added references: [68-71]

[68] Schröfl, C., et al., Recent progress in superabsorbent polymers for concrete. Cement and Concrete Research, 2022. 151 DOI: 10.1016/j.cemconres.2021.106648.

[69] Zhu, Q., C.W. Barney, and K.A. Erk, Effect of ionic crosslinking on the swelling and mechanical response of model superabsorbent polymer hydrogels for internally cured concrete. Materials and Structures, 2014. 48(7): p. 2261-2276 DOI: 10.1617/s11527-014-0308-5.

[70] Mignon, A., et al., Alginate biopolymers: Counteracting the impact of superabsorbent polymers on mortar strength. Construction and Building Materials, 2016. 110: p. 169-174 DOI: 10.1016/j.conbuildmat.2016.02.033.

[71] Wyrzykowski, M., et al., Recommendation of RILEM TC 260-RSC: using superabsorbent polymers (SAP) to mitigate autogenous shrinkage. Materials and Structures, 2018. 51(5) DOI: 10.1617/s11527-018-1241-9.

There do not appear to be any major issues with data analysis, interpretation, or overall conclusions. The methodology is sound, aligns with standards in the civil engineering and concrete materials science community, and is described with sufficient detail to allow reproducibility.

Several minor points could further improve clarity and presentation:

1. The cartoons in Fig. 5d are visually appealing but not essential.

2. Line 227: correct material names to polypropylene and polycarbonate.

3. Line 56: consider noting that similar layered architectures can also be achieved via 3D printing, referencing existing citations (e.g., Ref. 24).

4. The manuscript is generally well written, but minor grammatical and word-choice edits would further improve clarity.

Response:

Thank you very much for providing these constructive comments on our manuscript. Your suggestions have been extremely helpful in improving its clarity and overall quality. Our point-by-point responses are as follows:

1. We fully understand your expectation that the manuscript should convey the core innovative idea and its supporting evidence in the most concise manner. Indeed, Fig. 5 d represents a more generalized conceptual illustration, and we acknowledge that it does not provide direct evidence or technical explanation for the proposed mechanism. However, this figure visually conveys the application scenario and significance of our concept in a straightforward way, which may help readers outside the academic community or those less familiar with cement-based materials to better grasp the essence of our innovation. We also noted that many articles published in *Nature Communications* adopt similarly intuitive conceptual figures to communicate their key messages. We believe that retaining this figure may broaden the readership and benefit readers who mainly focus on the conceptual contribution without delving into technical details. Therefore, we respectfully prefer to keep this figure in the manuscript, and we sincerely appreciate your understanding and support.

2. We apologize for the mistake in the initial manuscript. Following your suggestion, we have corrected the error accordingly. Thank you for pointing this out.

3. Thank you for your careful reading. As advised, we have added relevant description and citations in **line 56-58, Page 2** accordingly.

4. We appreciate your positive feedback regarding the writing quality of the manuscript. In response to your comment, we have once again thoroughly reviewed and refined the entire manuscript to further improve clarity and readability. We hope that the revised version meets your expectations.

Relevant revised content

Line 56-58, Page 2, Main Text-Clean Version

"However, existing high-performance architected cementitious materials often rely heavily on complex fabrication methods, such as 3D printing [22, 24], ice-templating [6, 20], hot pressing [21], laser-cutting [5] and prefabricated mold casting [26]."

Added citations: [22 and 24]

[22] Prihar, A., et al., Tough double-bouligand architected concrete enabled by robotic additive manufacturing. Nat Commun, 2024. 15(1): p. 7498 DOI: 10.1038/s41467-024-51640-y.

[24] Moini, R., et al., *Mechanical properties and fracture phenomena in 3D-printed helical cementitious architected materials under compression.* Materials and Structures, 2024. 57(7).

Reviewer #2

The current work introduces "puff pastry" architected material with improved mechanical and thermal insulating properties. This work is nicely executed and contributes to the field of tough and ductile architected cementitious materials. The mechanical testing (MOR, ductility, and fracture toughness) is comprehensive, and the results are effectively supported by microstructural analysis. The use of Micro-CT and SEM to connect toughening mechanisms (such as crack deflection and interlocking) to fracture behavior is nicely performed. The microstructural analysis and the corresponding discussion regarding the relative length of the weakness phase compared to the thickness of the solid phase explain the toughening mechanisms in specimens with different folds (3, 5, & 7 folds). The study also demonstrates additional applications through thermal insulation characterization and connects these results to microstructural features. The fabrication process based on rolling during the plastic stage is new and interesting. Furthermore, rheological analysis of cement paste and investigation of sodium alginate hydrogel interactions with cement paste are valuable additions. However, there are the following questions that should be addressed. The work shows strong potential; however, I recommend a major revision to adequately address the following comments.

Response:

Thank you very much for your thorough evaluation of our study and for your positive feedback. We have carefully considered each of your comments and highly appreciate your insights. Your constructive suggestions have been extremely helpful in improving the quality and clarity of our manuscript. In response to your comments, we have made detailed revisions with the aim of enhancing the manuscript in accordance with your recommendations. We hope that the revised version meets your expectations.

1. Since this study uses hydrogels, it would be beneficial to include a discussion of recent hydrogel-cementitious composite studies, such as Wang, Han, et al., npj Materials Sustainability 3.1 (2025): 28 and Wang, Han, et al., Cement and Concrete Research 195 (2025): 107926.

Response:

Thank you very much for your constructive comments. We acknowledge that our discussion on this part was previously insufficient. Therefore, following your suggestions, we have added the relevant discussion in the Introduction (**Lines 64–69, Page 2, Main Text**) and have cited the literatures you recommended.

Relevant revised content

Line 64-69, Page 2, Main Text-Clean Version

"In recent years, the design of hydrogel–cementitious composites have shown great compatibility and potential in toughness-enhancement strategies for cement-based materials, by enabling the creation of layered structures, increasing the energy-absorption capacity of the matrix, and improving the material's strain capacity [6,35,36]. However, current approaches are facing challenges in simultaneously achieving adequate performance improvements, mild processing conditions, and good scalability, and are therefore largely restricted to laboratory-scale studies, hindering large-scale implementation."

Added citations: [35 and 36]

[35] Wang, H., et al., Toward tough and sustainable hydrogel-cement composites via biomineralization-inspired strategy. npj Materials Sustainability, 2025. 3(1) DOI: 10.1038/s44296-025-00072-w.

[36] Wang, H., et al., Hydrogel-guided dispersion strategy for enhancing the toughening efficiency of liquid polymers in cement paste. Cement and Concrete Research, 2025. 195 DOI: 10.1016/j.cemconres.2025.107926.

2. Number of folds appears to significantly influence performance. Fold-7 shows better performance than Fold-3 and Fold-5, while the difference between Fold-3 and Fold-5 in terms of specific KJC and MOR is not significant (Fold-5 even shows slightly lower averages). Since Fold-7 demonstrates clear improvement, it would be useful to comment on whether increasing the number of folds further could continue improving fracture toughness, or whether fabrication limits exist due to material properties. Microstructural observations suggest that higher folds reduce the relative thickness of the solid phase and enhance interlocking, which may further improve fracture toughness in higher folds (8, 9, 10, and so on) specimens.

Response:

We sincerely thank you for this valuable and insightful comment. We have indeed considered this issue during our study. One important reason for presenting results at folding cycles of 3, 5, and 7 is that, under our current material system and processing conditions, the maximum achievable number of stable rolling–folding cycles is seven. Beyond this point, we observed a significant deterioration in processability. With the existing rolling equipment and fabrication procedure, it becomes difficult to perform additional rolling cycles while maintaining the structural integrity of the material. This limitation has been clarified in **Lines 181~186, Page 6, Main Text**. Moreover, we consider exploring the performance limits of PPAC to be an important direction for future research, as highlighted in the "**Discussion and open questions**" section at the end of the manuscript.

Relevant revised content

Lines 181~186, Page 6, Main Text-Clean Version

"However, it should be noted that although the results demonstrate improved mechanical performance with increasing rolling-folding cycles, this study only presents results up to a maximum of 7 folds. This limitation arises from the significant deterioration in processability beyond seven cycles, making it difficult to maintain a stable rolling–folding process with the equipment used in this work. In principle, PPAC is expected

to exhibit a performance limit associated with the number of cycles for specific mixtures. However, this remains to be systematically investigated in the future."

3. The size effect is important in cementitious materials. Although the total number of folds is increased while keeping specimen size constant, fracture testing is performed only on samples with 40 mm thickness. The large-scale PPAC plate (40 cm × 40 cm × 4 cm) presented in the Supplementary Information is a good addition, but discussion of size effects on fracture properties and strength would strengthen the study.

Response:

We sincerely thank you for this valuable comment. The size effect is indeed a highly practical and important issue, as it directly relates to the applicability of PPAC in large-scale construction. In this study, although we demonstrated the feasibility of fabricating relatively large PPAC panels, the potential influence of specimen thickness on mechanical performance was not sufficiently addressed. Given that the toughening mechanism of PPAC arises from crack deflection within its layered architecture, thickness is expected to play a critical role. To address this, we have conducted additional experiments and included a dedicated discussion in **Supplementary Discussion 2**.

Specifically, PPAC specimens with thicknesses of 20 mm and 60 mm were prepared and compared with the original 40 mm specimens. To minimize structural variations, specimens were first fabricated following the same procedure described in the main text (PPAC fold-7, 40 mm). Then, during the plastic stage, two 40 mm PPAC fold-7 specimens were stacked and cured to obtain an 80 mm specimen, which was subsequently cut down to 60 mm. The 20 mm specimens were obtained by cutting the original 40 mm PPAC fold-7 specimens. This approach ensures that all groups share a comparable layered architecture, with thickness being the primary variable.

Single-edge notched bending (SENB) tests were then performed. The results show that the K_{IC} values do not exhibit significant differences across different thicknesses, which is reasonable since crack initiation is mainly governed by intrinsic material properties rather than structural dimensions. However, the load–displacement curves indicate that the 20 mm specimens exhibit smaller deflection at failure, primarily due to their reduced size.

For fracture toughness evaluation in terms of $\mathbf{K_{JC}}$, J–R curves were constructed. Due to thickness differences, ASTM criteria were not applied for direct comparison; instead, the final K_{JC} values at fracture were compared. The results show that the 60 mm and 40 mm specimens exhibit no significant difference in K_{JC} values, whereas the 20 mm specimens show a significantly lower K_{JC}. This behavior can be rationalized by the underlying toughening mechanism: the enhancement in PPAC arises from repeated crack deflection across weak interfaces. When the layer thickness remains similar, thicker specimens contain more interfaces along the crack propagation path, leading to greater energy dissipation. In contrast, the 20 mm specimens likely contain roughly half the number of layers compared to the 40 mm specimens, resulting in reduced toughening effect. The absence of further improvement from 40 mm to 60 mm suggests that the relationship between interface number and toughness may be not strictly linear.

Overall, these results indicate the dependence of PPAC on specimen thickness and the number of internal layers. Nevertheless, due to time and workload constraints, this remains a preliminary investigation.

A comprehensive understanding of size effects will require more extensive experimental data and systematic study, which we consider an important direction for future work.

Relevant revised content

Supplementary discussion 2. Size effect on PPAC performance. Supplementary Information.

To evaluate the influence of size effect on PPAC performance, specimens with different thicknesses (20 mm, 40 mm and 60 mm, **Supplementary Fig. 32 a**) were investigated. The 40 mm data correspond to the PPAC fold-7 specimens presented in the main text. To minimize variables, all specimens were prepared following the same procedure. Specifically, specimens were first fabricated using the protocol described in the main text with 7 rolling–folding cycles (same as PPAC Fold-7). During the plastic stage, two 40 mm thick specimens were stacked and cured to obtain an 80 mm thick specimen, which was subsequently cut to 60 mm. The 20 mm thick specimens were obtained by cutting the original 40 mm thick PPAC fold-7 specimens. This preparation strategy ensures that all specimens share a comparable layered architecture, with specimen thickness being the primary variable.

Single-edge notched bending (SENB) tests were then performed (**Supplementary Fig. 32 b**). The notch depth and span-to-thickness ratio were kept consistent with those used in the main text. The results show that the K_{IC} values do not exhibit significant differences across different thicknesses (**Supplementary Fig. 32 c, Supplementary Table 11**), which is expected, as crack initiation is primarily governed by intrinsic material properties rather than specimen dimensions. The load–displacement curves (**Supplementary Fig. 32 b**) indicate that the 20 mm thick specimens exhibit smaller deflection at failure, mainly due to their reduced thickness.

Due to the differences in specimen thickness, ASTM limit was not used to compare K_{JC} values. Instead, the K_{JC} values at final failure were used (**Supplementary Fig. 32 d**). The results show that the 60 mm and 40 mm thick specimens exhibit no significant difference in K_{JC} values, whereas the 20 mm thick specimens show significantly lower K_{JC} (**Supplementary Fig. 32 e, Supplementary Table 12**). This behavior can be attributed to the underlying toughening mechanism of PPAC, which relies on crack deflection along weak interlayer interfaces. When the layer thickness remains similar, thicker specimens can contain more interfaces along the crack propagation path, leading to increased energy dissipation and enhanced toughness. In contrast, the 20 mm thick specimens likely contain approximately half the number of layers compared to the 40 mm thick counterparts, resulting in reduced toughening effect. Meanwhile, the absence of further improvement from 40 mm to 60 mm suggests that the relationship between interface number and toughness may not be strictly linear.

Overall, these results indicate the dependence of PPAC performance on specimen thickness and the number of internal layers. However, this analysis remains preliminary. A comprehensive understanding of size effects will require more extensive experimental data and systematically designed test groups, which warrants further investigation.

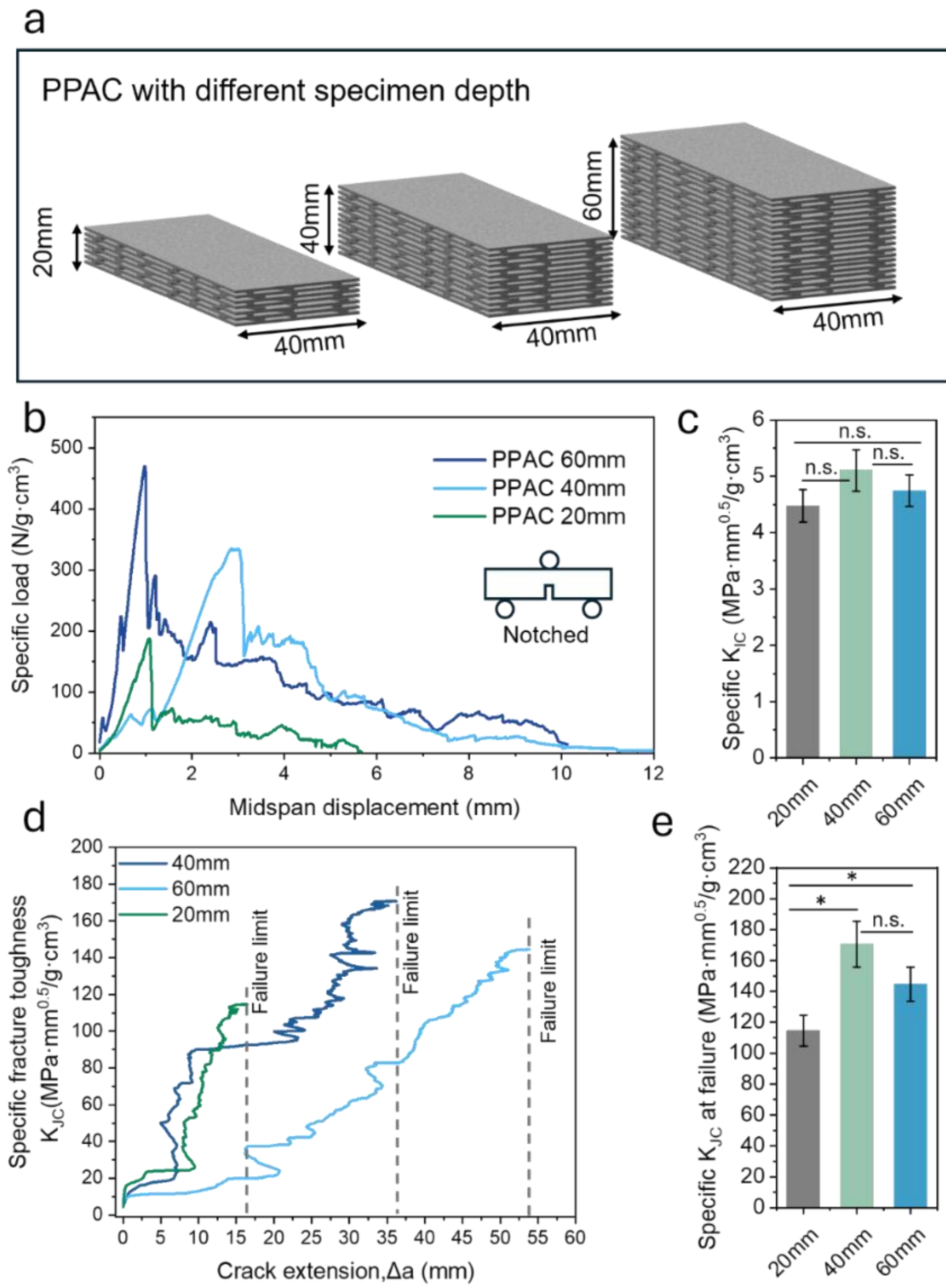

Supplementary Fig. 32 Size effect on the mechanical properties of PPAC. a Schematic diagram of specimen dimension. **b** SENB test results of PPAC with different specimen depth. **c** Specific K_{IC} results. **d** Crack-resistance curves (J-R curves) of PPAC with different specimen depth. **e** Specific K_{JC} value at failure limit.

Supplementary Table 13. Data set and t-test significance analysis results of the K_{IC} results in Supplementary Fig. 32

Sub Table a. Data set

Specimen	Mean value	SD	Size (n)
20mm	4.47599	0.29	3
40mm	5.10306	0.38	3
60mm	4.74513	0.28	3

Sub Table b. Significance analysis

Comparison	Mean difference	T-value	df	p-value	Significance
40 mm vs 20 mm	0.62707	2.27	3	0.11	n.s.
60 mm vs 20 mm	0.26914	1.15	3	0.33	n.s.
40 mm vs 60 mm	0.35793	1.32	3	0.27	n.s.

* ($p<0.05$) indicates a significant difference; ** ($p<0.01$) indicates a highly significant difference; n.s. means no significant difference.

Supplementary Table 14. Data set and t-test significance analysis results of the K_{IC}-failure results in Supplementary Fig. 32

Sub Table a. Data set

Specimen	Mean value	SD	Size (n)
20mm	114.6	10.02	3
40mm	170.67	14.82	3
60mm	144.59	11.07	3

Sub Table b. Significance analysis

Comparison	Mean difference	T-value	df	p-value	Significance
40 mm vs 20 mm	56.07	5.42	3	0.012	*
60 mm vs 20 mm	29.99	3.47	4	0.026	*
40 mm vs 60 mm	26.08	2.44	3	0.09	n.s.

* ($p<0.05$) indicates a significant difference; ** ($p<0.01$) indicates a highly significant difference; n.s. means no significant difference.

4. How many repetitions were performed for each test? Were statistical significance tests performed for the results shown in Figure 2? For example, Line 122 states that a parameter "was not significantly affected." Please clarify whether this refers to statistical significance, and please include tests if they were not conducted.

Response:

Thank you very much for your constructive comments and kind reminder. We apologize for not clearly reporting the number of experimental replicates and the results of statistical significance tests in the initial version. In this study, all data shown in the bar graphs are presented as mean ± standard deviation (SD), with each dataset derived from at least three parallel specimens. Additionally, we have supplemented the analysis with t-tests to indicate statistical significance, and significance markers have been added to **Fig. 2 b, c, d, f, g, h and Fig. 3 e**. We have included a description of this in **lines 412~416, Page 16~17, Main Text**, to clarify the sample size and the method of significance analysis used in this study. To further enhance the reliability of our data, we have provided the data for these figures as well as the t-test results, including T values, P values, degrees of freedom and significance result in **Supplementary Tables 4–10**. We hope these revisions meet your expectations and sincerely thank you for your guidance.

Relevant revised content

Lines 414-418, Page 16~17, Main Text-Clean Version

"**Statistical analysis.** Data in the bar figures is presented as mean±SD. For the statistical methods such as mechanical test and hot disk test, a minimum of 3 repetitions were performed for each test. The t-tests were performed to confirm the significance between the results. Confidence interval of 95% and α value of 0.05 was used. $P < 0.05$ indicates a significant difference (*) and $P < 0.01$ indicates a highly significant difference (**). The calculated t value, p value and significance are presented in **Supplementary Tables 4-10.**"

Modified figures

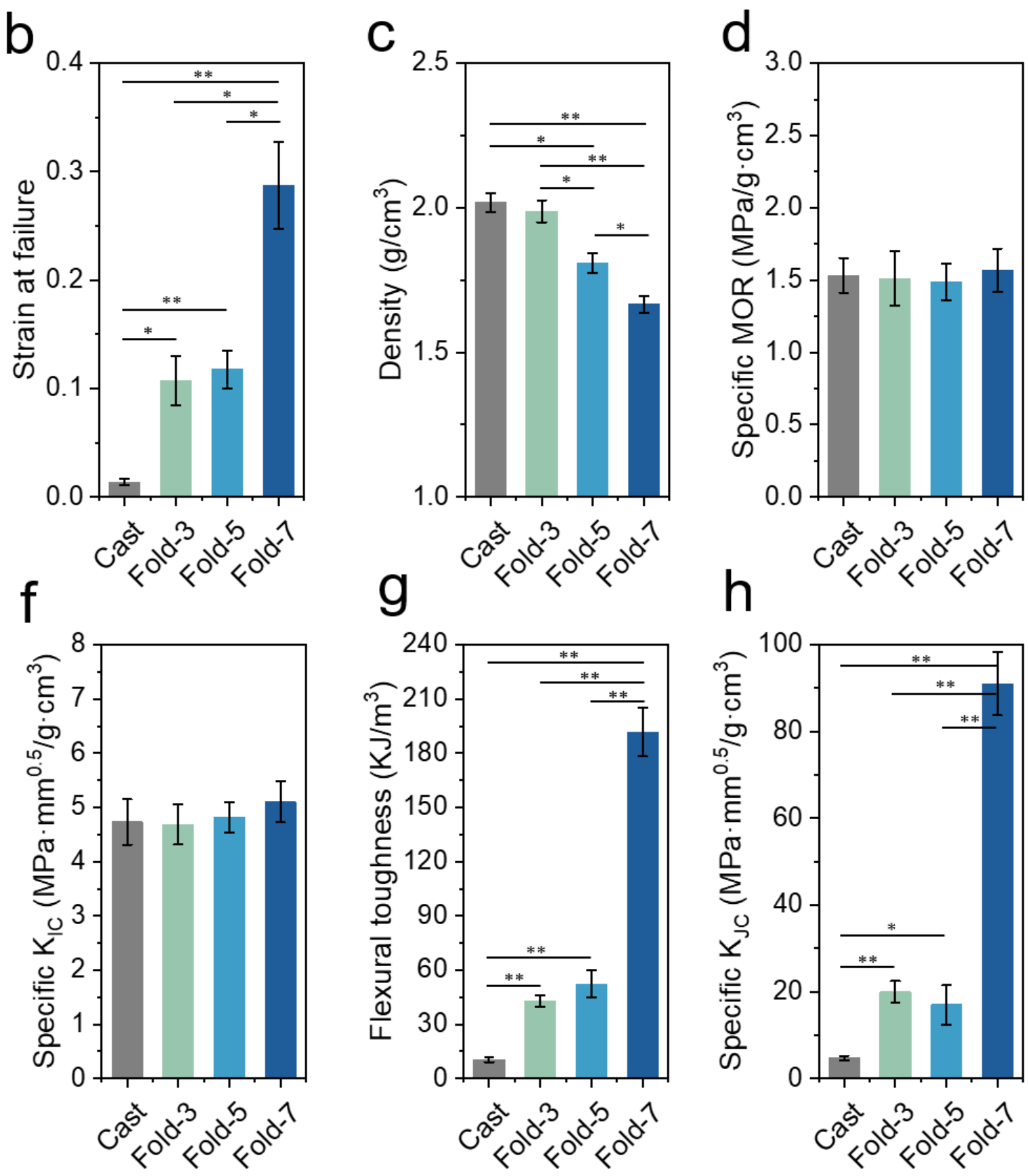

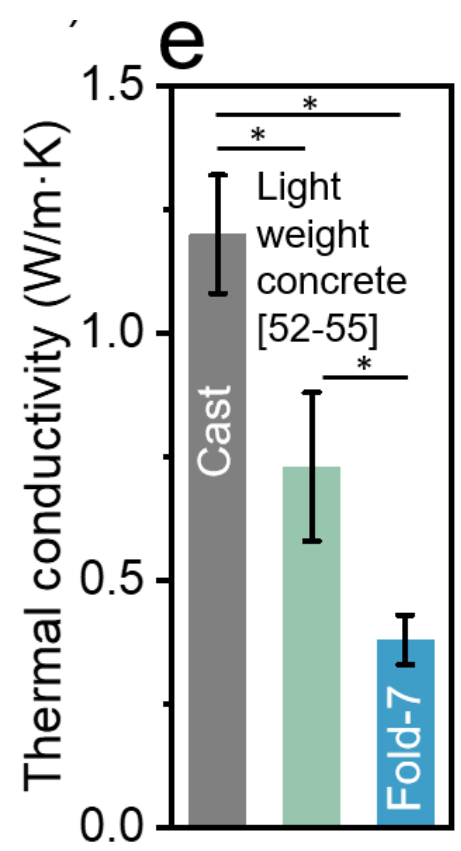

Fig. 2 b, c, d, f, g, h **Fig. 3 e**

No significance mark means no significant difference between results

5. Please clarify how flexural toughness is calculated (Line 132). Also, please justify the use of a span-to-depth ratio of 2.5, as flexural tests and SENB typically use ratios of 3 or higher. With a lower span-to-depth ratio, the flexural test becomes shear dominated.

Response:

Thank you very much for your detailed review and constructive feedback. Regarding the evaluation of flexural toughness, we referred to Refs. [1–2]. Although this flexural-toughness has been widely used for ductile materials such as metals and polymers, its suitability for brittle materials still requires broader discussion. Nevertheless, Refs. [1–2] are also studies on toughened cementitious materials and employed the flexural-toughness evaluation method. Therefore, to maintain a certain level of comparability with the existing literatures, we also present this metric in our study. The specific calculation procedure has now been added to the **Supplementary method 1**.

However, we would like to emphasize that the $K_{(JC)}$ value calculated from the SENB method prescribed in the ASTM standard is, in our view, a more scientific and reliable toughness indicator for brittle materials such as cementitious materials and ceramics. Accordingly, the core performance indicators in this study are presented mainly following the ASTM protocol, which is also consistent with many biomimetic toughening studies, e.g., Refs. [3–5].

We sincerely appreciate your comment on the specimen dimensions used in our SENB tests. We fully understand that the span-to-thickness ratio recommended in the ASTM standard is intended to ensure a pure bending stress state, thereby enabling a more accurate evaluation of toughness. In our study, the span was 100 mm and the specimen thickness was 40 mm. We acknowledge that this ratio is indeed lower than the ASTM recommendation and imposes certain limitations on the results.

Nevertheless, we would like to explain the rationale behind this selection. First, as the fundamental topic of this work is cement-based materials, therefore we initially referred to widely adopted cement-based material testing standards. According to the Chinese standard GB/T 17671-2021, flexural strength is tested using 40 mm × 40 mm × 160 mm specimens under a 100-mm span, which is a standard condition. Therefore, we prepared specimens of this size and used a 100-mm span to measure MOR, aiming to ensure comparability with traditional studies in this field. Second, due to the constraints of our previous experimental setup, the three-point bending fixture of our loading system was equipped with a fixed span of 100 mm, which could not be adjusted. This is a limitation on our side, and we will upgrade our testing conditions in future work to address this issue.

The ASTM standard notes that a lower span-to-thickness ratio may lead to a certain degree of shear contribution to the stress field. However, according to fracture-mechanics principles, this shear contribution becomes less significant as the material becomes more brittle. The matrix in this work is cement paste without aggregates or fibers, meaning that fracture primarily occurs through the brittle matrix, and its apparent ductility mainly originates from crack deflection among weak cavities rather than ductile plastic shear. Therefore, it can be inferred that the reduced span-to-thickness ratio has a limited influence on the measured fracture toughness in our case. Moreover, the toughness enhancement achieved by our best specimen (Fold-7) is extremely significant, for example, the K_{JC} increased by approximately 20 times compared with the Cast group, indicating that the potential error introduced by the span-to-thickness ratio would not alter the fundamental conclusions on the toughening enhancement originated from the puff pastry architecture in this work.

We acknowledge that our selection of specimen dimensions lacked sufficient caution, which may have raised concerns for you and potential readers. We hope the above explanation clarifies our reasoning. Unfortunately, acquiring new testing equipment and repeating all related experiments within a short time frame is quite challenging for us. We have clearly stated the limitations associated with specimen dimensions in the Supplementary Information to avoid any potential misunderstanding for readers. We sincerely appreciate your understanding and thank you again for your valuable comments.

References

[1] Y. Chen, Y. Zheng, Y. Zhou, W. Zhang, W. Li, W. She, J. Liu, C. Miao, Multi-layered cement-hydrogel composite with high toughness, low thermal conductivity, and self-healing capability, Nature Communications 14(1) (2023).

[2] J. Chen, Z. Liu, S. Zhang, S. Hu, F. Wang, Energy-Efficient Fabrication of Biomimetic Materials for Sustainable Infrastructure Applications, Advanced Science (2025).
[3] S. Gupta, H.S. Esmaeeli, R. Moini, Tough and Ductile Architected Nacre-Like Cementitious Composites, Advanced Functional Materials (2024).

[4] S. Gupta, R. Moini, Tough Cortical Bone-Inspired Tubular Architected Cement-Based Material with Disorder, Advanced Materials 36(52) (2024).

[5] H.-L. Gao, S.-M. Chen, L.-B. Mao, Z.-Q. Song, H.-B. Yao, H. Cölfen, X.-S. Luo, F. Zhang, Z. Pan, Y.-F. Meng, Y. Ni, S.-H. Yu, Mass production of bulk artificial nacre with excellent mechanical properties, Nature Communications 8(1) (2017).

Relevant revised content

Supplementary method 1. Calculation process of mechanical parameters. Supplementary Information.

Flexural Toughness: The flexural toughness (KJ·m^{-3}) generally represents the energy absorbed by the material per unit volume of the bent region. According to literature [12, 13], the flexural toughness can be calculated by the area enclosed by the flexural stress-strain curve of the material during bending. The strain here is actually equivalent strain for the 3-point bending test specimen, which is calculated by:

$$\varepsilon_e = \frac{6\delta D}{L^2} \quad (2)$$

Where ε_e is the equivalent strain, δ is the midspan deflection, D is the depth of the specimen.

In 3-point bending, the flexural stress can be calculated by:

$$\sigma = \frac{3pL}{2WD^2} \quad (3)$$

Where p is the crosshead load. Then, the flexural toughness is calculated by:

$$Flexural\ Toughness = \sum_{n=0}^{Failure} \sigma_n \times \varepsilon_{e_n} \quad (4)$$

Added references: [12-13]

[12] Y. Chen, Y. Zheng, Y. Zhou, W. Zhang, W. Li, W. She, J. Liu, C. Miao, Multi-layered cement-hydrogel composite with high toughness, low thermal conductivity, and self-healing capability, Nature Communications 14(1) (2023).

[13] J. Chen, Z. Liu, S. Zhang, S. Hu, F. Wang, Energy-Efficient Fabrication of Biomimetic Materials for Sustainable Infrastructure Applications, Advanced Science (2025).

6. In Fig. 2j (R-curve), crack extension may decrease locally due to compliance changes, which is understandable. However, KJC also decreases at certain points for Fold-7 in the R-curve. According to Supplementary Equation 8, Apl increases with crosshead displacement while other parameters (W, D, a0)

remain constant, so Jpl and KJC should increase. Please review the plot or clarify whether instantaneous crack length is used in Equation 8.

Response:

We sincerely apologize for this issue. After carefully re-examining the figure, we found that the curve for Fold-7 in Fig. 2 j was incorrectly presented using a smoothed plot. The plotting software we used was Origin 2018, with a Loess smoothing model and a window size of 0.01. This processing introduced unintended alterations to the curve that should not have occurred (as shown in the figure below).

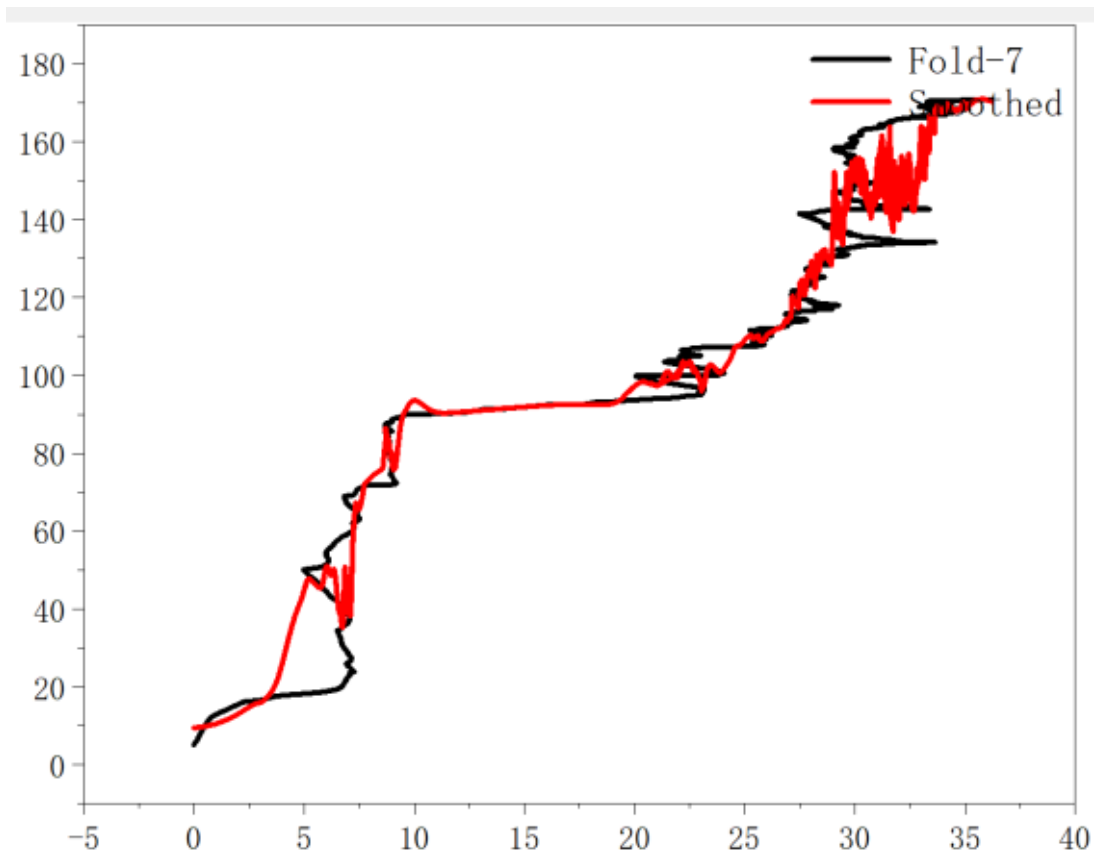

We sincerely apologize for this error. We have corrected it and now present the accurate curve in the main text. The revised content can be conveniently found in the "Relevant content" section following this response. In addition, we have carefully reviewed all related data in the manuscript and confirmed that this issue does not have any further impact on the results or conclusions of the study.

Relevant revised content

Fig. 2 j

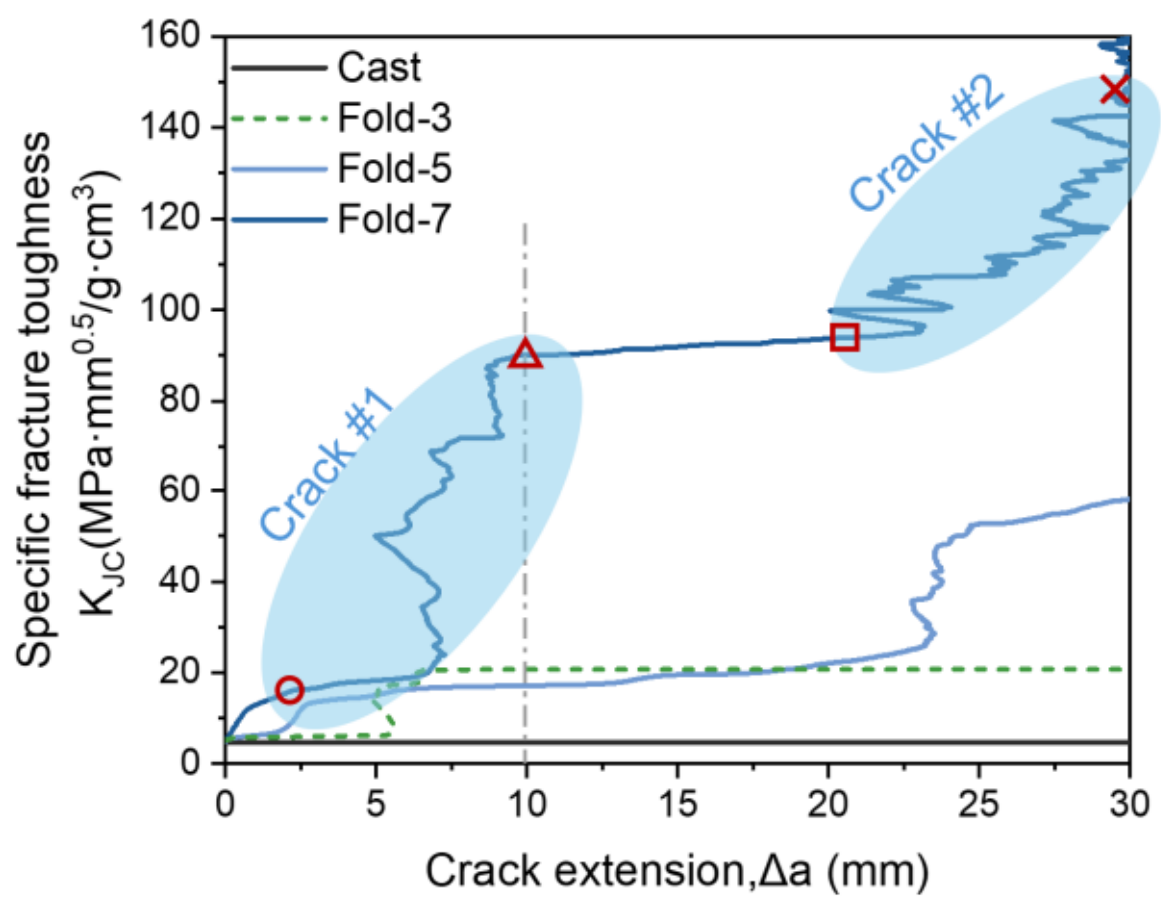

Supplementary Fig. 12

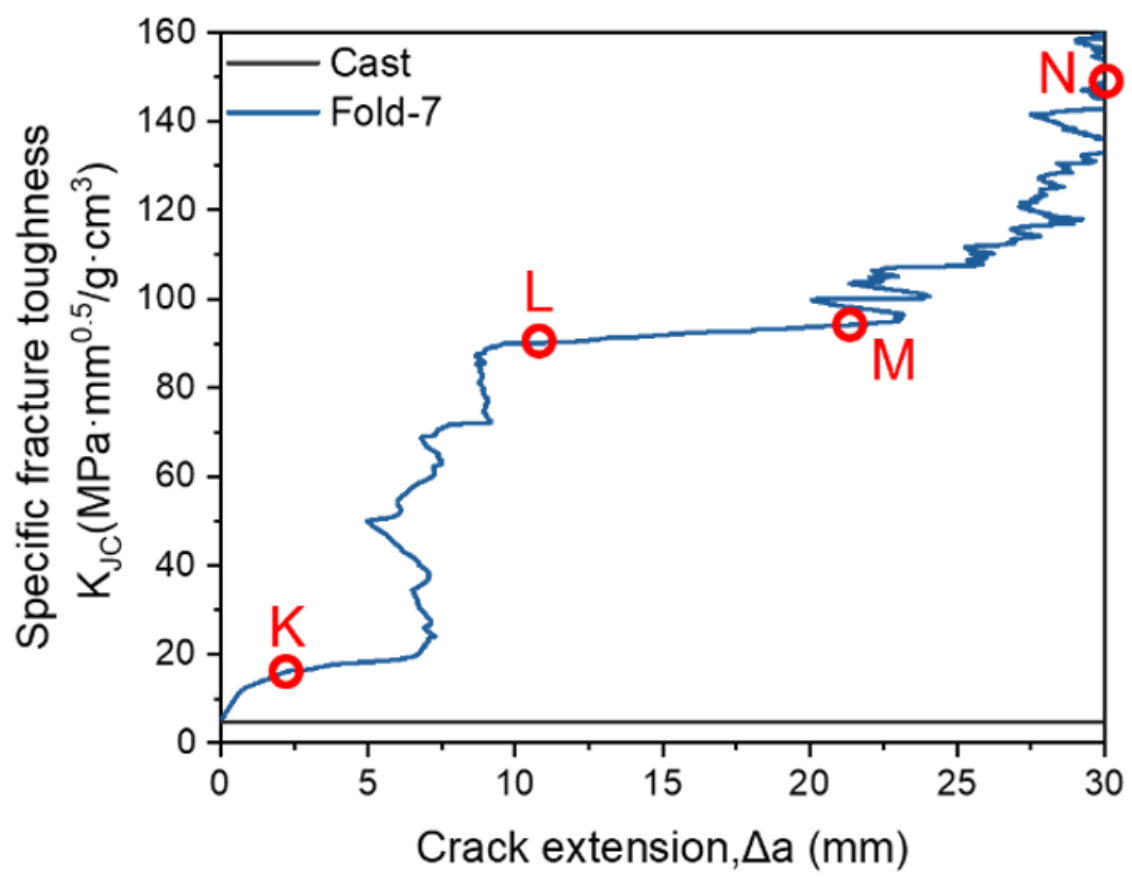

7. How would the samples perform when loaded in the other two perpendicular orientations? A brief discussion would be helpful.

Response:

We sincerely thank you for your valuable suggestion. In this study, the designed PPAC achieves enhanced toughness during bending through crack deflection enabled by multiple parallel weak interfaces. Therefore, the mechanical performance presented in the main text corresponds to loading applied perpendicular to the interfaces. However, your suggestion is both interesting and meaningful. Accordingly, we have supplemented additional mechanical tests with loading applied parallel to the interfaces. The results show that, in this configuration, the multilayer weak-interface architecture no longer promotes crack deflection. Instead, the interfaces may act as preferential weak planes for crack initiation, leading to a reduction in flexural performance compared with the cast counterpart.

This behavior is consistent with expectations, as the directionally arranged architecture of PPAC inherently results in pronounced mechanical anisotropy. These results are presented in **Supplementary Fig. 11**. In addition, we have clarified in the main text (**Lines 123–124, Page 5, Main Text**) that the toughening effect of PPAC is only observed when bending is applied perpendicular to the interfaces, and we briefly reference the results under parallel loading.

We hope these additions improve the clarity and rigor of the manuscript.

Relevant revised content

Supplementary Fig. 11. Supplementary Information

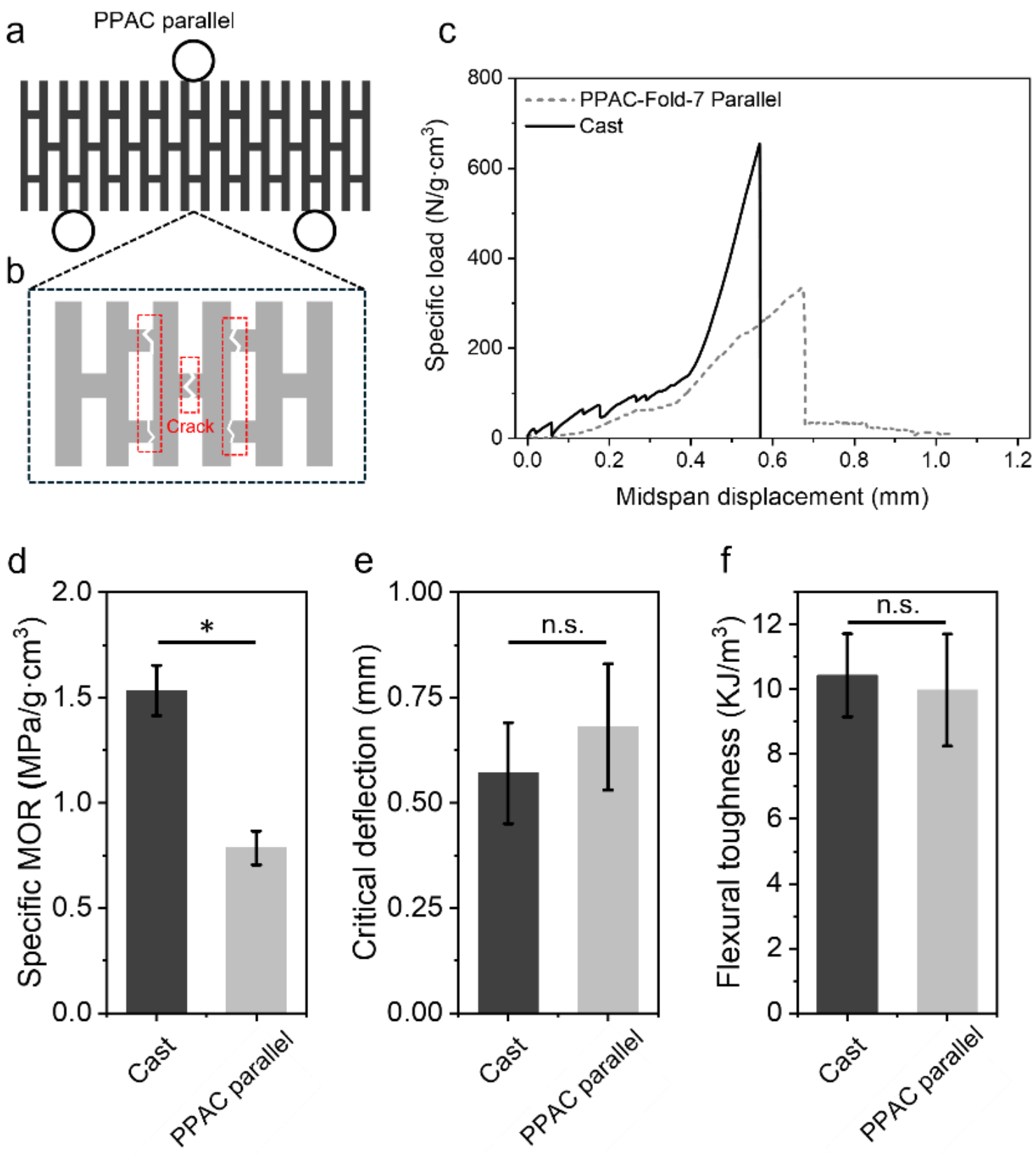

Supplementary Fig. 11. Mechanical properties of PPAC-Fold-7 when bending load is applied parallel to the weak interfaces. a Schematic diagram of the loading direction. **b** Schematic diagram of the potential fracture behavior at the material architectural scale. **c** 3-point bending (3PB) test curves. **d-f** Comparison on specific MOR, critical deflection and flexural toughness between cast the PPAC parallel, respectively. The results indicate that PPAC does not exhibit a toughness enhancement when loaded parallel to the interfaces. Moreover, as the SA-induced interfaces act as preferential weakness for crack, the MOR of PPAC can be significantly lower than that of the cast counterpart. The statistical significance analysis is listed in **Supplementary Table 2.**

Supplementary Table 2. Data set and t-test significance analysis results of the mechanical properties in Supplementary Fig. 11

Sub Table a. Data set (mean±SD)

Properties	Cast	PPAC perpendicular	Size (n)
Critical deflection	0.57±0.12	0.68±0.13	3
Specific MOR	1.53±0.12	0.79±0.08	3
Flexural toughness	10.44±1.28	9.98±1.73	3

Sub Table b. Significance analysis (Cast vs PPAC perpendicular)

Properties	Mean difference	T-value	df	p-value	Significance
Critical deflection	0.11	0.99	3	0.39	n.s.
Specific MOR	0.74	8.88	3	0.003	**
Flexural toughness	0.46	0.37	3	0.74	n.s.

* ($p<0.05$) indicates a significant difference; ** ($p<0.01$) indicates a highly significant difference; n.s. means no significant difference.

Lines 127-128, Page 5, Main Text-Clean Version

"Consequently, the toughness of PPAC is enhanced under bending perpendicular to the interfaces (see parallel loading results in **Supplementary Fig. 11**)……"

8. Lines 144 and 146: Please clarify the meaning of "hierarchical failure". Typically, hierarchical cracks refer to multi-scale fracture networks composed of interconnected cracks of different lengths. The DIC results appear to show one primary deflected crack and one branch; describing this as hierarchical may be excessive.

Response:

Thank you very much for your kind reminder. We sincerely apologize for any confusion caused by the inappropriate wording and for the potential overstatement of our conclusions. Following your suggestion, we have removed all instances of "hierarchical failure" and replaced them with more accurate and appropriate descriptions. Specifically, we now use **"progressive fracture"** to describe the gradual crack propagation process, and **"multi-stage fracture"** to characterize the overall fracture behavior of the material, as reflected by the multiple rising segments in the J–R curve.

9. Line 169 and Fig. 3b: Please specify whether the reported value corresponds to the maximum or average backside temperature.

Response：

Thank you for your kind reminder. The temperatures shown in **Fig. 3 b** and **Fig. 3 c** represent the maximum temperature within the corresponding regions. We have clarified this in the relevant paragraph and in the caption of **Fig. 3 b** and **Fig. 3 c**.

Relevant revised content

Fig. 3, Page 8, Main Text-Clean Version

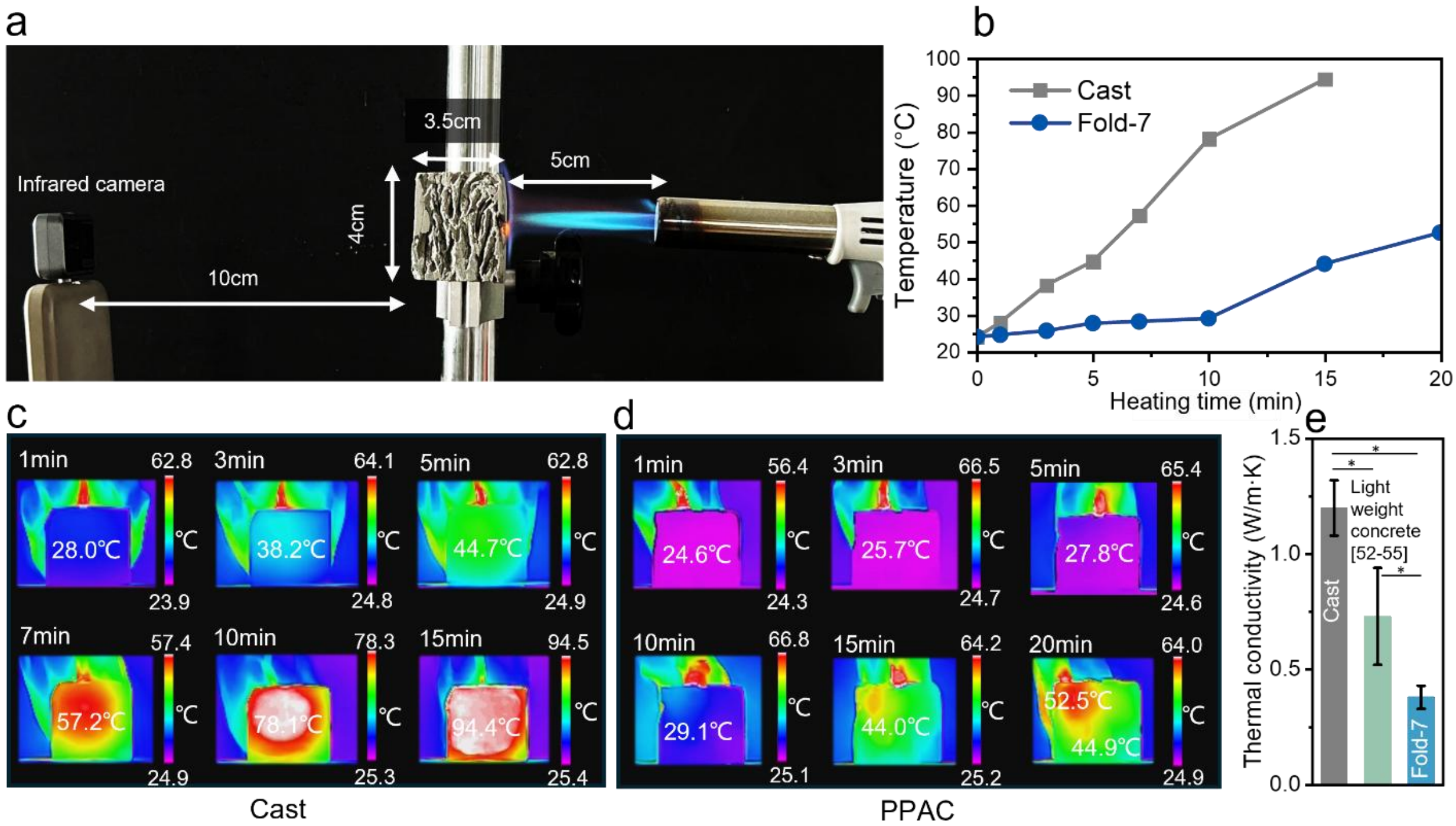

Fig. 3 Thermal insulation performance of as-fabricated puff pastry architected cementitious material (PPAC). a Illustration of the fire & thermal resistance test configuration. **b** Maximum temperature evolution of specimen's backside. **c, d** Thermal infrared images of the specimen's backside during the fire & thermal resistance test. The values shown in the figure represent the maximum temperature in the corresponding regions. **e** Comparison on thermal conductivity of cast specimen, light weight concrete possessing density similar to PPAC Fold-7, and PPAC Fold-7. The data of lightweight concrete are referenced from the literatures [52-55].

10. In this study, the samples are fabricated using cement paste; however, the large infrastructure materials are fabricated using mortar or concrete. Concrete/mortar potentially shows complex plastic behavior due to the presence of aggregates, which may impact the fabrication process used in this study. Can you address the potential extension of the fabrication process to mortar and concrete? In addition, adding brief comments in the Conclusion section on scalability to infrastructure applications, including potential use of robotics or hybrid additive manufacturing approaches, could be beneficial.

Response:

We sincerely thank you for your insightful and highly valuable comments. To our consideration, the incorporation of sand or coarse aggregates may influence the rheological properties of the material, which may introduce challenges during processing. However, the rolling–folding process employed in this study does not rely on operations at very fine length scales. Moreover, the formation of weak interfaces is not directly dependent on the specific composition of the paste material. Therefore, we believe that materials prepared under such conditions would still retain the key structural features of PPAC, namely the layered architecture and the presence of discrete weak interfaces. In fact, we have conducted a preliminary exploration of fabricating PPAC using mortar, as presented in **Supplementary discussion 1**. However, the size and shape of aggregates (e.g., sand or gravel) may indeed affect characteristics such as the thickness of the solid layers and

the tortuosity of the microstructure. These effects warrant more in-depth and systematic investigation. To address these points, we have added a "**Discussion and open questions**" section at the end of the manuscript to clarify the limitations of the present study and outline the challenges for practical implementation. The extension of PPAC to mortar and concrete systems is explicitly discussed. In addition, as per your suggestion, we also provide a perspective on the potential for industrial-scale production of PPAC through the integration of robotic technologies and additive manufacturing approaches.

Relevant revised content

Discussion and open questions, Page 14~15, Main text-Clean Version.

"This study proposes an innovative and facile fabrication method for a novel puff pastry architected cementitious material (PPAC) that simultaneously achieve enhanced toughness and thermal insulation, while remaining scalable for large-size production. The significance of this concept lies in economically enhancing the safety and comfort of urban environments by integrating high mechanical toughness, effective thermal insulation, and a scalable while cost-effective fabrication process under ambient conditions.

Despite the demonstrated performance advantages and application potential of PPAC, this work represents only an initial step toward its large-scale implementation in real construction scenarios, and many questions remain to be addressed. For example, while SA-crosslinked hydrogels have been shown to effectively introduce weak interfaces in cementitious materials, their physicochemical properties and the influence of their tunability on interfacial morphology require further investigation. Moreover, other materials capable of generating similar weak interfaces may exist, offering opportunities to expand the PPAC concept and potentially introduce additional functionalities. In addition, considering the complex service conditions of infrastructure, more systematic and strategy-driven studies are needed, such as compressive performance optimization (**Supplementary discussion 1**), size-dependent behavior (**Supplementary discussion 2**), customized structural design, and long-term durability (**Supplementary discussion 3**).

To translate this concept from laboratory-scale demonstration to practical applications, our future work will focus on both structural and material optimization. In particular, considering the carbon footprint of cementitious systems and the widespread use of aggregates in real construction, we will explore to extend the PPAC concept to mortar, concrete, and even fiber-reinforced composites.

Furthermore, one of the key advantages of PPAC is its mild processing conditions, simple fabrication procedure, and low cost (**Supplementary discussion 4**). The fabrication process used in this study is limited to laboratory-scale demonstrations and primarily serves as a proof of concept. From an industrial perspective, further optimization of the process is required to improve reproducibility and ensure consistent product quality. According to the fundamental principle underlying PPAC fabrication, each step in the process has strong potential for automation. The integration of robotic assistance, additive manufacturing techniques and industrial automation systems could enable the development of efficient and scalable production lines for PPAC.

Nevertheless, this study establishes a proof-of-concept for PPAC with combined high toughness and thermal insulation, providing a new insight for the development of high performance and multifunctional infrastructure materials. With continued advances, PPAC may enable cost-effective, energy saving and scalable manufacturing of infrastructure systems that simultaneously offer enhanced safety and efficient thermal management, thereby improving the comfort and resilience of human living and working environments."

11. The viscosity value (75000) in Supplementary Line 24 and Line 282 is missing a unit, and there is a typo

in Supplementary Line 24: "power" should be "powder." Please review the manuscript for proofreading.

Response:

Thank you very much for your careful review and kind reminder. We sincerely apologize for these errors. The unit of the viscosity you pointed out is Pa·s, and we have added this information in the relevant sections. We have also corrected the typographical errors. In addition, we have carefully rechecked the entire manuscript once again to avoid such issues.

Reviewer 3

The manuscript presents a novel and conceptually strong approach supported by substantial experimental evidence and with clear potential impact. However, key aspects related to durability, broader mechanical characterization, scalability, and the limitations of thermal testing and modeling require deeper discussion and clarification before the work can be accepted. The study is promising and likely publishable after substantial but feasible revisions.

Recommendation: Major Revision

The manuscript presents a novel puff pastry architected cementitious material (PPAC) fabricated via a simple rolling–folding process conducted entirely under ambient conditions. Inspired by laminated pastry processing, the approach employs in situ crosslinked sodium alginate to generate discrete weak interfaces that, after fragmentation and drying, form a lamellar architecture with embedded cavities. The material exhibits markedly enhanced specific fracture toughness (≈ 90 MPa·mm^0.5/g·cm^3), pronounced ductile behavior, and significantly reduced thermal conductivity (0.38 W/m·K), while maintaining comparable specific strength to cast cement paste. The concept is original, experimentally well supported, and potentially impactful for multifunctional infrastructure materials.

The experimental program is comprehensive, combining 3PB and SENB testing, R-curve analysis with DIC monitoring, X-CT and SEM/EDS characterization, flame-based thermal testing, and multiphysics heat-transfer modeling. The correlation between architecture, crack deflection, mechanical interlocking, and macroscopic toughening is convincingly demonstrated.

However, several issues require clarification. First, claims regarding scalability and infrastructure applicability would benefit from a more rigorous discussion of process reproducibility, dimensional control, and industrial feasibility. Second, mechanical characterization is limited to monotonic flexure; compressive behavior, fatigue resistance, and durability (e.g., permeability, freeze–thaw resistance, and the chemical stability of alginate films in alkaline environments) are not addressed. Third, the flame test is illustrative but not standardized for structural fire resistance, and potential thermal anisotropy is not quantified. Finally, the thermal model relies on geometric simplifications whose limitations should be more explicitly acknowledged.

Overall, the study introduces a compelling and energy-efficient architectural strategy for cementitious materials, achieving substantial improvements in toughness and thermal insulation. With a strengthened discussion of durability, scalability, and testing limitations, the manuscript would represent a significant contribution to architected cement-based materials.

Response:

We sincerely thank you for your positive evaluation of this study and for all the constructive comments you have provided. We believe that each of your suggestions is highly insightful and of great significance for improving the quality of this work as well as guiding its future development. We respond to your comments from several aspects, as outlined below.

Industrial feasibility: Regarding the reproducibility, dimensional control, and industrial feasibility of the proposed process, we have made additional consideration and discussion. However, considering that the main text is intended to emphasize the core concept, innovation, and scientific insights, these relevant contents have been included in the **Supplementary discussion 4**. In this section, we elaborate on the efficiency of PPAC fabrication, the stability of the processing workflow, the feasibility and the future industrial-scale implementation.

Compressive properties: Compressive strength is indeed an important property for infrastructure applications. Therefore, we have conducted additional uniaxial compression tests. The results show that, due to the presence of weak interfaces, the compressive performance of PPAC is lower than that of the cast counterpart. Nevertheless, we found that the compressive strength can be effectively improved by adjusting the material composition. In addition, PPAC exhibits pronounced anisotropy under compression, which is mainly attributed to its highly ordered architecture and the presence of weak interfaces. This observation is particularly interesting and introduces unique considerations for the structural design of PPAC. The full set of results and discussion is provided in **Supplementary Discussion 1.** However, it should be noted that this study primarily focuses on demonstrating the unique advantages of PPAC in terms of toughness and thermal insulation. We admit that several aspects related to practical engineering applications still require further optimization, but these questions may need additional research to address. In future work, we will aim to improve the compressive performance of PPAC through combined structural design and material optimization, and to maximize its overall performance through rational structural design strategies.

Durability: Regarding durability, we greatly appreciate your recognition of the core concept and your forward-looking perspective on large-scale applications. Based on the unique architecture of PPAC, durability is indeed an important issue that warrants careful consideration. The durability of cementitious materials is fundamentally governed by moisture ingress. In PPAC, the presence of weak interfaces may serve as preferential pathways for moisture transport, potentially facilitating the penetration of deleterious agents and leading to freeze–thaw damage. This issue is therefore of great relevance to the long-term engineering application of PPAC. However, due to the scope of the present study, a systematic investigation of PPAC durability is not included in the main text. We fully appreciate your comment on this aspect. Based on the underlying mechanism of moisture transport, we hypothesize that hydrophobic protection could be an effective strategy for enhancing the durability of PPAC. Accordingly, we have added a brief discussion in **Supplementary Discussion 3**, where we explore the feasibility of hydrophobic treatment to mitigate water ingress into PPAC. We hope this addition provides a meaningful complement to the manuscript and offers a preliminary perspective on potential strategies for addressing the durability challenges of PPAC.

Thermal performance: Regarding fire resistance and thermal insulation, we acknowledge that the flame

torch heating combined with infrared imaging presented in the manuscript serves primarily as a qualitative demonstration. We have clarified this in **Lines 198~201, Page 8, Main text**. Furthermore, we have investigated the thermal anisotropy of PPAC and the results are presented in **Supplementary Fig. 15,** with a brief description added to the main text (**Lines 188~190, Page 7~8, Main text**). We speculated that the mechanism is similar to those reported in Ref. [10.1002/adma.202506308]. Meanwhile, the simplifications applied in the thermal simulation model and its limitation are clearly described in **Lines 249~257, Page 9~10, Main text**.

Limitations: Based on your comments, we recognize that the limitations and challenges for the current proposed PPAC concept, including constraints in the experimental methods and simplifications in the modeling approach. After all, this study focuses primarily on concept development and validation, and many challenges remain before practical engineering applications can be realized. These include further optimization of material performance, extension of the PPAC fabrication process to mortar or concrete systems, development of compatible structural designs, and advancement toward industrial-scale production. To address these aspects, we have added a "**Discussion and open questions**" section at the end of the manuscript to outline the limitations of this work and potential directions for future research.

Once again, we sincerely thank you for your careful review and valuable comments. We hope that the revised manuscript meets your expectations more effectively.

Relevant revised content

Lines 188~190, Page 7~8, Main Text-Clean Version

"The lamellar structure of PPAC endows it with pronounced thermal anisotropy (**Supplementary Fig. 15**), and it exhibits excellent thermal insulation performance when the heat flow is perpendicular to the lamellar structure."

Lines 198~201, Page 8, Main Text-Clean Version

"It should be noted that this test did not follow a standardized procedure and was intended solely as a qualitative demonstration to visually illustrate the thermal insulation performance of PPAC. Therefore, the more precise and quantitative Hot Disk measurements were conducted……"

Lines 249~257, Page 9~10, Main Text-Clean Version

"During model construction, a simplified 2D model was employed (**Supplementary Fig. 30**). The weak cavities were idealized as rectangles with an aspect ratio of 10:1 based on the observations in **Fig. 4 b**, and the pore medium was defined as air. The pore area in the 2D model was determined according to the density of the Fold-7 specimen. A regularly staggered arrangement was then adopted to approximate the interlocking features and the dispersed weakness phase distribution. Although this simplified model was used, it retains the key structural characteristics of PPAC and incorporates appropriate parameterization. While the simulation is not intended for quantitative prediction, it still enables a directional comparison with the cast counterpart, thereby partially reflecting the thermal insulation advantage of PPAC."

Supplementary discussion 1. Compressive properties of PPAC. Supplementary Information.

Due to the presence of the internal sheet-like cavities, the compressive strength is lower than that of cast counterparts with the same mix proportion (**Supplementary Fig. 31 b**). In addition, owing to the pronounced orientation of its lamellar structure, the compressive strength of PPAC shows significant anisotropy between the in-plane and out- plane directions (**Supplementary Fig. 31 a**). Specifically, when the load is applied parallel to the weak interfaces and lamellar structure (in-plane direction), the compressive strength is markedly higher than when the load is applied perpendicular to the lamellar structure (out-plane direction). The underlying mechanism is reasonably straightforward. Under out-plane loading, stress concentration is more likely to occur at the tips of the thin interlayer cavities [19], and the interlocking regions between lamellae become the critical sites for failure. In contrast, when loading is applied in the in-plane direction, stress concentration within the sheet-like cavities is alleviated, and the lamellae can dominate the failure process, resulting in a larger effective load-bearing area (**Supplementary Fig. 31 a**), thereby leading to higher compressive strength (**Supplementary Fig. 31 b**). However, for the unique compressive behavior observed in PPAC, we only provide a preliminary interpretation based on the experimental results, and the underlying mechanisms require further investigation.

Considering that compressive strength is also important for infrastructure materials in practical engineering applications, we conducted preliminary investigations into several approaches to enhance compressive strength by adjusting material composition, include:

1. Mortar: Sand was introduced into the cement paste system. The sand used was fine quartz sand with a maximum particle size of approximately 0.6 mm, while all other components remained consistent with those used in the cement paste-based PPAC. The detailed mix proportions are as follows:

Supplementary Table 11. Mix proportion of PPAC-cement mortar (g/1000g binder)

Cement	Water	Sand	PCE	HPMC
1000	350	1500	3.5	20

2. UHPC mortar: PPAC was fabricated using UHPC mortar without steel fibers. Based on the previously described cement mortar system, silica fume and slag were further incorporated. The detailed mix proportions are as follows:

Supplementary Table 12. Mix proportion of PPAC-UHPC mortar (g/1000g binder)

Cement	Silica fume	Fly ash	PCE	Water	Sand
680	140	180	14	170	900

The experimental results indicate that the utilization of UHPC mortar can effectively enhance the compressive strength of PPAC, especially when loading is applied along the in-plane direction (**Supplementary Fig. 31 b**). The higher strength in the in-plane direction may be advantageous for certain engineering applications (**Supplementary Fig. 31 c**). For example, when used as a wall material to exploit its thermal insulation properties, the lamellar voids would be oriented perpendicular to the ground, and the primary gravitational load would be applied parallel to the weak interfaces. In contrast, when subjected to out-plane loading, such as in roofs or slabs, the imposed loads (e.g., human activity or snow loads) are typically much smaller than the self-weight of the structure, and structural performance is more strongly governed by flexural strength.

These results demonstrate the tunability and controllability of PPAC, as well as its potential for further performance enhancement. However, it should be noted that the primary objective of this study is to propose the underlying concept. More detailed optimization strategies and material design considerations will require more comprehensive and systematic investigations in future work.

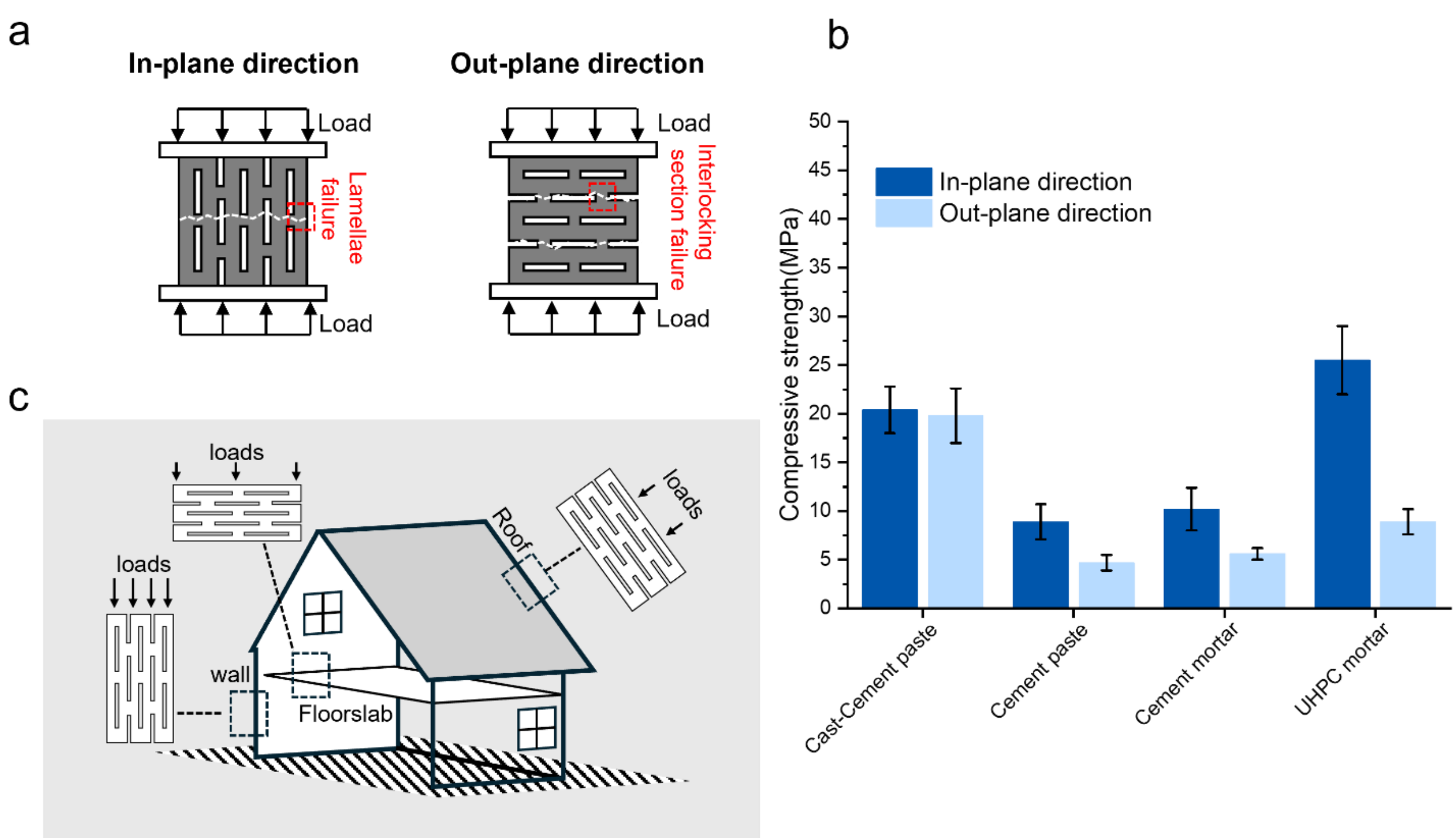

Supplementary Fig. 31. Compressive strength of PPAC from different loading directions. a Schematic illustration of in-plane and out-plane loading directions. **b** Compressive strength test results of PPAC specimens fabricated with different paste compositions. Specimens with dimensions of about 40mm×40mm×40mm were used. The Folding-time of these specimens are 7. **c** Schematic diagram of potential loading situations in housing applications.

Supplementary discussion 3. Durability of PPAC. Supplementary Information.

Considering the long-term service conditions of infrastructure, durability is a critical property of cementitious materials. Moisture ingress is one of the primary causes of durability-related degradation[22]. In PPAC, the presence of numerous weak interfaces can facilitate water transport, as these interfaces may act as preferential pathways for moisture diffusion (**Supplementary Fig. 33 a**). This study primarily aims to establish and validate the toughness and thermal insulation performance of PPAC, and thus does not present a systematic investigation of durability. However, here we offer preliminary insights and explore the feasibility of potential strategies for improving its durability. Surface hydrophobic treatment is a commonly adopted and effective strategy to improve the durability of cementitious materials[23, 24]. We applied similar approach to PPAC. First, the PPAC specimens were dried to constant mass. Subsequently, a commercial sodium methyl silicate–based silane hydrophobic agent was uniformly applied to the specimen surfaces. After 24 hours, the PPAC surface exhibited pronounced hydrophobicity. Even in regions with weak interfaces, water droplets were effectively prevented from penetrating into the material (**Supplementary Fig. 33 b, c**). To quantitatively

evaluate water absorption, untreated cast specimens, untreated PPAC specimens, and treated PPAC specimens were dried and tested according to ASTM C1585. The specimens were placed on a support within a water reservoir, allowing one surface to be in contact with water, and the mass change was measured at specified time intervals to determine water uptake. The water exposure direction for PPAC was consistent with that shown in **Supplementary Fig. 33 c**. As shown in **Supplementary Fig. 33 d**, due to the presence of weak interfaces, PPAC exhibits a higher water absorption rate than the cast counterpart. However, after hydrophobic treatment, the water absorption of PPAC decreases remarkably and becomes notably lower than that of the cast specimens. These results demonstrate that hydrophobic protection can be an effective strategy for enhancing the durability of PPAC and provides a promising direction for its future development.

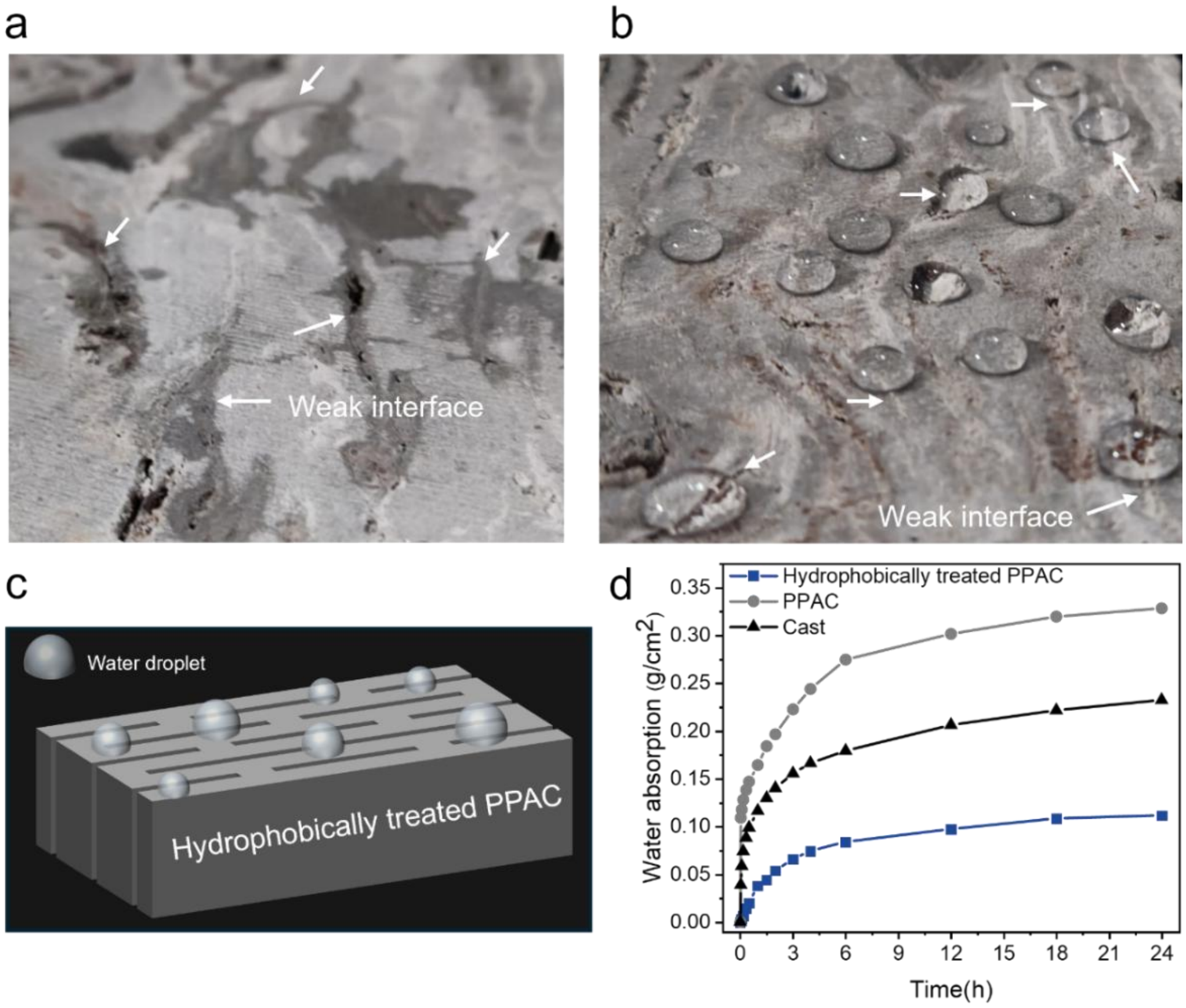

Supplementary Fig. 33. Preventing water ingress into PPAC through surface hydrophobic treatment. a Water droplet absorption on the PPAC surface. **b** hydrophobically treated PPAC surface. **c** Schematic illustration of a water droplet on the hydrophobically treated PPAC surface. **d** Water absorption test results.

Supplementary discussion 4. Evaluation of processing simplicity and Industrial feasibility. Supplementary Information.

Reproducibility: The PPAC fabrication process proposed in this study mainly consists of four steps: preparation of the sodium alginate solution, cement paste mixing, rolling–folding, and curing. The materials used in PPAC are all conventional constituents in cement-based materials and commercially available industrial products. No complex pre-treatment of raw materials is required during processing, which ensures that the material composition can be readily fixed and remains stable across repeated production.

The equipment involved in this study includes a mixer for preparing the sodium alginate solution, a mixer

for preparing cement paste, and a machine for the rolling–folding process, both of which are relatively low-cost. The preparation of the sodium alginate solution involves a simple powder dissolution and stirring process without the need for heating, making it readily scalable for industrial production. Similarly, cement paste mixing is a well-established and robust process, and the reproducibility can be ensured by using standardized mixing equipment and maintaining consistent mixing durations.

However, we acknowledge that the rolling-folding process presents certain challenges in terms of reproducibility and stability, but these challenges may be addressed by industrial design and optimization. In this study, due to limitations of the available equipment, several steps in the rolling process were performed manually. Extending the fabrication process in this study to an industrial-scale operational workflow could significantly improve process stability.

Overall, the PPAC fabrication process does not involve any intrinsically difficult-to-control physicochemical processes, such as freeze–thaw cycling, pore-forming, or granulation. Given that the formation of the lamellar structure in PPAC is governed by a relatively simple underlying mechanism, its fabrication is expected to benefit significantly from mechanization and automation, thereby enhancing reproducibility and controllability in large-scale production.

Dimensional Control: The dimensional control of PPAC is relatively straightforward. The thickness of PPAC can be precisely regulated by adjusting the distance between the rollers and the base plate in the rolling equipment. For the fabrication of larger structural elements, such as wall panels, slabs or beams, this can be achieved either by employing larger-scale rolling equipment or by adopting rolling-folding equipment with larger size or using assembly strategy at the plastic stage of PPAC. Given the inherently straightforward underlying mechanisms of the PPAC fabrication process, the development of more precise and controllable large-scale manufacturing approaches in industrial settings is expected to be feasible, primarily relying on equipment advancement and process standardization.

Potential Industrial production line: Based on the PPAC fabrication process, industrial production line can be speculated with the aid of automatic and robotic equipment. For example, during the rolling-folding process, the roller height and motion can be precisely and automatically controlled by motors. The spraying of sodium alginate (SA) solution on material surface can be automatically accomplished using a robotic arm. Subsequently, the material can be subjected to cutting and folding operations using robotic arms or other automated mechanisms. Then, the material can be returned to the rolling stage, where the aforementioned sequence—SA spraying, cutting, and folding—can be repeated. By controlling the number of production cycles, plastic PPAC with a designed number of lamellar interfaces can be fabricated.

Discussion and open questions, Page 14~15, Main text-Clean Version.

"This study proposes an innovative and facile fabrication method for a novel puff pastry architected cementitious material (PPAC) that simultaneously achieve enhanced toughness and thermal insulation, while remaining scalable for large-size production. The significance of this concept lies in economically enhancing the safety and comfort of urban environments by integrating high mechanical toughness, effective thermal insulation, and a scalable while cost-effective fabrication process under ambient conditions.

Despite the demonstrated performance advantages and application potential of PPAC, this work represents only an initial step toward its large-scale implementation in real construction scenarios, and many questions remain to be addressed. For example, while SA-crosslinked hydrogels have been shown to

effectively introduce weak interfaces in cementitious materials, their physicochemical properties and the influence of their tunability on interfacial morphology require further investigation. Moreover, other materials capable of generating similar weak interfaces may exist, offering opportunities to expand the PPAC concept and potentially introduce additional functionalities. In addition, considering the complex service conditions of infrastructure, more systematic and strategy-driven studies are needed, such as compressive performance optimization (**Supplementary discussion 1**), size-dependent behavior (**Supplementary discussion 2**), customized structural design, and long-term durability (**Supplementary discussion 3**).

To translate this concept from laboratory-scale demonstration to practical applications, our future work will focus on both material and structural optimization. In particular, considering the carbon footprint of cementitious systems and the widespread use of aggregates in real construction, we will explore to extend the PPAC concept to mortar, concrete, and even fiber-reinforced composites.

Furthermore, one of the key advantages of PPAC is its mild processing conditions, simple fabrication procedure, and low cost (**Supplementary discussion 4**). The fabrication process used in this study is limited to laboratory-scale demonstrations and primarily serves as a proof of concept. From an industrial perspective, further optimization of the process is required to improve reproducibility and ensure consistent product quality. According to the fundamental principle underlying PPAC fabrication, each step in the process has strong potential for automation. The integration of robotic assistance, additive manufacturing techniques and industrial automation systems could enable the development of efficient and scalable production lines for PPAC.

Nevertheless, this study establishes a proof-of-concept for PPAC with combined high toughness and thermal insulation, providing a new insight for the development of high performance and multifunctional infrastructure materials. With continued advances, PPAC may enable cost-effective, energy saving and scalable manufacturing of infrastructure systems that simultaneously offer enhanced safety and efficient thermal management, thereby improving the comfort and resilience of human living and working environments.”

Response to reviewers

Manuscript title: Tough, thermal insulating and scalable puff pastry architected cementitious material via simple rolling-folding

Manuscript Number: NCOMMS-25-92106A

Authors: Kailun Xia, Yuning Chen, Xingzi Liu, Siyuan Qiu, Qian Lin, Yi Tang, Tiantian Xiang, Sule Chen, Enlai Dong, Zijian Jia, Wei Wang, Yu Chen, Yamei Zhang*

Dear Editor and reviewers:

We sincerely appreciate your valuable time and effort for carefully reviewing this manuscript. We have conscientiously considered all the comments and made corresponding modifications and revisions. We believe these comments can help us improve our manuscript a lot. The reviewers' comments are listed point by point with corresponding responses. For the convenience of reviewers and editor, the corresponding modifications have been appended beneath each response, thus you can directly see these modifications. For your convenience. **Please Note:** For the convenience of the editor and reviewers, and to accommodate potential differences in review preferences, we have provided both a **marked-up manuscript with tracked changes** and a **clean version**. **All line numbers and references mentioned in the responses below correspond to the clean version of the manuscript.**

We are looking forward to your kind consideration of the suitability for accepting this manuscript after accomplishing the revision. If there are any further questions, please let us know at your convenience.

Best regards

Yamei Zhang,

Corresponding author, on behalf of all the authors

Reviewer #1

I am satisfied with the changes the authors have made in the revised document. All my comments and questions were addressed. The changes are substantial and add value and greater insight. This is a high quality and interesting study, now worthy of publication in this journal.

Response:

We sincerely appreciate your positive evaluation of our manuscript. We are pleased that the revisions we made have satisfactorily addressed your concerns. Your constructive comments and suggestions have significantly contributed to improving the scientific rigor and overall quality of this study. We are truly grateful for the time and effort you devoted to reviewing our work.

Reviewer #2

The authors have addressed my comments and incorporated the corresponding revisions. Therefore, I recommend publication of the manuscript, subject to the following minor comment, which should be addressed:

- Please include the units of the reported properties in Supplementary Tables 13 and 14. In addition, please clarify whether the significance analysis was performed for specific KIC and KJC values or for KIC and KJC.

Response:

We sincerely thank you for your positive evaluation and recognition of our manuscript. We also greatly appreciate your constructive comments, which have been instrumental in improving the quality of our work. Regarding your minor comment, we have added the corresponding units to Supplementary Table 13 and Supplementary Table 14. In addition, all significance analyses in this study were conducted based on the data presented in the figures and tables. In other words, the significance analysis was performed for the specific K_{IC} and specific K_{JC} values. In addition, corresponding annotations have been added to the tables for clarification.

Relevant content

Supplementary Table 13-14

Supplementary Table 13. Data set and t-test significance analysis results of the Specific K_{IC} ($MPa \cdot mm^{0.5}/g \cdot cm^3$) results in Supplementary Fig. 32

Sub Table a. Data set

Specimen	Mean value (Specific K_{IC})	SD	Size (n)
20mm	4.47599	0.29	3
40mm	5.10306	0.38	3
60mm	4.74513	0.28	3

Sub Table b. Significance analysis

Comparison	Mean difference	T-value	df	p-value	Significance
40 mm vs 20 mm	0.62707	2.27	3	0.11	n.s.
60 mm vs 20 mm	0.26914	1.15	3	0.33	n.s.
40 mm vs 60 mm	0.35793	1.32	3	0.27	n.s.

* ($p<0.05$) indicates a significant difference; ** ($p<0.01$) indicates a highly significant difference; n.s. means no significant difference.

Supplementary Table 14. Data set and t-test significance analysis results of the Specific K_{JC}-failure ($MPa \cdot mm^{0.5}/g \cdot cm^3$) results in Supplementary Fig. 32

Sub Table a. Data set

Specimen	Mean value (Specific K_{JC} at failure)	SD	Size (n)
20mm	114.6	10.02	3
40mm	170.67	14.82	3
60mm	144.59	11.07	3

Sub Table b. Significance analysis

Comparison	Mean difference	T-value	df	p-value	Significance
40 mm vs 20 mm	56.07	5.42	3	0.012	*
60 mm vs 20 mm	29.99	3.47	4	0.026	*
40 mm vs 60 mm	26.08	2.44	3	0.09	n.s.

* ($p<0.05$) indicates a significant difference; ** ($p<0.01$) indicates a highly significant difference; n.s. means no significant difference.
